# Regarding the existence of abelian fractional topological insulators in twisted MoTe_2_ and related systems

**DOI:** 10.1038/s42005-025-02483-6

**Published:** 2026-01-16

**Authors:** Yves H. Kwan, Glenn Wagner, Jiabin Yu, Andrea Kouta Dagnino, Yi Jiang, Xiaodong Xu, B. Andrei Bernevig, Titus Neupert, Nicolas Regnault

**Affiliations:** 1 Department of Physics, University of Texas at Dallas, Richardson, TX USA; 2 Department of Physics, University of Zürich, Zürich, Switzerland; 3 Institute for Theoretical Physics, ETH Zürich, Zürich, Switzerland; 4 Department of Physics, Princeton University, Princeton, NJ USA; 5 Donostia International Physics Center, P. Manuel de Lardizabal 4, Donostia-San Sebastian, Spain; 6 Department of Materials Science and Engineering University of Washington, Seattle, WA USA; 7 Department of Physics, University of Washington, Seattle, WA USA; 8 IKERBASQUE, Basque Foundation for Science, Bilbao, Spain; 9 Laboratoire de Physique de l’Ecole normale superieure, ENS, Universite PSL, CNRS, Sorbonne Universite, Universite Paris-Diderot, Sorbonne Paris Cite, Paris, France; 10 Center for Computational Quantum Physics, Flatiron Institute, New York, NY USA

**Keywords:** Topological matter, Quantum Hall

## Abstract

The experimental discovery of fractional Chern insulators (FCIs) in moiré materials raises the question of whether their time-reversal invariant analogs, fractional topological insulators (FTIs), can also be realized in these platforms. We address this via exact diagonalization calculations in both a Landau level (LL) model and continuum model for twisted MoTe_2_, and extract principles for engineering FTIs in realistic conditions. For the spinful LL model at filling $\nu =\frac{1}{3}+\frac{1}{3}$, we show that a suppression of the short-range component of the interaction is important to stabilize the FTI. For twisted MoTe_2_ at $\nu =-\frac{4}{3}$, we find that a short-range attraction *g* on top of the screened Coulomb interaction is needed to realize an FTI. We discuss how this threshold value of *g* could be reduced by examining larger system sizes, incorporating band-mixing effects, exploiting Landau level character, and engineering the dielectric environment. While our study highlights the challenges, at least for the fillings considered, for obtaining FTIs, we also provide potential sample-engineering routes to improve the stability of FTI phases.

## Abstract (web summary)

Fractional Chern insulators, and their time-reversal analogs, fractional topological insulators, are realizations of topological order in flat-band electronic systems; while the former have been realized experimentally in twisted bilayer MoTe_2_, the latter have remained more elusive. Here, using exact diagonalization calculations, the authors propose routes towards engineering fractional topological insulators in twisted bilayer MoTe_2_ and other moiré materials.

## Introduction

Fractional Chern insulators (FCIs)^1–3^ are the zero-field analog of the venerable fractional quantum Hall (FQH) effect that appear in fractionally filled narrow Chern bands^4–6^. The interactions between the electrons lead to a state with fractionalized excitations, a fractionally quantized Hall conductivity and a topological ground state degeneracy. Moiré materials are known to host flat topological bands with relatively uniform Berry curvature, making them ideal platforms for realizing FCIs.

Recent experiments on twisted homobilayer MoTe_2_ (*t*MoTe_2_) have observed direct consequences of an FCI^7,8^, especially a fractionally quantized Hall conductance at filling factors *ν* = − 2/3 and  − 3/5^9–12^. Although exact diagonalization (ED) calculations without band mixing can yield FCIs at certain fillings^13–18^, inclusion of band mixing^19–21^ is essential for theoretically capturing key aspects of the experimental phenomenology. Furthermore, quantitative comparison to experiments requires careful consideration of the band structure^22–25^. Even more recently, pentalayer graphene with a hexagonal boron nitride (hBN) substrate has been experimentally shown to host a Chern insulator at *ν* = 1^26,27^ and FCIs at multiple fillings *ν* < 1^27^, which have motivated several recent theoretical studies^28–33^, though the appearance of an FCI is still not reproduced in unbiased calculations that account for multi-band effects.

A class of related topologically ordered phases are fractional topological insulators (FTIs), which are distinguished from FCIs in that they are not chiral, but time-reversal (TR) symmetric^34–39^. If spin is a good quantum number, these states exhibit the fractional quantum spin Hall effect (FQSHE). However, neither spin conservation nor TR symmetry are strictly required to protect the topological order, i.e. the anyon content, of these phases, in sharp contrast to *Z*_2_ topological insulators which require TR symmetry. Both FTI and FQSHE physics represent a substantial departure from FQH physics, and are still experimentally elusive, although there exists a recent report of a potential FQSHE in low-angle *t*MoTe_2_^40^. Indeed, FTIs have a vanishing Hall response, while also supporting fractionally charged excitations and a topological ground state degeneracy. The simplest FTIs (which we will be concerned with in this work) are adiabatically connected to a direct product of a Laughlin state for spin-up electrons and its time-reversed copy for spin-down electrons. They can be constructed by partially filling two topological bands with spin-locked Chern numbers *C* = ± 1. FTIs have been numerically investigated via ED in both lattice toy models^36,41–43^ and lowest Landau level (LLL) models^44,45^. However, no theoretical proposal for an FTI with a material-realistic model has been put forward to date.

**Fig. 1:**
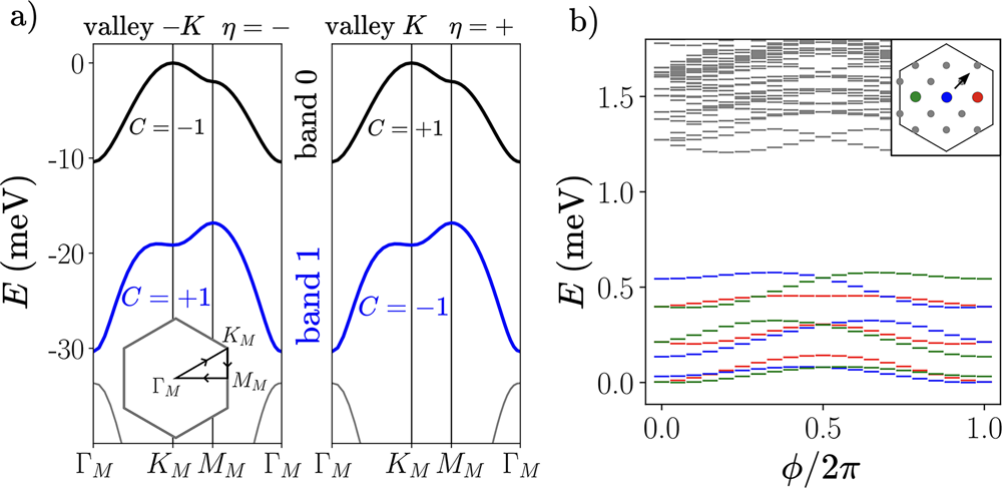
Twisted MoTe_2_ at twist angle 3. 7^∘^. **a** Continuum model band structure of the first-harmonic model of ref. ^22^. The highest valence bands have non-trivial valley Chern numbers. Inset shows the corresponding path in the moiré Brillouin zone (mBZ). **b** Many-body spectrum for *ν* = − 4/3, *S*_*z*_ = 0, and ${N}_{\max }^{1}=0$ as a function of flux *ϕ* threaded along one handle of the torus (inset shows momentum grid of the *N*_*s*_ = 15 lattice and shift under one flux insertion). Colored markers denote the lowest three states in each of the three momentum sectors (colored in inset) belonging to the fractional topological insulator’s ground state manifold. The long-range interaction is generated by solving the Poisson equation in the dual-gated device geometry using ${\epsilon }_{{{{{\rm{MoTe}}}}}_{2}}^{\perp }=10$, ${\epsilon }_{{{{{\rm{MoTe}}}}}_{2}}^{\parallel }=21$. In this figure, we add an additional short-range interaction with strength *g* = − 1400 meVnm^2^ (Eq. (3)). The FTI survives until *g* = − 1200 meVnm^2^ for these parameters.

The isolated valley Chern bands (Fig. 1a) and experimental signatures of FCIs in *t*MoTe_2_ naturally suggest the possibility of realizing a *ν* = − 4/3 FTI in this platform, namely by combining time-reversed copies of the experimentally-observed *ν* = − 2/3 FCI (at twist angle *θ* ≈ 3.7°) in opposite valleys (and hence opposite spins due to spin-valley locking), but several potential obstacles need to be addressed. First, a prerequisite for obtaining FTIs is the absence of magnetic order, while mean-field and single-band ED studies routinely find spin-valley polarization at *ν* = − 4/3, in contradiction with experiments. Ref. ^19^ shows that band mixing favors states with small magnetization at *ν* = − 4/3, where the ground state still has non-zero spin due to finite-size effects. Second, an FTI phase needs to be obtained with physical intervalley interactions. For vanishing intervalley interactions, the existence of the FTI in the spin-unpolarized sector as a product of two decoupled FCIs follows directly from the existence of the latter. However, numerical studies in other idealized models consistently find that FTIs are rapidly destabilized already by a moderate intervalley interaction^36,41^, calling into question the feasibility of FTIs in realistic *t*MoTe_2_ where the dominant interactions are expected to be valley-isotropic.

In this paper, we address these challenges in the twisted TMD homobilayer material platform. First working in the Landau level setting, we find that the FTI phase exists for valley-isotropic interactions when (i) the effect of non-local screening of the Coulomb interaction is accounted for and (ii) the short-range repulsion is sufficiently softened. We translate these insights to the realistic material setting by performing comprehensive ED calculations at *ν* = − 4/3 *t*MoTe_2_ around *θ* = 3.7°. We investigate how factors such as screening, short-range interactions, band structure parameters and band mixing influence the magnetism of the system and the stability of the FTI. The main conclusion we draw from our *t*MoTe_2_ calculations is that the FTI requires an additional onsite attraction, whose value is significantly larger than the microscopic contribution derived from electron coupling to monolayer phonons, to counteract the strong Coulomb repulsion at short distances.

## Results

### LLL model

**Fig. 2:**
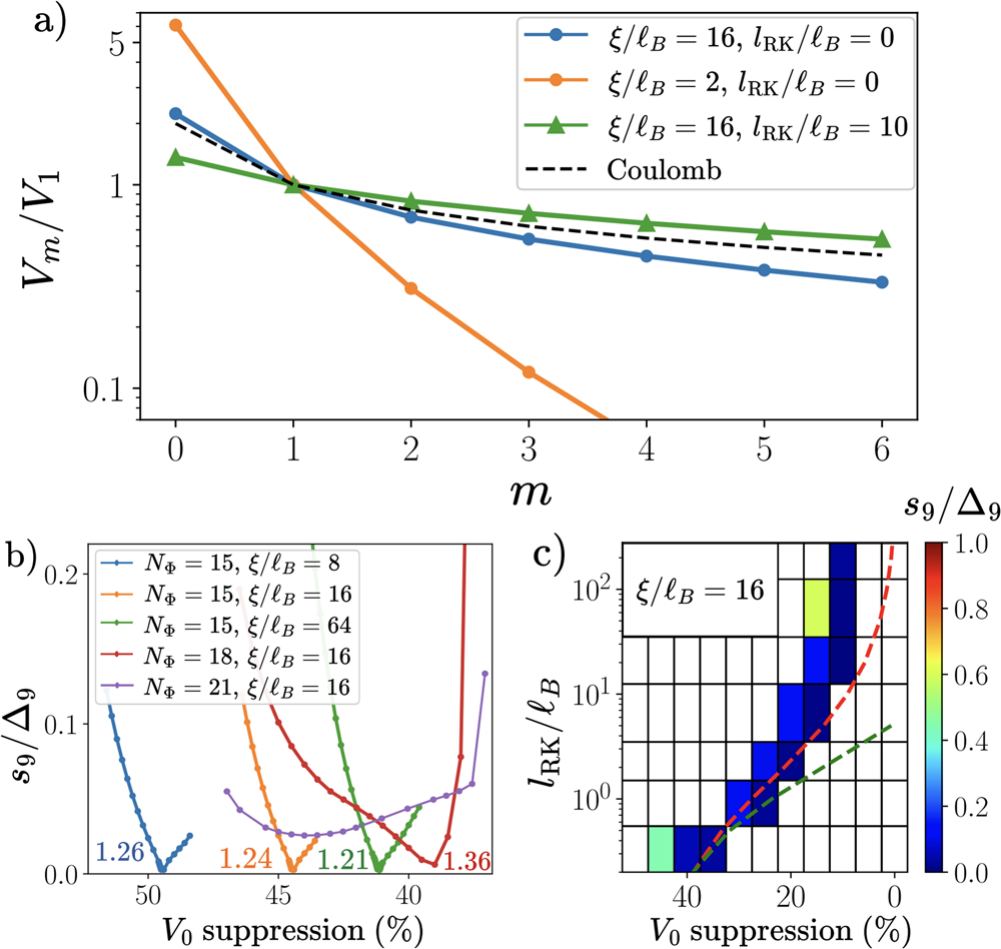
Time-reversal symmetric lowest Landau level model on torus geometry at 1/3 filling per valley. **a** Haldane pseudopotentials *V*_*m*_ for the Coulomb potential with dual-gate distance *ξ* and Rytova-Keldysh length scale *l*_RK_. Black dashed line indicates the bare Coulomb limit *ξ* → *∞*,  *l*_RK_ = 0. **b** Fractional topological insulator (FTI) spacing/gap ratio for different *ξ* and number of flux quanta *N*_*Φ*_. The horizontal axis shows the percentage suppression of *V*_0_. The number next to each curve denotes the value of *V*_0_/*V*_1_ corresponding to the minimum spacing/gap ratio. **c** FTI spacing/gap ratio (color) as a function of *l*_RK_ and *V*_0_ suppression for fixed *ξ*/*ℓ*_*B*_ = 16 and *N*_*Φ*_ = 18. White regions indicate where *Δ*_9_ < 0 or *s*_9_/*Δ*_9_ > 1. Red (green) dashed line is contour of constant *V*_0_/*V*_1_ = 1.24 (*V*_0_/*V*_2_ = 1.77).

**Fig. 3:**
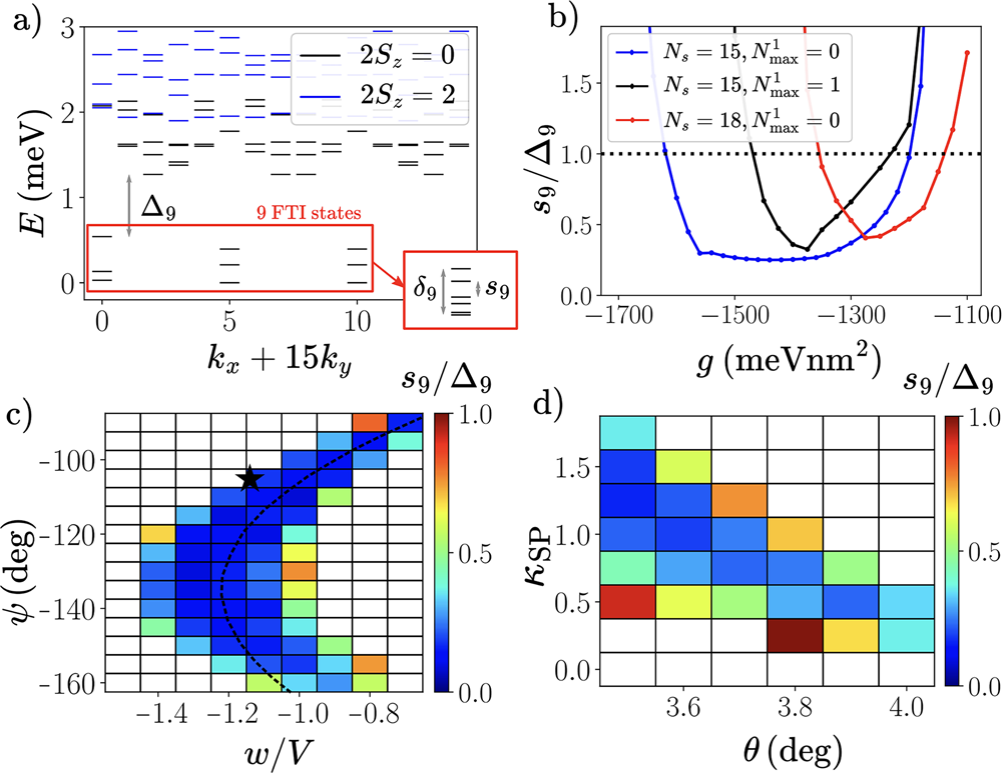
Exact diagonalization calculations of fractional topological insulators (FTI) in twisted MoTe_2_ at filling *ν* = − 4/3. Unless otherwise stated, calculations are performed at *θ* = 3. 7^∘^ with ${N}_{\max }^{1}=0$ for a tilted *N*_*s*_ = 15 lattice (see Fig. 1b inset). The interaction is generated by solving the Poisson equation in the dual-gated device geometry with ${\epsilon }_{{{{{\rm{MoTe}}}}}_{2}}^{\perp }=10$, ${\epsilon }_{{{{{\rm{MoTe}}}}}_{2}}^{\parallel }=21$. **a** Many-body spectrum of FTI phase. Parameters are identical to those of Fig. 1b, i.e., *g* = − 1400 meVnm^2^. Lowest four levels shown for each *S*_*z*_ and momentum sector. Higher spin sectors are above the energy axis limit. The nine states of the FTI manifold, indicated by red box, are separated by a gap *Δ*_9_ to the higher states. Zoom-in collapses the nine FTI states onto a single column, and indicates how the maximal spacing *s*_9_ and spread *δ*_9_ are defined. **b** Dependence of the FTI spacing/gap ratio as a function of *g* for different system sizes *N*_*s*_ and occupations ${N}_{\max }^{1}$ of band 1. The ground state is in the *S*_*z*_ = 0 sector for all data points. **c** *s*_9_/*Δ*_9_ (color) as a function of continuum model parameters *ψ* and *w*/*V* for fixed *g* = − 1400 meVnm^2^ and $\sqrt{{V}^{2}+{w}^{2}}=25\,{{{\rm{meV}}}}$. The black star indicates the continuum model parameters of ref. ^22^ used in this work. The dashed line tracks the minimum of the Berry curvature fluctuations as a function of *ψ*. **d** *s*_9_/*Δ*_9_ (color) as a function of bandwidth scaling factor *κ*_SP_ and twist angle *θ* for fixed *g* = − 1400 meVnm^2^.

To understand the FTI phase in a system that is free from the complications of dispersion, inhomogeneous band geometry/curvature and band mixing, we first consider a toy model with TR symmetry whose Hilbert space comprises two LLLs arising from opposite magnetic fields ${{{\bf{B}}}}=\eta B\widehat{z}$, where *η* = + ( − ) is the index for valley *K* ( − *K*)^42,44–57^. In the context of *t*MoTe_2_ with spin-valley locking, *η* also corresponds to the spin projection along *S*_*z*_. We add density-density interactions *V*(*q*) that preserve the valley-*U*_v_(1) symmetry, and define the Haldane pseudopotentials for relative angular momentum *m* (see Fig. 2a)

1 $${V}_{m}\equiv \int \frac{{d}^{2}{{{\bf{q}}}}}{{(2\pi )}^{2}}V(q){L}_{m}({q}^{2}){e}^{-{q}^{2}},$$

where *L*_*m*_(*x*) are the Laguerre polynomials and we have set the magnetic length *ℓ*_*B*_ = 1. The even *m* pseudopotentials do not affect the intravalley physics due to fermion antisymmetry. We perform ED calculations on the square torus geometry with *N*_*Φ*_ flux quanta, and fix the filling factors *ν*_*η*_ = *N*_*η*_/*N*_*Φ*_ = 1/3, where *N*_*η*_ is the number of particles in valley *η*. Note that the particle-hole symmetry within the LLL ensures that the results are identical for *ν*_*η*_ = 2/3 up to a global energy shift. Due to the periodic boundary conditions (PBCs), the FTI has nine topologically-degenerate ground states that are adiabatically connected to decoupled products of *ν* = 1/3 FQH states in the two valleys, and lie in specific momentum sectors^58^. The correct momentum-resolved counting of the FTI degeneracy can be straightforwardly inferred by performing a calculation where the intervalley interaction is artificially switched off. We characterize the FTI states through the maximum spacing *s*_9_ between adjacent levels in the FTI manifold, irrespective of the momentum, and the gap *Δ*_9_ that separates them from higher energy levels (see Fig. 3a). A smaller positive spacing/gap ratio *s*_9_/*Δ*_9_ indicates a more robust FTI. In this work, we use *s*_9_/*Δ*_9_ < 1 as a necessary condition for an FTI. A negative *Δ*_9_ means that the lowest nine states do not have the correct momenta for an FTI. More details of the conditions are given in Supplementary Note 1 A.

For a purely intravalley *V*_1_ interaction, there are nine zero-energy ground states corresponding to products of *ν* = 1/3 Laughlin states with no intervalley correlations. In ref. ^44^, the stability of this model FTI against finite *V*_0_ has been investigated. While the FTI still survived at *V*_0_ = *V*_1_, we emphasize that this does not correspond to a valley-isotropic interaction since the intervalley *V*_1_ was not considered. To our knowledge, there are no existing studies that have demonstrated an FTI phase for valley-isotropic (i.e., purely density-density) interactions in any fermionic system. Thus, we perform calculations in a restricted model with valley-isotropic *V*_0_ and *V*_1_ (see Fig. S3 in Supplementary Note 1 B), and find *s*_9_/*Δ*_9_ < 1 for the FTI in a window *V*_0_/*V*_1_ ≃ 1.18 − 1.35 (1.28 − 1.80) for *N*_*Φ*_ = 15 (18).

With an eye towards treating long-range interaction potentials relevant for moiré materials, we now consider the Coulomb potential screened by metallic gates positioned at height  ± *ξ*/2, i.e., $V(q)\propto \frac{\tanh \frac{q\xi }{2}}{q}$. The unscreened Coulomb potential has *V*_0_/*V*_1_ = 2 (Fig. 2a, dashed line), which is increased further by gate-screening (blue and orange lines). Since large *V*_0_/*V*_1_ may lead to phase-separated or disordered phases^44,45^, we introduce an additional short range attraction  ~ − *δ*(**r**) which only suppresses the *V*_0_ pseudopotential, and additionally helps to penalize ferromagnetism. While the resulting interaction has attractive components, the LLL-projected interaction remains purely repulsive as long as *V*_0_ > 0.

The spacing/gap ratio of the FTI as a function of the percentage suppression of *V*_0_ is shown in Fig. 2b, where we zoom in to highlight the small values of *s*_9_/*Δ*_9_, indicating very close degeneracy of the FTI ground states. It is clear that the FTI persists for a window around *V*_0_/*V*_1_ ≃ 1.3, similar to our isotropic *V*_0_ − *V*_1_ calculation mentioned above, despite the large-*m* tail of *V*_*m*_, whose decay rate decreases for weaker screening (Fig. 2a). The FTI is more stable for larger *ξ* in that the required suppression of *V*_0_ is reduced, and the best spacing/gap ratio in finite-size numerics is smaller (see Fig. S6 in Supplementary Note 1 C for *ξ*/*ℓ*_*B*_ = 2, 4 results). The latter is surprising since the valley-polarized *ν* = 1/3 Laughlin state only becomes exact in the *ξ* → 0 limit where *V*_*m*>1_/*V*_1_ → 0. This implies that the ideal conditions for realizing an FQH/FCI state do not necessarily coincide with those for an FTI. As the system size increases, there is a drift of the FTI phase towards greater *V*_0_/*V*_1_ (which implies less needed suppression of *V*_0_) for the system sizes studied. We note that for the largest calculations (*N*_*Φ*_ = 21), the *s*_9_/*Δ*_9_ curve has a slightly higher minimum (though still much less than 1) but is significantly broadened. This prevents us from performing a finite-size extrapolation to estimate the stability window of *V*_0_/*V*_1_ for the FTI in the thermodynamic limit. While the global ground state for the unsuppressed gate-screened interaction $V(q)\propto \frac{\tanh \frac{q\xi }{2}}{q}$ has finite valley polarization, the valley-unpolarized ${S}_{z}=\frac{1}{2}({N}_{+}-{N}_{-})=0$ sector is the lowest sector for values of *V*_0_/*V*_1_ where the FTI is stabilized (see Fig. S7 in Supplementary Note 1 C).

We now account for the Rytova-Keldysh (RK)^59,60^ correction to the Coulomb interaction arising from the in-plane polarizability and finite width of few-layer materials^61–63^. We take the phenomenological form

2 $$V(q)\propto \frac{1}{q(1+{l}_{{{{\rm{RK}}}}}q)}\tanh \frac{q\xi }{2},$$

where *l*_RK_ is the RK length scale. Increasing *l*_RK_ softens the short-distance repulsion and reduces *V*_0_/*V*_1_, while somewhat enhacing *V*_*m*_/*V*_1_ for *m* > 1 (see Fig. 2a, green line). Hence, less additional suppression of *V*_0_ is needed to obtain the FTI, as shown in Fig. 2c. The FTI phase roughly follows a locus of constant *V*_0_/*V*_1_. Relaxing the FTI condition to only require *Δ*_9_ > 0 (i.e. the nine lowest states lie in the correct momentum sectors) does not significantly enlarge the stability region (see Fig. S10 in Supplementary Note 1 d).

Finally, we also consider higher Landau levels (LLs) as potential hosts for FTIs. As discussed in Supplementary Note 1 E, while the non-relativistic *n* = 1 LL does not stabilize an FTI for the parameters considered, we find that the Dirac fermion *n* = 1 LL yields an FTI for a significantly smaller suppression of the *V*_0_ pseudopotential compared to the LLL case (*n* = 0). The reason is that for both types of *n* = 1 LLs, the different form factors of the wavefunctions lead to Eq. (2) having a smaller *V*_0_/*V*_1_ ratio, compared to the LLL case. However, the higher *V*_*m*_/*V*_1_ ratios are sufficiently enhanced for the non-relativistic *n* = 1 LL such that the FTI does not survive for valley-isotropic interactions.

### FTI in twisted MoTe_2_

We now turn to modeling *t*MoTe_2_ at filling *ν* = − 4/3, focusing on *θ* = 3.7° about which FCIs^7–10^ were observed (at fillings  < 1). Unless otherwise stated, we use the single-particle first-harmonic continuum model^64^ with parameters m_*_ = 0.6*m*_*e*_, *w* = − 18.8meV, *V* = 16.5meV and *ψ* = − 105.9° taken from ref. ^22^. Higher harmonics are only necessary for describing substantially smaller twist angles. Due to spin-orbit coupling, the moiré bands are spin-valley locked.

**Fig. 4:**
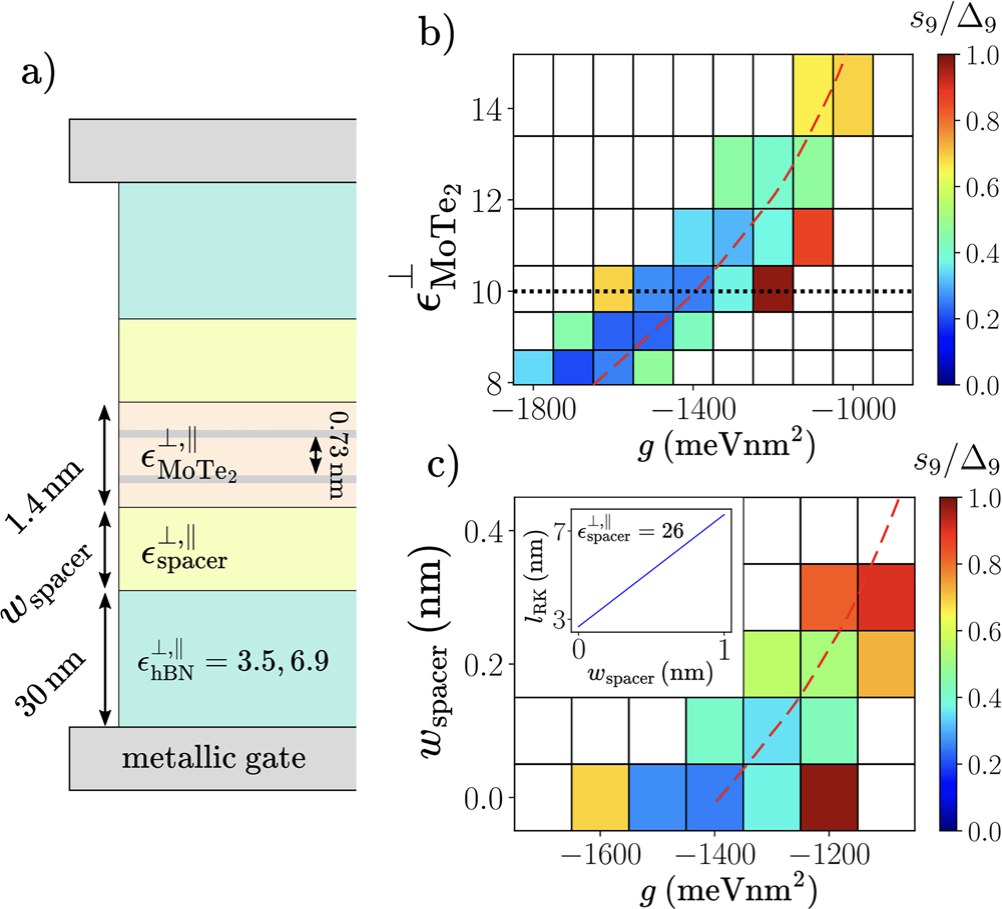
Dependence on the dielectric environment. One-band-per-valley (1BPV) calculations are performed at *θ* = 3. 7^∘^ for a tilted *N*_*s*_ = 15 lattice (see Fig. 1b inset). **a** Schematic cross-section of a *t*MoTe_2_ device, where we assume mirror symmetry in the vertical direction. The continuum model degrees of freedom are positioned at the Mo planes (gray lines) at *z* = ± 0.365 nm, and embedded in the dielectric environment generated by the device stack. Optional spacer dielectrics (yellow) can be inserted between *t*MoTe_2_ and the encapsulating hBN substrates. We fix ${w}_{{{{\rm{hBN}}}}}=30\,\,{{{\rm{nm}}}},{w}_{{{{{\rm{MoTe}}}}}_{2}}=1.4\,\,{{{\rm{nm}}}},{d}_{{{{{\rm{MoTe}}}}}_{2}}=0.73\,\,{{{\rm{nm}}}}$, ${\epsilon }_{\,{{{\rm{hBN}}}}}^{\perp }=3.5$ and ${\epsilon }_{\,{{{\rm{hBN}}}}}^{\parallel }=6.9$. **b** *s*_9_/*Δ*_9_ (color) as a function of ${\epsilon }_{{{{{\rm{MoTe}}}}}_{2}}^{\perp }$ and *g* for fixed ${\epsilon }_{{{{{\rm{MoTe}}}}}_{2}}^{\parallel }/{\epsilon }_{{{{{\rm{MoTe}}}}}_{2}}^{\perp }=2.1$ in the absence of spacers (*w*_spacer_ = 0). Dotted line indicates ${\epsilon }_{{{{{\rm{MoTe}}}}}_{2}}^{\perp }$ used in Fig. 3. **c ***s*_9_/*Δ*_9_ (color) as a function of *w*_spacer_ and *g* for fixed ${\epsilon }_{{{{{\rm{MoTe}}}}}_{2}}^{\perp }=10$, ${\epsilon }_{{{{{\rm{MoTe}}}}}_{2}}^{\parallel }=21$ and ${\epsilon }_{\,{{{\rm{spacer}}}}}^{\perp ,\parallel }=26$. Inset shows the effective *q* → 0 RK parameter *l*_RK_ for the intralayer interaction as a function of *w*_spacer_, which is fitted well by *l*_RK_ = 5.07 *w*_spacer_ + 2.67 nm. Red dashed lines in **b**,**c**) indicate constant *V*_0_/*V*_1_ = 1.11 for the intralayer interaction.

To capture the screening effects due to the *t*MoTe_2_ thickness and the dielectric environment, we compute the interaction potential by solving the Poisson equation in a typical dual-gated hBN-encapsulated geometry (see Fig. 4a without the spacer slabs). As detailed in Supplementary Note 4, our treatment of the interaction potential ${V}_{ll^{\prime} }(q)$ accounts for the anisotropic dielectric screening from hBN and MoTe_2_ slabs of widths *w*_hBN_ and ${w}_{{{{{\rm{MoTe}}}}}_{2}}$, and the dependence on the layer indices $l,l^{\prime}$ coming from the finite interlayer distance ${d}_{{{{{\rm{MoTe}}}}}_{2}}=0.73\,\,{{{\rm{nm}}}}$. Unless stated otherwise, we take ${\epsilon }_{{{{{\rm{MoTe}}}}}_{2}}^{\perp }=10$ and ${\epsilon }_{{{{{\rm{MoTe}}}}}_{2}}^{\parallel }=21$ from first principles calculations on bilayer MoTe_2_^65^. At long wavelengths, the inclusion of the MoTe_2_ screening results in an effective *l*_RK_ ≃ 2.7 nm for the $l=l^{\prime}$ interaction (Fig. 4c inset). Anticipating from our LL results that a suppression of the short-range repulsion will be necessary to stabilize FTIs, we add an additional onsite intralayer interaction

3 $$\delta {V}_{ll^{\prime} }(q)=g{\delta }_{ll^{\prime} }.$$

Due to fermion antisymmetry, Eq. (3) is ineffective between particles in the same valley.

The ED calculations are performed on torus geometries with *N*_*s*_ moiré unit cells. In order to access different *N*_*s*_ while maintaining an aspect ratio close to 1, we frequently utilize tilted boundary conditions^41,66^ (see Fig. 1b inset for mBZ momentum grid for *N*_*s*_ = 15). For large system sizes, computational complexity necessitates projection to the highest valence band (labeled band 0 in Fig. 1a) in each valley *η* = ± , i.e., a one-band-per-valley (1BPV) calculation. However, as emphasized in ref. ^19^, band mixing is important owing to strong interactions and the relatively small gap  ≃ 10 meV to the next valence band. To reduce the computational cost, we adopt a truncated Hilbert space approach^67^ in some computations, such that we also allow a maximum number ${N}_{\max }^{1}$ of holes in the next valence band (band 1 in Fig. 1a). Configurations with a large number of holes in band 1 are not expected to be important for the low-energy physics due to the kinetic energy cost. ${N}_{\max }^{1}={N}_{s}$ corresponds to a two-bands-per-valley (2BPV) calculation.

Figure 3a shows a representative 1BPV (${N}_{\max }^{1}=0$) many-body spectrum in the FTI phase. Apart from the small *s*_9_/*Δ*_9_, spectral evolution under flux threading provides additional evidence for an FTI (see Fig. 1b). We emphasize that these results are acquired when the long-range part of the interaction is valley-isotropic, which is the physically natural regime since the valleys are related by TR symmetry and hence the corresponding Bloch states occupy the same region in real space. We find that, similar to the LLL model, an attractive *g* < 0 is necessary to stabilize an FTI. Additional results, including dependence of FTI stability on valley-anisotropic interactions and displacement field, are provided in Supplementary Note 5.

Figure 3b illustrates that *s*_9_/*Δ*_9_ < 1 over a range of finite *g* for *N*_*s*_ = 15 and ${N}_{\max }^{1}=0$. To connect more quantitatively with the LLL limit, we use the geometric relation^68^ for the effective magnetic length ${\ell }_{B}^{* }=\scriptstyle\sqrt{\frac{{A}_{M}}{2\pi }}=2.02\,\,{{{\rm{nm}}}}$ for *θ* = 3.7°, where *A*_*M*_ is the area of the unit cell in *t*MoTe_2_. This allows for evaluation of the pseudopotentials *V*_*m*_ for the intralayer interaction potential, revealing that these FTI states lie in the range 1.03 < *V*_0_/*V*_1_ < 1.18. This lends support to the notion that, akin to the LLL model, the ratio *V*_0_/*V*_1_ is a key indicator for whether an interaction potential admits FTI states. We note that for the potential ${V}_{ll^{\prime} }(q)\propto \frac{\tanh \frac{q\xi }{2}}{q}$ obtained by setting ${w}_{{{{{\rm{MoTe}}}}}_{2}}={d}_{{{{{\rm{MoTe}}}}}_{2}}=0$ and ignoring the hBN anisotropy, we do not find FTI states for any *g*, while introduction of a finite *l*_RK_, as in Eq. (2), yields FTIs (see Figs. S22 and S25 in Supplementary Note 5 A). This implies that the higher pseudopotentials still play a quantitatively significant role, and underscores the importance of detailed analysis of the dielectric screening environment.

Figure 3b also shows the effects of increasing the system size (*N*_*s*_ = 18) and band mixing (${N}_{\max }^{1}=1$). In both cases, we observe a drift of the FTI region towards smaller values of ∣*g*∣, meaning towards the realistic situation. However, current finite-size restrictions prevent an extrapolation to the thermodynamic limit with full band mixing. We have checked that the ground state lies in *S*_*z*_ = 0 for the parameters shown in Fig. 3b. The system is fully spin-polarized for *g* = 0 in 1BPV calculations, and previous work has shown that the ground state in 2BPV calculations is not fully polarized^19^. Here, the short-range attraction *g* < 0 acts to decrease the repulsive energy between opposite valleys, allowing the possibility for full demagnetization in 1BPV calculations. Increasing ${N}_{\max }^{1}$ also lowers the energy of the *S*_*z*_ = 0 sector relative to the magnetized sectors^19^ (see Figs. S32 and S33 in Supplementary Note 5 B), ensuring that the FTI phase lies within the non-magnetic regime at *ν* = − 4/3.

In Fig. 3c, we fix *g* = − 1400 meVnm^2^ and repeat the calculations for a range of continuum model parameters *ψ*, *w* and *V*. The FTI phase roughly tracks where the Berry curvature fluctuations of the lowest valence band are smallest (dashed line), suggesting that the FTI benefits from uniform Berry curvature^69^. For smaller ∣*w*/*V*∣, the FTI can survive to a significantly lower ∣*g*∣ (see Fig. S38 in Supplementary Note 5 C). In Fig. 3d, still keeping *g* fixed, we instead vary *θ* and introduce an artificial factor *κ*_SP_ that multiplies the kinetic term. As the twist angle is reduced, the FTI phase attains a lower *s*_9_/*Δ*_9_ and is realized at larger *κ*_SP_, including the physical value *κ*_SP_ = 1. The FTI phase roughly tracks where the Hartree renormalized bandwidth is minimal (see Fig. S35 in Supplementary Note 5 C). We caution that the actual continuum model parameters, as fitted to ab initio data, are expected to vary with *θ*^24^, but not considerably over the range shown in Fig. 3. We note that 1BPV calculations projected only into band 1 can stabilize FTIs for significantly lower values of ∣*g*∣ (see Fig. S39 in Supplementary Note 5 C). This suggests that FTIs may potentially be obtained also at higher hole filling factors such as *ν* = − 10/3 by fully hole-occupying band 0 and partially hole-occupying band 1.

### Dielectric screening

Changes to the dielectric environment can affect the functional form of the interaction potential and help stabilize an FTI for smaller ∣*g*∣. The 1BPV calculations of Fig. 4b demonstrate that the required values of ∣*g*∣ reduce as the relative permittivity of MoTe_2_ increases. An enhanced ${\epsilon }_{{{{{\rm{MoTe}}}}}_{2}}^{\perp ,\parallel }$, which could arise due to screening from remote moiré bands, acts to confine the electric field lines within the *t*MoTe_2_ system, which relatively suppresses the short-distance repulsion. Note that weakening the interaction too much eventually destabilizes the FTI. Another way to selectively weaken the short-range repulsion is to surround *t*MoTe_2_ with thin spacers with moderately high dielectric constant (Fig. 4a), which works to impede the electric field lines from spreading into the encapsulating hBN. For example with ${\epsilon }_{\,{{{\rm{spacer}}}}}^{\perp ,\parallel }=26$ appropriate for HfO_2_^70^, increasing *w*_spacer_ (Fig. 4c) leads to a drift of the FTI region to smaller ∣*g*∣, approximately following a stripe of constant *V*_0_/*V*_1_. The inset shows that the spacer increases the effective RK length. Our current calculations find that the spacer needs to be quite narrow  ≲ 0.4 nm to preserve the FTI, since the spacer also increases the FTI spacing/gap ratio. Note that if *ϵ*_spacer_ is too large, then *V*_0_/*V*_1_ is enhanced because the spacer effectively acts as a metallic gate for *ϵ*_spacer_ → *∞*. *V*_0_/*V*_1_ can also be substantially reduced with small but unphysical *ϵ*_spacer_ ≪ 1, which confines the field lines to the *t*MoTe_2_ (see Fig. S20 in Supplementary Note 4 B).

## Discussion

In our investigation of FTIs we use realistic *t*MoTe_2_ parameters, with the only extra ingredient invoked being a short-range attraction *g*. A possible origin for this is electron-phonon coupling, which has been argued to influence the balance between competing phases in moiré graphene^71–73^. In Supplementary Note 3, we consider the monolayer phonons, yielding a contribution *g* ≃ − 20 meVnm^2^ which is significantly smaller than the scale *g* ~ − 1000 meVnm^2^ that is needed for the FTI in our calculations. While the precise size of this discrepancy can be affected by other sources of short-range interaction, we discuss the possibility that our current finite-size ED studies overestimate the required strength of short-range attraction. First, our calculations of the FTI in both *t*MoTe_2_ and the LLL model exhibit appreciable drifts towards weaker ∣*g*∣ for larger system sizes. The former also displays a similar drift with respect to band mixing (Fig. 3b). It would be interesting to investigate other methods of incorporating additional bands, such as those leveraged to treat Landau level mixing in the FQH setting^67,74–78^. Second, the stability of the FTI varies with twist angle *θ* and the band structure parameters (Fig. 3c, d), and our extensive calculations have so far neither optimized these choices, nor considered other filling factors in detail. We note that the continuum model parameters change significantly as a function of *θ*^24^ and for different TMDs. Theoretical work has indicated that the second valence band of low-angle *θ* ≈ 2.1° *t*MoTe_2_ exhibits similarities with the non-relativistic *n* = 1 LL^79–81^. Our calculations show that this LL does not yield an FTI, which suggests that an FTI in *θ* ≈ 2.1° *t*MoTe_2_ at *ν* = − 8/3 or  − 10/3 is unlikely, at least in the absence of band-mixing. However, we find that resemblance to the Dirac *n* = 1 LL may support an FTI with a smaller ∣*g*∣ compared to the LLL. Third, the details of dielectric screening play an important role in shaping the interaction potential, as evidenced in the dependence of the FTI phase on ${\epsilon }_{{{{{\rm{MoTe}}}}}_{2}}$ (Fig. 4b). As we have briefly discussed, dielectric engineering can help reduce the required ∣*g*∣ (Fig. 4c), an approach that should be explored in future work. Therefore, we believe that the *t*MoTe_2_ material platform warrants further attention to determine whether the experimental realization of an FTI is feasible here. We note that our calculations are unbiased, and we consider a large number of realistic effects in TMDs. The fractionalized helical edge modes of the FTI, which could be locally imaged^82^, are stable against *S*_*z*_-preserving perturbations, and would yield quantized signatures in (non-local) transport measurements (see Supplementary Note 6).

Given the similarities between the results for the FTI in *θ* ≈ 3.7° *t*MoTe_2_ and the LL models, we believe our work has ramifications for obtaining FTIs in other systems. In particular, we expect the pseudopotential ratio *V*_0_/*V*_1_ will still play a crucial role in stabilizing FTI phases. The construction of appropriate trial wavefunctions will likely shed further light on the optimal *V*_0_/*V*_1_ window. We believe density matrix renormalization group studies that can analyze the drift of ∣*g*∣ can reveal whether, as we speculate, the FTI could appear under real-world sample conditions.

*Note added.—* During the preparation of this manuscript, an experimental report^40,83^ of a state with a *R*_*xx*_ plateau but without a *R*_*xy*_ plateau in *θ* ≈ 2.1° *t*MoTe_2_ at *ν* = − 3 appeared; it was suggested, but not yet confirmed, that this could be evidence for the FQSHE. This was followed by several theoretical works^84–90^ discussing possible related aspects. We note that no prior theory has directly addressed the feasibility of FTIs or FQSH states in *t*MoTe_2_ using microscopic unbiased calculations. A calculation or demonstration of a fractionalized state at *θ* ≈ 2.1° *t*MoTe_2_ at *ν* = − 3 is still missing, and must be able to explain the continuous *R*_*xy*_ dependence on density.

## Supplementary information

Supplementary material

## Data Availability

The data that support the findings of this study are available from the corresponding author upon reasonable request.

## Code availability

The code that was used to produce the data in this study is available from the open source package DiagHam.
