# Supplementary material for: Regarding the existence of abelian fractional topological insulators in twisted MoTe2 and related systems
Source: Commun Phys. 2026 Jan 16;9(1):52. doi: 10.1038/s42005-025-02483-6 (PMC12893902; doi:10.1038/s42005-025-02483-6)
Supplement: Supplementary file 1 — Supplementary material [file 42005_2025_2483_MOESM1_ESM.pdf]

— Supplementary material for "When Could Abelian Fractional Topological Insulators Exist in Twisted $MoTe_2$ (and Other Systems)" —

CONTENTS

1. Opposite-fields Lowest Landau level model 2
   A. Model and methods 2
   B. $V_0^{+-} - V_1^{++} - V_1^{+-}$ phase diagram 3
   C. Gate-screened Coulomb interactions 5
   D. Rytova-Keldysh correction 8
   E. Other Landau levels 10

2. Review of $t\mathrm{MoTe}_2$ model 13
   A. Single-particle continuum model 13
   B. Interactions 14

3. Contribution to short-range interaction from electron-phonon coupling in $t\mathrm{MoTe}_2$ 15

4. Dielectric screening of interaction potential 18
   A. General setup 18
      a. Isotropic material embedded in isotropic dielectric substrate 20
      b. Anisotropic material embedded in anisotropic dielectric substrate 20
   B. Numerical results for V_0/V_1 21

5. Additional numerical data for $t\mathrm{MoTe}_2$ 25
   A. Effects of system parameters 25
   B. Spin depolarization 26
   C. Insights from Hartree-Fock 26
   D. Fractional Chern insulators at $\nu = -2/3$ 27

6. Edge mode stability and transport 36
   A. Stability analysis 36
   B. Landauer-Büttiker analysis 37

Supplementary Note 1: Opposite-fields Lowest Landau level model

In this section, we describe in detail a set of ED calculations within a toy model which comprises a pair of lowest Landau levels (LLLs) where the two valleys $\eta = \pm$ experience magnetic fields oriented along opposite directions. Note that we can use the terms 'spin' and 'valley' interchangeably, since they are locked together in the tMoTe$_2$ context owing to strong spin-orbit coupling. In Supplementary Note 1 A, we describe the model and details of the ED implementation. In Supplementary Note 1 B, we study the phase diagram within a restricted space of Haldane pseudopotentials $V_m^{\eta\eta'}$ parameterizing the interaction. In Supplementary Note 1 C and 1 D, we consider more realistic interactions corresponding to variations of the gate-screened Coulomb interaction. In Supplementary Note 1 E, we discuss how the physics changes for other Landau levels beyond the LLL.

A. Model and methods

Consider the single-particle Hamiltonian of electrons of charge $-e$ confined to a 2D plane and minimally coupled to a valley-dependent vector potential $\mathbf{A}^\eta(\mathbf{r})$

$$\hat{H}_0^\eta = \frac{(\mathbf{p} + e\mathbf{A}^\eta(\mathbf{r}))^2}{2m}, \tag{E1}$$

where $\nabla \times \mathbf{A}^\eta = \eta(0,0,B)^T$, B is the magnetic field, and $\eta = +\,(-)$ labels electrons in valley $+\,(-)$. $\mathbf{p}$ and $\mathbf{r}$ are the 2D momentum and position operators, respectively. We define the magnetic length $\ell_B = \frac{\hbar}{eB}$ which will often be set to unity. The system preserves time-reversal symmetry $\mathcal{T}$ since the magnetic fields in the two valley sectors are equal and opposite, and the corresponding LLs have opposite Chern numbers $C^\eta = \eta$. Furthermore, the Hamiltonian has a $U_\mathrm{v}(1)$ valley conservation symmetry whose generator is $2\hat{S}_z = \hat{N}_+ - \hat{N}_-$, where $\hat{N}_\eta$ is the number operator in valley η. We construct the single-particle Hilbert space by projecting to the lowest Landau levels (LLLs) in the two valley sectors. We refer to this system simply as the LLL model, with the understanding that it always corresponds to the opposite magnetic fields setup described above. Similar setups have been considered previously in [44–58].

To incorporate interactions into the LLL model, we consider $\mathcal{T}$- and S_z-preserving valley-dependent interactions of the form (in the continuum)

$$\hat{H}_\mathrm{int} = \frac{1}{2}\sum_{\eta\eta'}\int \frac{d^2\mathbf{q}}{(2\pi)^2} V^{\eta\eta'}(q)\rho^\eta_\mathrm{LLL}(\mathbf{q})\rho^{\eta'}_\mathrm{LLL}(-\mathbf{q}) \tag{E2}$$

where $\rho^\eta_\mathrm{LLL}(\mathbf{q})$ is the LLL-projected density operator. While the bare interaction potential $V^{\eta\eta'}(q)$ is isotropic in real 2D space, we allow for anistropies in valley space. The symmetries require

$$V^{++}(q) = V^{--}(q), \quad V^{+-}(q) = V^{-+}(q) \tag{E3}$$

such that we will often only refer to the $++$ (intravalley) and $+-$ (intervalley) components explicitly. In certain situations, including for some of the tMoTe$_2$ calculations in this work, we will consider uniformly scaling the intervalley interaction relative to the intravalley interaction

$$V^{+-}(q) = \lambda V^{++}(q) \tag{E4}$$

where λ is the valley anistropy parameter and $\lambda = 1$ its physical value.

In the LLL model, it is convenient to parameterize the interaction potential in terms of Haldane pseudopotentials

$$V_m^{\eta\eta'} \equiv \int \frac{d^2\mathbf{q}}{(2\pi)^2} V^{\eta\eta'}(q) L_m(q^2\ell_B^2) e^{-q^2\ell_B^2} \tag{E5}$$

where $L_m(x)$ is the Laguerre polynomial. $V_m^{\eta\eta'}$ characterizes the interaction energy of two particles with relative angular momentum m. Note that $V_m^{\eta\eta}$ for m even does not affect the physics due to fermionic statistics.

We perform ED calculations on a torus with magnetic periodic boundary conditions, and restrict to square geometries with aspect ratio of 1. In terms of the number of flux quanta N_Φ and particle numbers N_+, N_-, the filling factors are defined by $\nu_+ = N_+/N_\Phi$ and $\nu_- = N_-/N_\phi$. The Hamiltonian has a particle-hole symmetry (PHS) that relates $(\nu_+, \nu_-) \leftrightarrow (1-\nu_+, 1-\nu_-)$. We will mostly work at filling $\nu_+ = \nu_- = 1/3$, but owing to PHS, this is analogous to the $\nu = -2/3 - 2/3$ situation appropriate for tMoTe$_2$ (if band mixing is neglected). To reduce the computational

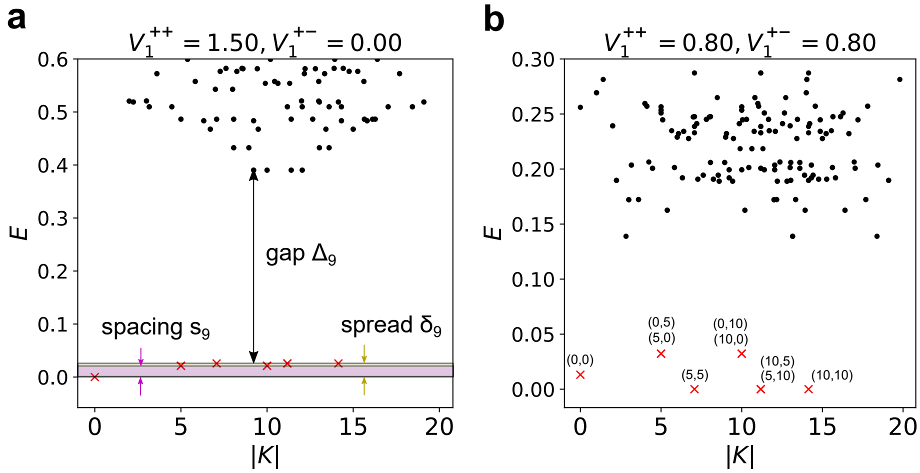

FIG. S1. **Exact diagonalization spectra for FTI phases in the LLL model at $\nu_+ = \nu_- = 1/3$ for selected V_1^{++}/V_0^{+-} and V_1^{+-}/V_0^{+-}.** $|K| = \sqrt{K_x^2 + K_y^2}$ denotes the magnitude of the many-body momentum. The lowest 4 states (lowest state) are kept for momentum sectors that contain (do not contain) a state in the FTI ground state manifold. The 9 states in the FTI ground state manifold are denoted with red crosses, and their momenta (K_x, K_y) are labelled for b). Note that some ground states are overlapping in the figures, e.g. those with momenta $(K_x, K_y) = (0, 5)$ and $(5, 0)$. a) illustrates how the FTI spacing s_9, spread δ_9 and gap Δ_9 are defined. $V_0^{+-} = 1$ for both plots. In this case, s_9 is only slightly smaller than δ_9. Square torus geometry with $N_\Phi = 15$ flux quanta.

cost, we exploit the many-body translation symmetries [59, 92] and diagonalize within many-body momentum sectors labelled by $\mathbf{K} = (K_x, K_y)$, where K_x and K_y take values $0, \ldots, N_\Phi - 1$.

To diagnose the presence of FTIs in the finite-size numerics, we consider properties of the many-body spectrum for $S_z = 0$. Focusing on the $\nu_+ = \nu_- = 1/3$ FTI, we first identify the momenta corresponding to the 9 (nearly)-degenerate FTI ground states. These can be determined by considering the limit of two decoupled $\nu_\eta = 1/3$ FQH Laughlin states in the two valley sectors. For example for $N_\Phi = 15$, this yields a single state for each of $(K_x, K_y) = (5i_x, 5i_y)$ with $i_x, i_y = 0, 1, 2$. We define the FTI spread δ_9 as the energy bandwidth of the 9 lowest states with these quantum numbers, i.e. we postulate that these 9 states form the FTI ground state manifold. We define the FTI spacing s_9 as the maximum energy difference between adjacent levels in the FTI manifold, irrespective of momentum. The FTI gap Δ_9 is defined as the energy of the lowest state not in this manifold minus the energy of the highest energy state in this manifold. Note that the gap Δ_9 defined this way can be negative, which certainly rules out the possibility of an FTI phase. Figure S1a illustrates these definitions for a system deep in the FTI regime. The condition that the spread is less than the gap (i.e. $\delta_9 < \Delta_9$ for our problem) is commonly used as a condition for the identification of the topological phase in the FCI/FTI literature. In this work, we take the less stringent condition of the spacing/gap ratio s_9/Δ_9 being less than 1 as a necessary condition for determining an FTI phase. The rationale behind this criterion is that to visually separating the 9 low energy states from the higher energy states requires $s_9 < \Delta_9$. The presence of a path in parameter space where the spacing/gap ratio remains small and connects to the limit of decoupled FQH states demonstrates adiabatic continuity and provides further evidence of an FTI phase. By considering other S_z sectors, we can also determine the presence of a positive valley gap.

B. $V_0^{+-} - V_1^{++} - V_1^{+-}$ phase diagram

A model $\nu_+ = \nu_- = 1/3$ FTI state can be trivially obtained by including only a single non-vanishing pseudopotential V_1^{++} (recall from Eq. E3 that $V_1^{--} = V_1^{++}$ due to the valley symmetries). This corresponds to exact $\nu = 1/3$ Laughlin states in each valley sector, which are decoupled due to the absence of intervalley interactions. The stability of the FTI against including intervalley interactions was partially addressed by Ref. 44, which carried out ED calculations for the LLL model on the torus for $N_\Phi = 15$. In particular, they investigated the phase diagram in the $S_z = 0$ sector as a function of the ratio V_0^{+-}/V_1^{++}, with all other pseudopotentials, including V_1^{+-}, set to zero. For large and repulsive V_0^{+-}/V_1^{++}, Ref. 44 found that the system was gapless and phase separated into regions of opposite valley polarization. For $V_0^{+-}/V_1^{++} = 1$, Ref. 44 found that the system remained in the FTI phase based on computing wavefunction overlaps with the model wavefunction corresponding to decoupled Laughlin states. However, we emphasize that $V_0^{+-}/V_1^{++} = 1$ does not correspond to a valley-isotropic interaction, since the intervalley interaction is ultra short-

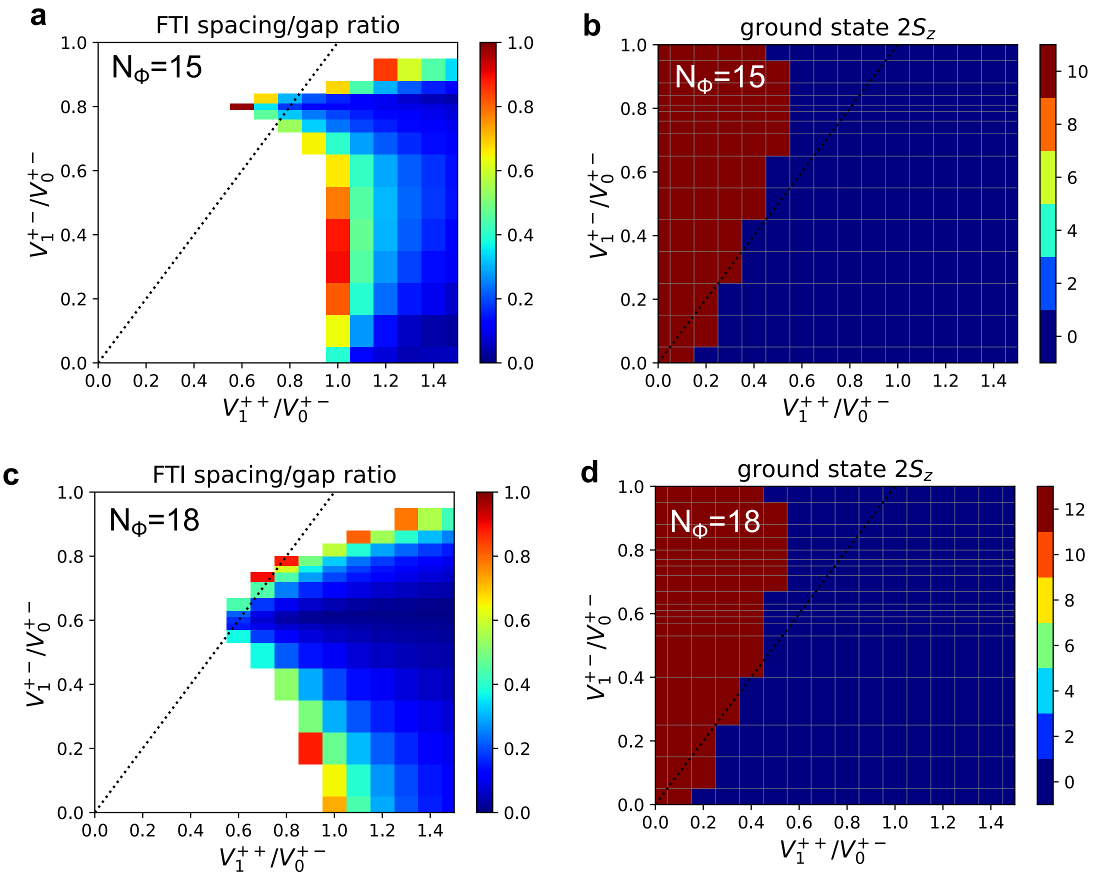

FIG. S2. **FTI spacing/gap ratio and ground state valley polarization as a function of pseudopotentials V_1^{++}/V_0^{+-} and V_1^{+-}/V_0^{+-} in the LLL model at $\nu_+ = \nu_- = 1/3$.** All other pseudopotentials are set to zero. a) See text for definitions of the spacing s_9 and gap Δ_9. White regions correspond to where the spacing is greater than the gap, or the gap is negative. Dotted line indicates isotropic interactions $V_1^{++} = V_1^{+-}$. $N_\Phi = 15$ flux quanta. b) Valley polarization $2S_z = N_+ - N_-$ of the ground state across all valley sectors for the same grid of pseudopotentials as in a). c,d) Same as a,b) except for $N_\Phi = 18$ flux quanta. All plots use square torus geometry.

range $\sim \delta(\mathbf{r})$, while the intravalley interaction has a longer range $\sim \nabla^2\delta(\mathbf{r})$. In contrast, the interaction potential relevant for moiré materials such as tMoTe$_2$ is expected to be isotropic in valley space. To the best of our knowledge, there are no prior theoretical studies that have demonstrated an FTI phase for valley-isotropic interactions in a fermionic system, whether for LLs or lattice systems.

In order to address this gap, we first consider an expanded parameter space consisting of pseudopotentials $V_0^{+-}, V_1^{++}, V_1^{+-}$ in the LLL model. Without loss of generality, we set $V_0^{+-} = 1$. As mentioned above, V_0^{++} does not need to be considered since only the odd pseudopotentials are relevant for the intravalley interaction owing to fermionic statistics. Crucially, we include parameter sets which lie along the valley-isotropic line $V_1^{++} = V_1^{+-}$. The results for the FTI spacing/gap ratio for $N_\Phi = 15$ and $S_z = 0$ are presented in Fig. S2a, where parameters that yield ground states that are consistent with our criteria for an FTI phase (see Sec. 1 A) are colored. $V_1^{++} \to \infty$ on the horizontal axis corresponds to the idealized limit of decoupled FQH states and vanishing spacing/gap ratio. In agreement with Ref. 44, we find that the FTI survives to $V_1^{++} = V_0^{+-}$, though it does not persist for much larger V_0^{+-}. It is clear that decreasing V_1^{++} generally is deleterious for the FTI.

Curiously, we find non-monotonic behavior of the spacing/gap ratio as a function of V_1^{+-} in the FTI region at $V_1^{++} \simeq V_0^{+-}$. This suggests that a finite V_1^{+-} can actually be beneficial for the FTI phase, which runs counter to the expectation that intervalley interactions are harmful to the FTI. Strikingly, we observe a small window where an FTI is stabilized with valley-isotropic interactions (see dotted line in Fig. S2a). We find that this occurs for $V_1^{++} = V_1^{+-} \simeq 0.8V_0^{+-}$, and the spacing/gap ratio remains small along a parameter path connected to the ideal decoupled FQH limit. The many-body spectrum is plotted in Fig. S1b, and exhibits a spacing/gap ratio $\simeq 0.3$.

Figure S2b shows the valley polarization S_z of the ground state across all valley sectors (note that the maximum $S_z = N_+ - N_- = \frac{2N_\Phi}{3}$ for $\nu_+ + \nu_- = \frac{2}{3}$). For small V_1^{++}, the ground state lies in the fully magnetized sector. However,

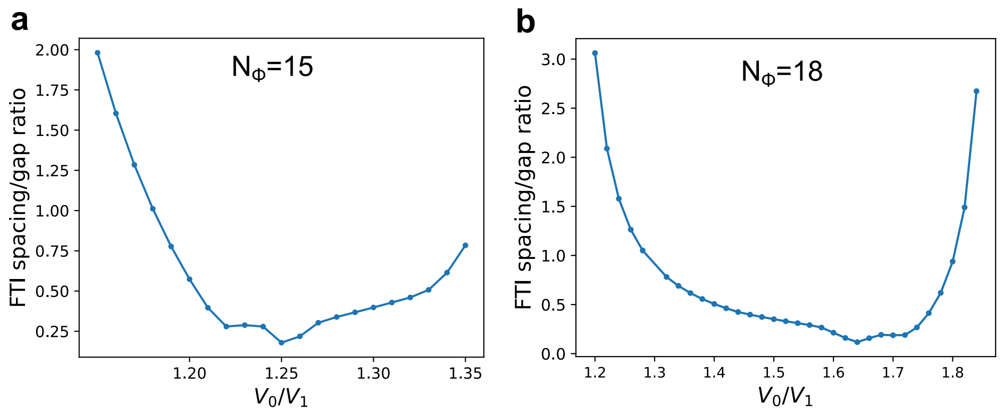

FIG. S3. **FTI spacing/gap ratio as a function of valley-isotropic pseudopotentials $V_0^{\eta\eta'} = V_0$ and $V_1^{\eta\eta'} = V_1$ in the LLL model at $\nu_+ = \nu_- = 1/3$.** All other pseudopotentials are set to zero. Square torus geometry with a) $N_\Phi = 15$, and b) 18 flux quanta.

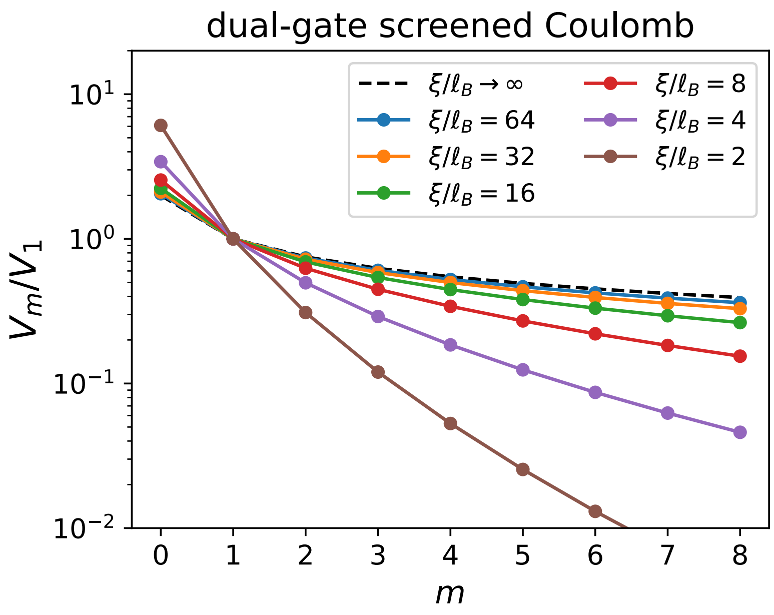

FIG. S4. **LLL pseudopotentials V_m of the dual-gate screened Coulomb interaction (Eq. E6) for different screening lengths ξ/ℓ_B.** Dashed line corresponds to the unscreened Coulomb limit.

for the parameters where the lowest states in the $S_z = 0$ sector form an FTI, we find that the ground state of the system is indeed non-magnetized.

Figure S2c shows analogous results for the $S_z = 0$ phase diagram for $N_\Phi = 18$. Compared to the $N_\Phi = 15$ calculations, the window of FTI stability along the isotropic line is wider, and shifts to higher V_0/V_1. As demonstrated in Fig. S2d, the ground state of the system is non-magnetized for parameters where we find an FTI.

In Fig. S3, we show additional results at $S_z = 0$ along the valley-isotropic line $V_1^{\eta\eta'} = V_1$ for $N_\Phi = 15, 18$ and $S_z = 0$. For the larger system size, the FTI stability window increases, and the minimum spacing/gap ratio decreases.

C. Gate-screened Coulomb interactions

Having demonstrated the existence of FTIs for valley-isotropic interactions within a restricted set of allowed pseudopotentials V_0, V_1, we now turn to more realistic interactions that are relevant for moiré systems. We consider the

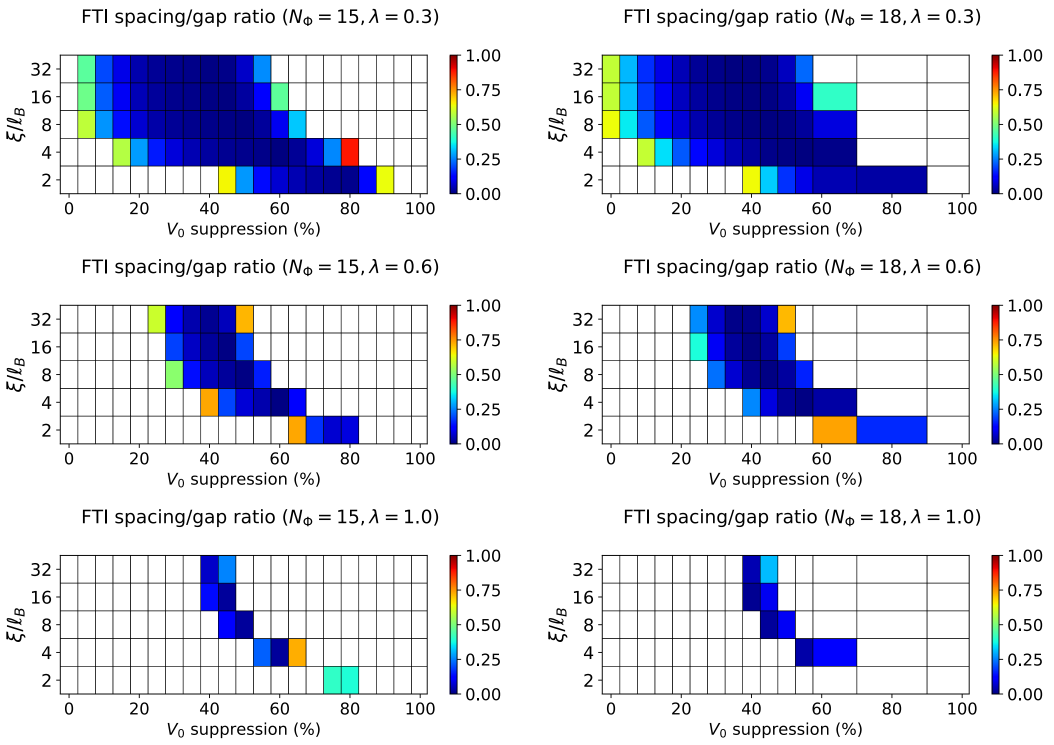

FIG. S5. **FTI spacing/gap ratio as a function of V_0 suppression and dual-gate Coulomb screening length ξ/ℓ_B for different interaction anisotropy parameters λ in the LLL model at $\nu_+ = \nu_- = 1/3$.** White regions correspond to where the spacing is greater than the gap, or the gap is negative. Square torus geometry with $N_\Phi = 15, 18$ flux quanta.

Coulomb potential screened by two metallic gates with separation ξ, and positioned symmetrically on either side of the 2D system

$$V^{\eta\eta'}(q) = V_\xi(q) = \frac{e^2}{2\epsilon_0\epsilon q}\tanh\frac{q\xi}{2}, \tag{E6}$$

where ϵ is the relative permittivity (whose value is irrelevant for the LLL model which lacks band dispersion). The corresponding pseudopotentials are plotted in Fig. S4. In the absence of screening ($\xi/\ell_B \to \infty$), the Coulomb pseudopotentials $V_m \sim \binom{2m}{m}4^{-m}$ decay algebraically $\sim m^{-1/2}$ at large m. For finite ξ, they decay exponentially with sufficiently large m. The limit of small ξ/ℓ_B is well approximated by the $(V_0^{+-}, V_1^{++}, V_1^{+-})$ setup in Sec. 1 B with $V_1^{++} = V_1^{+-}$ and large V_0^{+-}/V_1^{++}.

Reference 45 has previously performed ED numerics on the LLL model on the sphere using the unscreened ($\xi/\ell_B \to \infty$) limit of Eq. E6, and found a compressible state at $\nu_+ = \nu_- = 1/3$ that is likely disordered. For the opposite limit $\xi/\ell_B \to 0$, the system is phase-separated according to the discussion of Sec. 1 B since V_0/V_1 is large. Therefore, to expand the space of parameters and increase the chances of finding a stable FTI phase, we introduce an additional onsite attraction $\propto -\delta(\mathbf{r})$, which is equivalent to subtracting a constant from $V_\xi(q)$ in Eq. E6, and only affects the $m = 0$ pseudopotential V_0. In the following calculations, we parameterize the strength of the onsite attraction by the resulting percentage suppression of V_0 compared to using only the screened Coulomb interaction.

Figure S5 shows the FTI spacing/gap ratio for $N_\Phi = 15, 18$ as a function of the screening length ξ/ℓ_B and the suppression of V_0, for different values of the valley anisotropy λ [Eq. (E4)]. For $\lambda = 0$ (not shown) corresponding to decoupled valley sectors, the gap is positive and the spacing is exactly zero (due to center-of-mass degeneracy) for all ξ and V_0. For small $\lambda = 0.3$, the region of FTI stability persists for a broad range of V_0 suppression. This shrinks significantly as λ is increased, but even for valley-isotropic interactions ($\lambda = 1$), we find that the FTI phase survives in a sliver of the phase diagram. We observe a slight drift towards less V_0 suppression for the FTI phase as N_Φ increases.

Figure S6 examines the region of FTI stability for $\lambda = 1$ and $N_\Phi = 15$ in more detail. The required suppression of V_0 decreases for larger ξ, mainly because the original V_0/V_1 for the gate-screened interaction becomes smaller as the gates are separated from each other (Fig. S4), and tends to $V_0/V_1 = 2$ in the unscreened Coulomb limit. Indeed, the inset of Fig. S6 shows that the ideal value of suppressed V_0/V_1 remains relatively constant $\simeq 1.2 - 1.35$ across

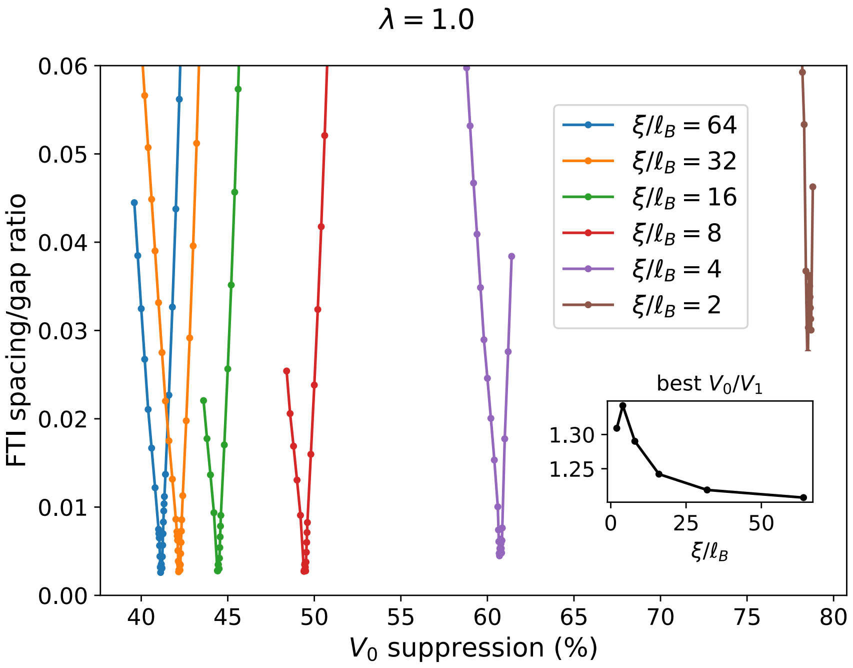

FIG. S6. **FTI spacing/gap ratio as a function of V_0 suppression for different dual-gate Coulomb screening lengths ξ/ℓ_B in the LLL model at $\nu_+ = \nu_- = 1/3$.** A V_0^{+-} suppression of 0% corresponds to the original unaltered dual-gate screened Coulomb potential. The inset shows the effective (suppressed) value of V_0/V_1 for the FTI with the lowest spacing/gap ratio, as a function of ξ/ℓ_B. The interaction potential is isotropic in valley space ($\lambda = 1$), such that $V_m^{++} = V_m^{+-}$. Square torus geometry with $N_\Phi = 15$ flux quanta.

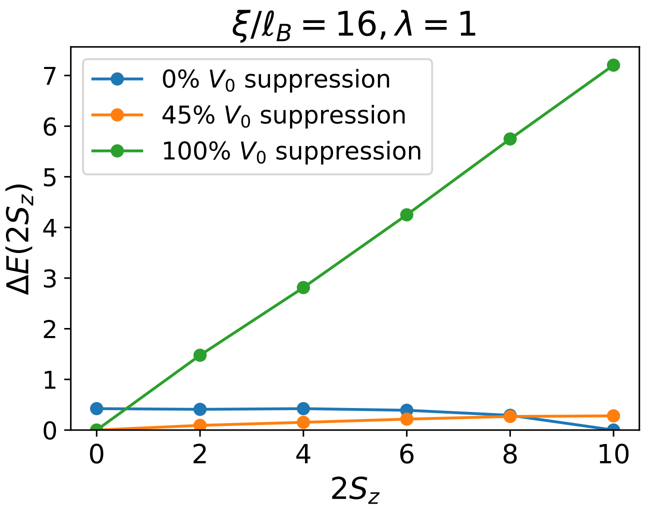

FIG. S7. **Ground state energy across different magnetization sectors in the LLL model with dual-gate screening length $\xi/\ell_B = 16$ at $\nu_+ + \nu_- = 2/3$.** $2S_z = N_+ - N_-$ is the valley imbalance, and $\Delta E(2S_z)$ gives the ground state energy in valley sector S_z measured relative to the minimum energy across all S_z. For 45% V_0 suppression (orange), the system realizes an FTI phase in the $S_z = 0$ sector, and the lowest excitation about the FTI ground state manifold remains in the $S_z = 0$ sector. Square torus geometry with $N_\Phi = 15$ flux quanta.

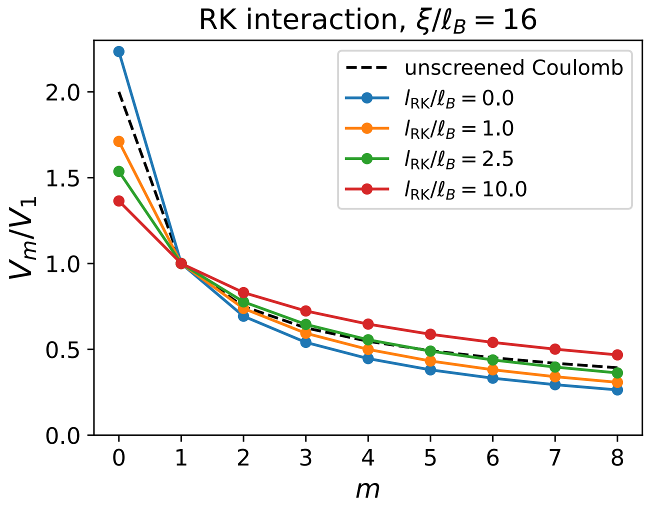

FIG. S8. **LLL pseudopotentials V_m of the dual-gate screened Coulomb interaction with RK corrections (Eq. E7) for $\xi/\ell_B = 16$.** Dashed line corresponds to the unscreened Coulomb limit ($\xi \to \infty, l_{\mathrm{RK}} = 0$).

a wide range of screening lengths. We also find the FTI is more robust for larger ξ, as evidenced by the minimum spacing/gap ratio $\lesssim 0.03$, compared with $\simeq 0.25$ for the effective $\xi \to 0$ limit in Supplementary Note 1 B. Thus, it appears a longer screening length is preferable for obtaining FTIs because less suppression of V_0 is necessary, and the resulting states have a better spacing/gap ratio. This conclusion is somewhat surprising because if we consider a single decoupled valley sector, the FQH state at $\nu = 1/3$ is closer to the ideal Laughlin state for shorter ξ/ℓ_B since the pseudopotentials decay more rapidly (though for the pure Coulomb limit $\xi \to \infty$, the Laughlin state still faithfully captures the low-energy physics there). This suggests, again, that the conditions for realizing an ideal FQH state do not strictly coincide with those for stabilizing an FTI for $\lambda = 1$. Corresponding data for $N_\Phi = 18$, $\xi/\ell_B = 16$ are shown in Fig. 2b of the main text.

Finally, we investigate the dependence of the ground state energy on valley polarization $S_z = \frac{1}{2}(N_+ - N_-)$ at fixed total filling $\nu = 2/3$. Figure S7 shows that for $\xi/\ell_B = 16$, using the unaltered gate-screened interaction leads to the global ground state (which is an FQH state) being in the fully valley-polarized sector. We expect a suppression of V_0 to favor valley depolarization, as the short-range repulsion between opposite-valley fermions is reduced. For 45% V_0 suppression, corresponding to an FTI in the $S_z = 0$ sector, we indeed find that the global ground state is valley unpolarized. Furthermore, the lowest energy state above the FTI manifold is also in the $S_z = 0$ subspace. These findings are unchanged for the range of $\xi/\ell_B = 2 - 64$ investigated.

D. Rytova-Keldysh correction

Appendix 1 C demonstrated that for long-range gate-screened Coulomb interactions $V_\xi(q)$, the FTI can be stabilized if there is an additional short-range attraction that suppresses the V_0 pseudopotential relative to the higher V_m. In this section, we consider a Rytova-Keldysh (RK) correction to the long-range interaction that accounts for the in-plane polarizability, which is affected by the sample thickness, in the systems of interest. We use the following interaction potential

$$V_{\xi, l_{\mathrm{RK}}}(q) = \frac{e^2}{2\epsilon_0 \epsilon q (1 + l_{\mathrm{RK}} q)} \tanh \frac{q\xi}{2} \tag{E7}$$

where l_{RK} is a length scale which will be treated as a phenomenological tuning parameter in the LLL model. Setting $l_{\mathrm{RK}} = 0$ recovers Eq. (E6), while a large l_{RK} weakens the interaction at short length scales, effectively reducing V_0. The interaction at large distances remains dominated by the screening from the metallic gates. Figure S8 illustrates the suppression of V_0/V_1, as well as the slower decay of higher pseudopotentials with m, when l_{RK} is finite. We have checked that for $\nu_+ = 1/3, \nu_- = 0$, the many-body gap above the FQH ground states at l_{RK} does not vanish as l_{RK} is increased.

Figure S9 shows the FTI spacing/gap ratio as a function of the RK length l_{RK} and the suppression of V_0, for $N_\Phi = 15$, gate distance $\xi/\ell_B = 16$ and different values of the valley anisotropy λ [Eq. (E4)]. As l_{RK} increases, the

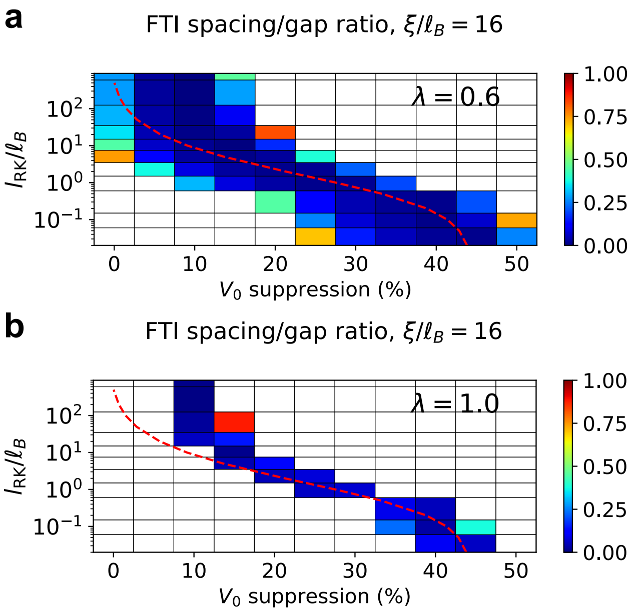

FIG. S9. **FTI spacing/gap ratio as a function of V_0 suppression and RK length $l_{\mathbf{RK}}$ for different interaction anisotropy parameters λ in the LLL model at $\nu_+ = \nu_- = 1/3$.** Gate screening length $\xi/\ell_B = 16$, and $\lambda = 0.6, 1.0$ in **a)** and **b)** respectively. White regions correspond to where the spacing is greater than the gap, or the gap is negative. Red dashed line is contour of constant $V_0/V_1 = 1.24$. Square torus geometry with $N_\Phi = 15$ flux quanta.

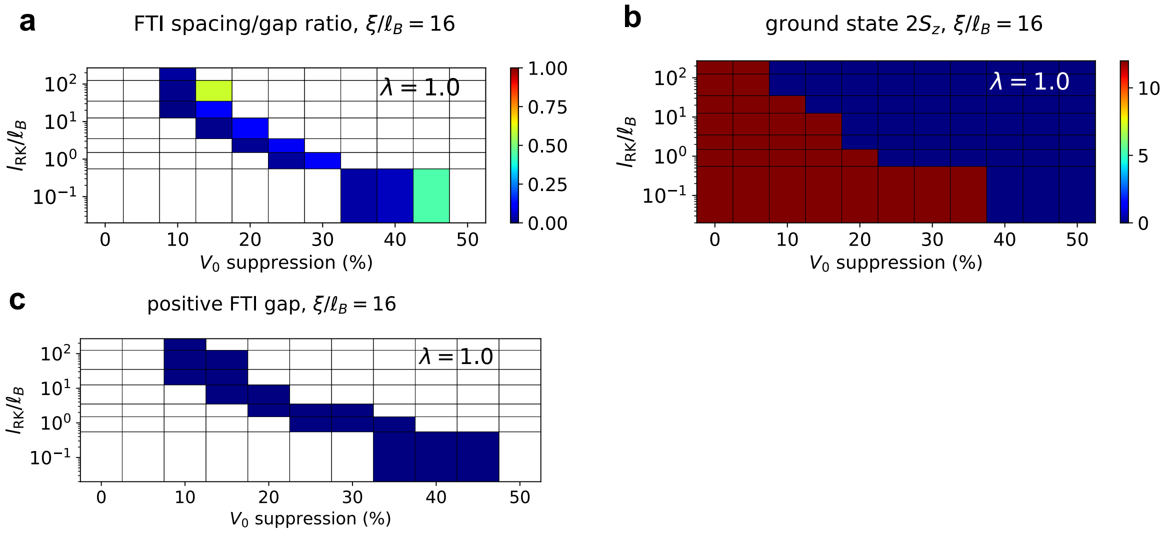

FIG. S10. **FTI spacing/gap ratio, ground state valley polarization, and presence of positive FTI gap as a function of V_0 suppression and RK length $l_{\mathbf{RK}}$ for $\lambda = 1.0$ in the LLL model at $\nu_+ = \nu_- = 1/3$.** Gate screening length $\xi/\ell_B = 16$. **a)** FTI spacing/gap ratio for the unpolarized $S_z = 0$ sector. White regions correspond to where the spacing is greater than the gap, or the gap is negative. The data plotted here is identical to Fig. 2c of the main text. **b)** Ground state valley polarization $2S_z$. **c)** Blue regions indicate where FTI gap $\Delta_9 > 0$, i.e. the nine lowest energy states lie in the correct momentum sectors for an FTI. Square torus geometry with $N_\Phi = 18$ flux quanta.

requisite percentage suppression of V_0 needed to stabilize the FTI decreases. Up to moderate l_{RK}, the parameters that minimize the spacing/gap ratio correspond roughly to a constant value of $V_0/V_1 \simeq 1.24$, though there are deviations for larger l_{RK}.

In Fig. S10a we show the corresponding $\lambda = 1.0$ results for $N_\Phi = 18$ (the data is identical to Fig. 2c of the main text). Furthermore, in Fig. S10b, we plot the valley polarization $2S_z$ of the global ground state across all magnetization sectors. There is a transition between the fully valley-polarized ($2Sz = N_+ + N_-$) phase and the

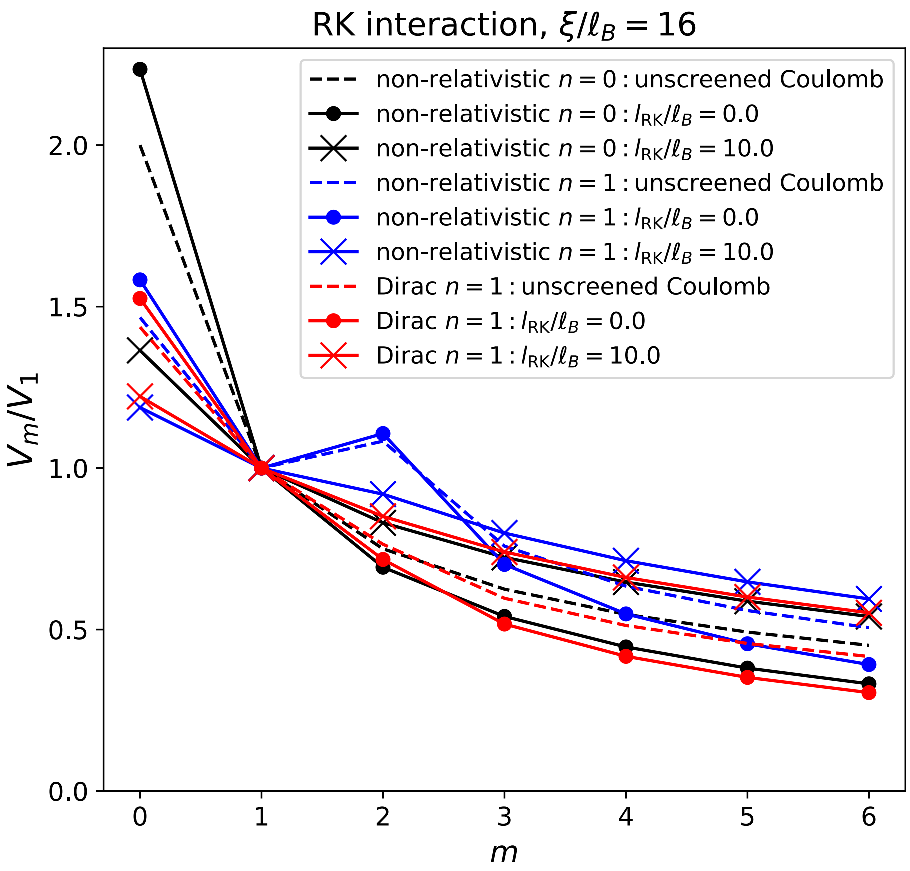

FIG. S11. **Pseudopotentials V_m of the dual-gate screened Coulomb interaction with RK corrections (Eq. E7) for $\xi/\ell_B = 16$ and different Landau levels.** The non-relativistic $n = 1$ LL is shown in blue, while the Dirac $n = 1$ LL is shown in red. The results for the LLL, i.e. the $n = 0$ LL (the pseudopotentials are identical for the non-relativistic and relativistic cases), are shown in black (see also Fig. S8). Dashed lines correspond to the unscreened Coulomb limit ($\xi \to \infty, l_{\rm RK} = 0$).

unpolarized ($2S_z = 0$) phase, which is close to and sometimes overlaps the FTI stability region for $S_z = 0$. The system tends towards polarization for smaller $l_{\rm RK}$ and V_0 suppression. We find that most of the parameters for which we find an FTI in the $S_z = 0$ sector belong to the unpolarized phase. In Fig. S10c, we show the region of parameter space where the FTI gap $\Delta_9 > 0$ is positive, which indicates that the lowest nine energy states have the correct momenta for an FTI. Comparing to Fig. S10a, we observe that this region is not significantly larger than the region where $s_9/\Delta_9 < 1$, suggesting that the FTI stability region is primarily determined by having a positive Δ_9.

E. Other Landau levels

So far, we have only investigated the LLL, which we can also refer to as the 'non-relativistic' $n = 0$ Landau level (LL). The term 'non-relativistic' refers to the fact that the LLs are computed for a parabolic dispersion. We can also project instead into higher LLs with LL index $n > 0$. For a given interaction potential $V(q)$, the corresponding Haldane potentials in the non-relativistic n'th LL are

$$V_{n,m}^{\text{non-rel}} \equiv \int \frac{d^2\mathbf{q}}{(2\pi)^2} V(q) \left[L_n\left(\frac{q^2\ell_B^2}{2}\right)\right]^2 L_m(q^2\ell_B^2) e^{-q^2\ell_B^2}, \tag{E8}$$

which generalize the $n = 0$ case of Eq. (E5). Note that we have made the valley indices η, η' implicit for notational clarity, though just as in Eq. (E4), we can consider scaling the intervalley interaction relative to the intravalley interaction by a factor λ. The additional Laguerre polynomial factors L_n reflect the different form factors of the n'th harmonic oscillator states in the LL problem.

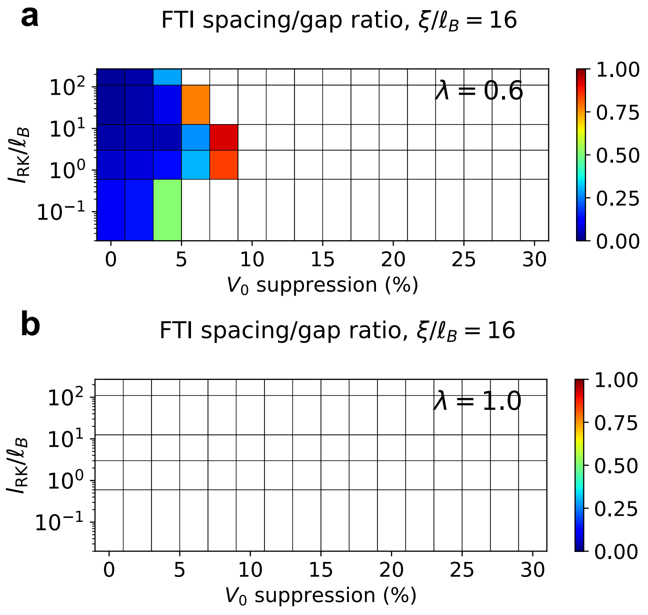

FIG. S12. **FTI spacing/gap ratio as a function of V_0 suppression and RK length l_{RK} for different interaction anisotropy parameters λ in the non-relativistic $n = 1$ LL at $\nu_+ = \nu_- = 1/3$.** Gate screening length $\xi/\ell_B = 16$, and $\lambda = 0.6, 1.0$ in **a)** and **b)** respectively. White regions correspond to where the spacing is greater than the gap, or the gap is negative. Square torus geometry with $N_\Phi = 15$ flux quanta.

We can also consider the 'relativistic' case corresponding to a Dirac Hamiltonian, which would be appropriate for e.g. one valley of graphene where the Dirac Hamiltonian acts in sublattice space [93]. In the presence of a magnetic field, we obtain so-called Dirac LLs. The Dirac $n = 0$ LL consists of the 0'th harmonic oscillator state localized on one sublattice. However, all higher Dirac $n > 0$ LLs consist of an equal magnitude superposition of an n'th harmonic oscillator state in one sublattice and an $(n-1)$'th harmonic oscillator state in the other sublattice. Assuming that the interaction is density-density in sublattice space, we can define the relativistic Haldane pseudopotentials

$$V^{\mathrm{rel}}_{n>0,m} \equiv \int \frac{d^2\mathbf{q}}{(2\pi)^2} V(q) \left[\frac{1}{2} L_n\left(\frac{q^2\ell_B^2}{2}\right) + \frac{1}{2} L_{n-1}\left(\frac{q^2\ell_B^2}{2}\right)\right]^2 L_m(q^2\ell_B^2) e^{-q^2\ell_B^2}, \tag{E9}$$

with $V^{\mathrm{rel}}_{0,m} = V^{\mathrm{non\text{-}rel}}_{0,m}$.

Figure S11 compares the pseudopotential ratios V_m/V_1 for the LLL (where the non-relativistic and Dirac cases are identical), the non-relativistic $n = 1$ LL, and the Dirac $n = 1$ LL. For the same interaction potential $V(q)$, we find that the $n = 1$ LLs have a significantly suppressed V_0/V_1 ratio compared to the LLL. Based on the ED results presented in this appendix so far, this suggests that a comparatively smaller suppression of the V_0 pseudopotential would be needed to stabilize the FTI. In the presence of RK corrections with $l_{\mathrm{RK}}/\ell_B = 10$, the V_0/V_1 ratios of the $n = 1$ LLs are $\simeq 1.2$. However, the $n = 1$ LLs differ significantly in their pseudopotential ratios for $m > 1$. For the (gate-screened) Coulomb interaction, the non-relativistic $n = 1$ LL has sizable $V_2/V_1 > 1$. Furthermore, with the RK correction, V_m/V_1 for $m > 1$ are all moderately enhanced compared to the LLL case. On the other hand, the pseudopotential ratios V_m/V_1 for $m > 1$ in the Dirac $n = 1$ LL are all similar to the those in the LLL. Motivated by our observations of the LL dependence of the pseudopotentials, in the following we perform ED calculations at $\nu_+ = \nu_- = 1/3$ analogous to the LLL model, except that we project instead into the non-relativistic or Dirac $n = 1$ LL.

In Fig. S12, we show the FTI spacing/gap ratio as a function of the RK length l_{RK} and the suppression of V_0, for $N_\Phi = 15$, gate distance $\xi/\ell_B = 16$ and different values of the valley anisotropy λ [Eq. (E4)] for the non-relativistic $n = 1$ LL. For $\lambda = 0.6$, we observe that the FTI phase is clustered around zero or small percentage suppression of V_0. This is to be contrasted with the LLL case (Fig. S9), where at small l_{RK}, a sizable suppression $\gtrsim 25\%$ is required to stabilize the FTI. However for the non-relativistic $n = 1$ LL, we do not find any FTIs for $\lambda = 1.0$. This suggests that the behavior of the pseudopotentials for $m > 1$ prevents the existence of the FTI, even if we tune V_0/V_1. In Fig. S13, we show the FTI spacing/gap ratio and ground state valley polarization for $\lambda = 1.0$ and $N_\Phi = 18$. We note that, like in the LLL (Fig. S10b), there is a phase boundary between the valley-polarized and unpolarized phases. However, here there is no FTI region in the $S_z = 0$ sector anywhere.

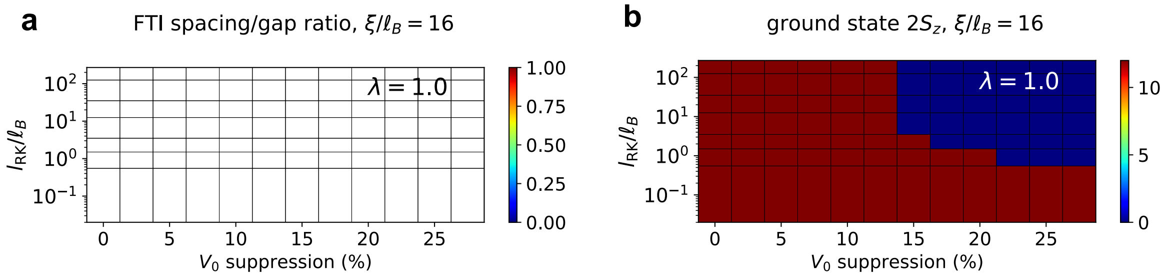

FIG. S13. **FTI spacing/gap ratio and ground state valley polarization as a function of V_0 suppression and RK length $l_{\rm RK}$ for $\lambda = 1.0$ in the non-relativistic $n = 1$ LL at $\nu_+ = \nu_- = 1/3$.** Gate screening length $\xi/\ell_B = 16$. **a)** FTI spacing/gap ratio for the unpolarized $S_z = 0$ sector. White regions correspond to where the spacing is greater than the gap, or the gap is negative. **b)** Ground state valley polarization $2S_z$. Square torus geometry with $N_\Phi = 18$ flux quanta.

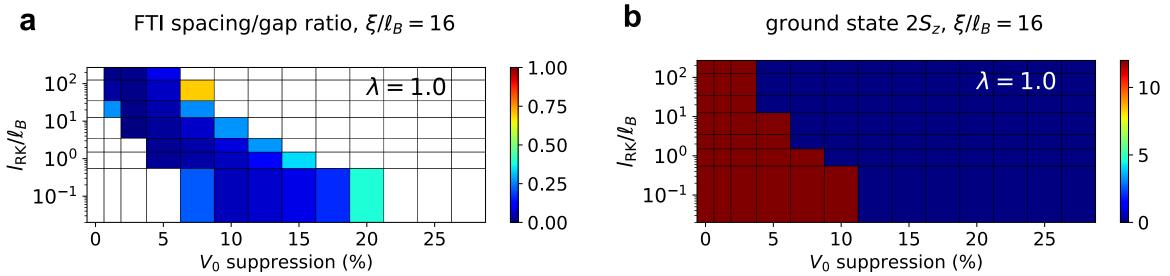

FIG. S14. **FTI spacing/gap ratio and ground state valley polarization as a function of V_0 suppression and RK length $l_{\rm RK}$ for $\lambda = 1.0$ in the Dirac $n = 1$ LL at $\nu_+ = \nu_- = 1/3$.** Gate screening length $\xi/\ell_B = 16$. **a)** FTI spacing/gap ratio for the unpolarized $S_z = 0$ sector. White regions correspond to where the spacing is greater than the gap, or the gap is negative. **b)** Ground state valley polarization $2S_z$. Square torus geometry with $N_\Phi = 18$ flux quanta.

In Fig. S14, we show $N_\Phi = 18$ and $\lambda = 1.0$ results for the Dirac $n = 1$ LL. In the $S_z = 0$ sector, we observe that compared to the LLL case (Fig. S9), the FTI phase exists at a significantly lower range of V_0 percentage suppression. For large $l_{\rm RK}$, the FTI survives very close to the limit where V_0 is not suppressed at all. Our calculations for different valley sectors reveals that for some of the parameters where the $S_z = 0$ sector is an FTI, the global ground state is actually magnetized at $\nu_+ + \nu_- = 2/3$. However, a sizable portion of the parameters where the $S_z = 0$ sector yields an FTI remains non-magnetized.

Supplementary Note 2: Review of tMoTe$_2$ model

In this section, we review the interacting model of tMoTe$_2$ used in this work. In Supplementary Note 2 A, we describe the single-particle continuum model. In Supplementary Note 2 B, we consider the general form of the interaction term.

A. Single-particle continuum model

Our description of the single-particle model closely follows the presentation of Ref. [22]. The single-particle model, which captures the moiré band structure of the valence bands of twisted homobilayer MoTe$_2$, is defined in terms of continuum degrees of freedom associated with electron creation operators $c^\dagger_{\eta,l,\boldsymbol{r}}$. The index $\eta=\pm$ labels the valleys $\pm K$, which are locked to the spins $\uparrow,\downarrow$ due to the strong spin-orbit coupling, while $l=t,b$ labels the two layers, and $\boldsymbol{r}$ labels the in-plane position $\boldsymbol{r}=(x,y)$. The top (bottom) layer is rotated by $-\theta/2$ $(\theta/2)$ starting from AA-stacking, such that the rotated K-points lie at the momenta

$$K_b=\frac{4\pi}{3a_0}\begin{pmatrix}\cos\theta/2\\ \sin\theta/2\end{pmatrix},\quad K_t=\frac{4\pi}{3a_0}\begin{pmatrix}\cos\theta/2\\ -\sin\theta/2\end{pmatrix}, \tag{E1}$$

where $a_0=0.352\,\text{nm}$ is the lattice constant of MoTe$_2$. K_b (K_t) maps onto the K_M (K'_M) point of the moiré Brillouin zone (mBZ). We also define the following wavevectors

$$\boldsymbol{q}_1=K_b-K_t,\quad \boldsymbol{q}_2=C_3\boldsymbol{q}_1,\quad \boldsymbol{q}_3=C_3^2\boldsymbol{q}_1, \tag{E2}$$

where C_3 is a counter-clockwise rotation matrix by $2\pi/3$. In terms of these, we define the basis moiré reciprocal lattice vectors

$$\boldsymbol{b}_1=\boldsymbol{q}_3-\boldsymbol{q}_2,\quad \boldsymbol{b}_2=\boldsymbol{q}_1-\boldsymbol{q}_2. \tag{E3}$$

The continuum model for valley η takes the general form

$$H_{\eta,0}=\int d^2\boldsymbol{r}\begin{pmatrix}c^\dagger_{\eta,b,\boldsymbol{r}} & c^\dagger_{\eta,t,\boldsymbol{r}}\end{pmatrix}\begin{pmatrix}h_{\eta,b}(\boldsymbol{r}) & t_\eta(\boldsymbol{r})\\ t^*_\eta(\boldsymbol{r}) & h_{\eta,t}(\boldsymbol{r})\end{pmatrix}\begin{pmatrix}c_{\eta,b,\boldsymbol{r}}\\ c_{\eta,t,\boldsymbol{r}}\end{pmatrix}. \tag{E4}$$

The intralayer term is

$$h_{\eta,l}(\boldsymbol{r})=\frac{\hbar^2\nabla^2}{2m^*}+V_{\eta,l}(\boldsymbol{r})+(-1)^l\frac{D}{2}, \tag{E5}$$

where $m^*=0.6m_e$ is the effective mass of the valence band maximum of monolayer MoTe$_2$, $V_{\eta,l}(\boldsymbol{r})$ is the intralayer potential to be specified shortly, and D corresponds to an externally applied displacement field. Unless otherwise stated, we set $D=0$. Note that the continuum model operators are defined with a layer-dependent momentum boost. In particular, under the action of translation $T_{\boldsymbol{R}_M}$ by a moiré lattice vector $\boldsymbol{R}_M$, we have

$$T_{\boldsymbol{R}_M}c^\dagger_{\eta,l,\boldsymbol{r}}T^{-1}_{\boldsymbol{R}_M}=c^\dagger_{\eta,l,\boldsymbol{r}+\boldsymbol{R}_M}e^{-i\eta\boldsymbol{R}_M\cdot K_l}. \tag{E6}$$

Both the intralayer moiré potential $V_{\eta,l}(\boldsymbol{r})$ and interlayer hopping $t_\eta(\boldsymbol{r})$ are expanded to the first harmonics

$$V_{\eta,l}(\boldsymbol{r})=Ve^{-(-1)^l i\psi}\sum_{i=1,2,3}e^{i\boldsymbol{g}_i\cdot\boldsymbol{r}}+Ve^{(-1)^l i\psi}\sum_{i=1,2,3}e^{-i\boldsymbol{g}_i\cdot\boldsymbol{r}} \tag{E7}$$

$$t_\eta(\boldsymbol{r})=w\sum_{i=1,2,3}e^{-i\eta\boldsymbol{q}_i\cdot\boldsymbol{r}} \tag{E8}$$

where $(-1)^t=1$ and $(-1)^b=-1$, and $\boldsymbol{g}_i=C_3^{i-1}\boldsymbol{b}_1$. Unless otherwise stated, we use the parameters $w=-18.8\,\text{meV}$, $V=16.5\,\text{meV}$ and $\psi=-105.9°$, which are determined by fitting the above parameterization to DFT calculations [22].

B. Interactions

In this work we consider generally layer-dependent density-density interactions. Since we are considering hole-doping of the moiré valence bands (fully filled valence bands corresponds to $\nu = 0$, i.e. charge neutrality), the interaction Hamiltonian is more naturally expressed in the hole basis as explained in Ref. [19]. We denote by $\tilde{c}^\dagger_{\eta,l,\boldsymbol{r}}$ the hole creation operator, which is related to the electron creation operator by

$$\mathcal{K} c^\dagger_{\eta,l,\boldsymbol{r}} \mathcal{K}^{-1} = \tilde{c}_{\eta,l,\boldsymbol{r}}, \tag{E9}$$

with $\mathcal{K}$ the complex conjugate. We then define the hole density operator $\tilde{\rho}_{\eta,l}(\boldsymbol{q})$ for a fixed valley and layer

$$\tilde{\rho}_{\eta,l}(\boldsymbol{q}) = \sum_{\boldsymbol{k}\in\text{mBZ},\boldsymbol{Q}\in\mathcal{Q}_{\eta,l}} \tilde{c}^\dagger_{\eta,l,\boldsymbol{k}-\boldsymbol{Q}+\boldsymbol{q}} \tilde{c}_{\eta,l,\boldsymbol{k}-\boldsymbol{Q}} \tag{E10}$$

$$\tilde{c}^\dagger_{\eta,l,\boldsymbol{r}} = \frac{1}{\sqrt{\mathcal{V}}} \sum_{\boldsymbol{k}} \sum_{\boldsymbol{Q}\in\mathcal{Q}^\eta_l} e^{-i(\boldsymbol{k}-\boldsymbol{Q})\cdot\boldsymbol{r}} \tilde{c}^\dagger_{\eta,l,\boldsymbol{k}-\boldsymbol{Q}} \tag{E11}$$

$$\mathcal{Q}^\eta_l = \{\boldsymbol{G}_M + \eta(-)^l \boldsymbol{q}_1\}, \tag{E12}$$

where $\mathcal{V}$ is the total area of the system, and $\boldsymbol{G}_M$ is a moiré reciprocal lattice vector. We have introduced the hole creation operator in momentum space $\tilde{c}^\dagger_{\eta,l,\boldsymbol{k}-\boldsymbol{Q}}$. The interaction term takes the form

$$H_{\text{int}} = \frac{1}{2\mathcal{V}} \sum_{\boldsymbol{q}ll'\eta\eta'} V_{ll'}(\boldsymbol{q}) : \tilde{\rho}_{\eta,l}(\boldsymbol{q}) \tilde{\rho}_{\eta',l'}(-\boldsymbol{q}) : \tag{E13}$$

where $V_{ll'}(\boldsymbol{q})$ is the Fourier transform of the layer-dependent interaction potential. The total Hamiltonian is $H = H_{\text{int}} + \sum_\eta H_{\eta,0}$.

The notation $:\hat{O}:$ in Eq. (E13) denotes a normal-ordering of the operators in $\hat{O}$ with respect to the hole vacuum. This means that all hole creation operators $\tilde{c}^\dagger$ appear to the left of all hole annihilation operators $\tilde{c}$. Therefore, H_{int} annihilates the state $|\nu = 0\rangle$ at charge neutrality at filling factor $\nu = 0$, which corresponds to having no holes in the system. This form of the interaction is sensible because the continuum model parameters are extracted from DFT calculations performed at charge neutrality.

In this work, we consider two contributions to the interaction potential $V_{ll'}(\boldsymbol{q})$. The main contribution is the long-range Coulomb interaction, which is screened by the metallic gates and the non-local dielectric environment of the $t\text{MoTe}_2$ device. This is discussed in detail in Supplementary Note 4. To help stabilize the FTI, we also add an on-site intralayer attractive interaction with amplitude g (see Eq. (3) in the main text and Eq. (E1)). Due to fermion antisymmetry, this only has an effect between particles with different spins. One possible origin of an attractive g is the electron-phonon coupling, which is studied in Supplementary Note 3.

We can characterize the interaction potential $V_{ll'}(\boldsymbol{q})$ by calculating the Haldane pseudopotentials in the case that the it is projected into the LLL. This is carried out by considering Eq. E5 with the effective magnetic length $\ell^*_B = 2.02\,\text{nm}$. This value of ℓ^*_B is chosen to satisfy [69] $2\pi{\ell^*_B}^2 = A_M$, with A_M the moiré unit cell area of $t\text{MoTe}_2$ at $\theta = 3.7°$.

Supplementary Note 3: Contribution to short-range interaction from electron-phonon coupling in tMoTe$_2$

In this section, we discuss a possible physical origin for the onsite interaction g in Eq. (3). More explicitly, the onsite interaction has the following form

$$H_g = \frac{g}{2}\sum_{l\eta\eta'}\int d^2r : \tilde{\rho}_{\eta,l}(\boldsymbol{r})\tilde{\rho}_{\eta',l}(\boldsymbol{r}) := \frac{g}{2\mathcal{V}}\sum_{\boldsymbol{q}l\eta\eta'} : \tilde{\rho}_{\eta,l}(\boldsymbol{q})\tilde{\rho}_{\eta',l}(-\boldsymbol{q}) : , \tag{E1}$$

where the hole density operator $\tilde{\rho}_{\eta,l}(\boldsymbol{q})$ and the normal-ordering notation are defined in Supplementary Note 2 B, and $\tilde{\rho}_{\eta,l}(\boldsymbol{r})$ is the Fourier transformation of $\tilde{\rho}_{\eta,l}(\boldsymbol{q})$:

$$\tilde{\rho}_{\eta,l}(\boldsymbol{r}) = \tilde{c}^\dagger_{\eta,l,\boldsymbol{r}}\tilde{c}_{\eta,l,\boldsymbol{r}} \ . \tag{E2}$$

We note that the intravalley component of Eq. (E1) is ineffective due to the onsite and intralayer nature of the interaction, as fermionic statistics leads to

$$: \tilde{\rho}_{\eta,l}(\boldsymbol{r})\tilde{\rho}_{\eta,l}(\boldsymbol{r}) := \tilde{c}^\dagger_{\boldsymbol{r},\eta,l}\tilde{c}^\dagger_{\boldsymbol{r},\eta,l}\tilde{c}_{\boldsymbol{r},\eta,l}\tilde{c}_{\boldsymbol{r},\eta,l} = 0 \ . \tag{E3}$$

As such, g mimics the effect of the V_0 Haldane pseudopotential in the LLL model discussion of Supplementary Note 1. As shown in the main text and Supplementary Note 5, such a short-range interaction with attractive $g < 0$ is required to stabilize the FTI phase in the ED calculations of tMoTe$_2$. In the following, we will derive the contribution to g from the electron-phonon coupling (EPC).

The EPC that we consider is within each individual MoTe$_2$ layer. The low-energy physics of the monolayer MoTe$_2$ can be well captured by d orbitals on Mo atoms and the p orbitals on the Te atoms [22, 65]. The single-layer MoTe$_2$ has a mirror symmetry with the mirror plane lying in the 2D system, namely m_z where z is perpendicular to the sample. The low-energy states at and near $\pm$K mainly come from the $d_{x^2-y^2}$ and d_{xy} orbitals at Mo atoms, which are even under m_z. Therefore, we will only consider the fermionic m_z-even sector of the Hamiltonian, leading to the following general EPC Hamiltonian

$$H_{\text{el-ph}} = \sum_{\boldsymbol{R}_1\boldsymbol{R}_2\boldsymbol{R}}\sum_{\boldsymbol{\tau}_1\boldsymbol{\tau}_2\boldsymbol{\tau}}^{\boldsymbol{\tau}_{\text{Mo}},\boldsymbol{\tau}_{\text{Te},1},\boldsymbol{\tau}_{\text{Te},2}}\sum_{\alpha_{\boldsymbol{\tau}_1}\alpha'_{\boldsymbol{\tau}_2}i}\sum_s \hat{c}^\dagger_{\boldsymbol{R}_1+\boldsymbol{\tau}_1,\alpha_{\boldsymbol{\tau}_1},s}\hat{c}_{\boldsymbol{R}_2+\boldsymbol{\tau}_2,\alpha'_{\boldsymbol{\tau}_2},s}u_{\boldsymbol{R}+\boldsymbol{\tau},i}\ F^{\alpha_{\boldsymbol{\tau}_1}\alpha'_{\boldsymbol{\tau}_2}i}_{\boldsymbol{\tau}_1,\boldsymbol{R}_2-\boldsymbol{R}_1+\boldsymbol{\tau}_2,\boldsymbol{R}-\boldsymbol{R}_1+\boldsymbol{\tau}} \ , \tag{E4}$$

where $s=\uparrow,\downarrow$ labels the spin, $\boldsymbol{\tau}_{\text{Mo}}$ labels the position of the Mo atom in one unit cell, $\boldsymbol{\tau}_{\text{Te},1}$ and $\boldsymbol{\tau}_{\text{Te},2}$ label the positions of the two Te atoms in one unit cell, $\alpha_{\boldsymbol{\tau}}$ labels the orbitals on the atom $\boldsymbol{\tau}$, $\hat{c}^\dagger_{\boldsymbol{R}_1+\boldsymbol{\tau}_1,\alpha_{\boldsymbol{\tau}_1},s}$ creates an electron at $\boldsymbol{R}_1+\boldsymbol{\tau}_1$ with orbital $\alpha_{\boldsymbol{\tau}_1}$ and spin s, and $u_{\boldsymbol{R}+\boldsymbol{\tau},i}$ labels the ion motion at $\boldsymbol{R}+\boldsymbol{\tau}$ along the ith direction. Here we use the fact that the dominant contribution from spin-orbit coupling usually only enters in an on-site way, and thus the EPC is diagonal in spin. The lattice translations lead to

$$F^{\alpha_{\boldsymbol{\tau}_1}\alpha'_{\boldsymbol{\tau}_2}i}_{\boldsymbol{R}_1+\boldsymbol{R}_0+\boldsymbol{\tau}_1,\boldsymbol{R}_2+\boldsymbol{R}_0+\boldsymbol{\tau}_2,\boldsymbol{R}+\boldsymbol{R}_0+\boldsymbol{\tau}} = F^{\alpha_{\boldsymbol{\tau}_1}\alpha'_{\boldsymbol{\tau}_2}i}_{\boldsymbol{R}_1+\boldsymbol{\tau}_1,\boldsymbol{R}_2+\boldsymbol{\tau}_2,\boldsymbol{R}+\boldsymbol{\tau}}\ \forall \text{ lattice vector } \boldsymbol{R}_0 \ . \tag{E5}$$

Transforming Eq. (E4) to the momentum space, we have

$$H_{\text{el-ph}} = \frac{1}{N}\sum_{\boldsymbol{\tau}_1,\boldsymbol{\tau}_2,\boldsymbol{\tau}}\sum_{\alpha_{\boldsymbol{\tau}_1}\alpha'_{\boldsymbol{\tau}_2}i}\sum_s\sum_{\boldsymbol{k}_1,\boldsymbol{k}_2}^{\text{1BZ}} \hat{c}^\dagger_{\boldsymbol{k}_1,\boldsymbol{\tau}_1,\alpha_{\boldsymbol{\tau}_1},s}\hat{c}_{\boldsymbol{k}_2,\boldsymbol{\tau}_2,\alpha'_{\boldsymbol{\tau}_2},s}\left[F_{\boldsymbol{\tau}i}(\boldsymbol{k}_1,\boldsymbol{k}_2)\right]_{\boldsymbol{\tau}_1\alpha_{\boldsymbol{\tau}_1},\boldsymbol{\tau}_2\alpha'_{\boldsymbol{\tau}_2}} u^\dagger_{\boldsymbol{k}_2-\boldsymbol{k}_1,\boldsymbol{\tau},i} \ , \tag{E6}$$

where 1BZ is the first Brillouin zone of the monolayer MoTe$_2$, and

$$\left[F_{\boldsymbol{\tau}i}(\boldsymbol{k}_1,\boldsymbol{k}_2)\right]_{\boldsymbol{\tau}_1\alpha_{\boldsymbol{\tau}_1},\boldsymbol{\tau}_2\alpha'_{\boldsymbol{\tau}_2}} = \sum_{\boldsymbol{R}_1\boldsymbol{R}_2} e^{-\mathrm{i}\boldsymbol{k}_1\cdot(\boldsymbol{R}_1+\boldsymbol{\tau}_1-\boldsymbol{\tau})}e^{\mathrm{i}\boldsymbol{k}_2\cdot(\boldsymbol{R}_2+\boldsymbol{\tau}_2-\boldsymbol{\tau})}F^{\alpha_{\boldsymbol{\tau}_1}\alpha'_{\boldsymbol{\tau}_2}i}_{\boldsymbol{R}_1+\boldsymbol{\tau}_1,\boldsymbol{R}_2+\boldsymbol{\tau}_2,\boldsymbol{\tau}} \ . \tag{E7}$$

For the moment, we focus on momenta $\pm$K for the fermions, since the relevant low-energy fermionic degrees of freedom lie around the K valleys. We then have

$$H_{\text{el-ph}} \approx \frac{1}{N}\sum_{\eta_1\eta_2}\sum_{\boldsymbol{\tau}_1,\boldsymbol{\tau}_2,\boldsymbol{\tau}}\sum_{\alpha_{\boldsymbol{\tau}_1}\alpha'_{\boldsymbol{\tau}_2}i}\sum_s \hat{c}^\dagger_{\eta_1\text{K},\boldsymbol{\tau}_1,\alpha_{\boldsymbol{\tau}_1},s}\hat{c}_{\eta_2\text{K},\boldsymbol{\tau}_2,\alpha'_{\boldsymbol{\tau}_2},s}u^\dagger_{(\eta_2-\eta_1)\text{K},\boldsymbol{\tau},i}\left[F_{\boldsymbol{\tau}i}(\eta_1\text{K},\eta_2\text{K})\right]_{\boldsymbol{\tau}_1\alpha_{\boldsymbol{\tau}_1},\boldsymbol{\tau}_2\alpha'_{\boldsymbol{\tau}_2}} \ . \tag{E8}$$

After integrating out the fermions, the EPC would eventually lead to an effective attractive interaction. To show this, we first convert the EPC Hamiltonian to the eigenbasis of electrons $\gamma^\dagger$ and phonons $b^\dagger$, which reads

$$H_{\text{el-ph}} = \frac{1}{N}\sum_{nmls}\sum_{\eta_1\eta_2=\pm} G^s_{nml}(\eta_1\mathrm{K},\eta_2\mathrm{K})\gamma^\dagger_{\eta_1\mathrm{K},n,s}\gamma_{\eta_2\mathrm{K},m,s}(b^\dagger_{(\eta_2-\eta_1)\mathrm{K},a}+b_{-(\eta_2-\eta_1)\mathrm{K},a}) \ , \tag{E9}$$

where

$$\widetilde{F}_a(\eta_1\mathrm{K},\eta_2\mathrm{K}) = \sum_{\boldsymbol{\tau}',i'} F_{\boldsymbol{\tau}'i'}(\eta_1\mathrm{K},\eta_2\mathrm{K})\frac{1}{m_{\boldsymbol{\tau}'}}[v^*_a(\eta_2\mathrm{K}-\eta_1\mathrm{K})]_{\boldsymbol{\tau}'i'} \tag{E10}$$

$$\widetilde{G}^s_{nma}(\eta_1\mathrm{K},\eta_2\mathrm{K}) = U^\dagger_{n,s}(\eta_1\mathrm{K})\widetilde{F}_a(\eta_1\mathrm{K},\eta_2\mathrm{K})U_{m,s}(\eta_2\mathrm{K}) \ , \tag{E11}$$

$$G^s_{nma}(\eta_1\mathrm{K},\eta_2\mathrm{K}) = \frac{\hbar}{2\omega_a(\eta_2\mathrm{K}-\eta_1\mathrm{K})}\widetilde{G}^s_{nma}(\eta_1\mathrm{K},\eta_2\mathrm{K}) \ , \tag{E12}$$

and $\omega_a(\boldsymbol{q})$ and $v_a(\boldsymbol{q})$ are the frequency and eigenvector of the phonon in the ath phonon band and momentum $\boldsymbol{q}$. Here, we have used the spin-U(1) symmetry of the single-particle fermionic Hamiltonian, $U_{m,s}(\boldsymbol{k})$ labels the eigenvector of the mth electronic band with spin s at $\boldsymbol{k}$, and

$$\gamma^\dagger_{\boldsymbol{k},n,s} = \sum_{\boldsymbol{\tau}_1,\alpha_{\boldsymbol{\tau}_1}} \hat{c}^\dagger_{\boldsymbol{k},\boldsymbol{\tau}_1,\alpha_{\boldsymbol{\tau}_1},s}\,[U_{m,s}(\boldsymbol{k})]_{\boldsymbol{\tau}_1,\alpha_{\boldsymbol{\tau}_1}} \ . \tag{E13}$$

Then, based on the second-order perturbation theory, the EPC can mediate an attractive interaction as the following:

$$\begin{aligned} H_{\text{eff-int}} = \frac{1}{N}\sum_{\eta_1,a}\sum_{nn'mm'ss'}\sum_{\eta,\eta'} &\frac{G^{s'}_{n'm'a}(\eta'\mathrm{K},\eta'\mathrm{K}+\eta\mathrm{K}-\eta_1\mathrm{K})G^s_{nma}(\eta\mathrm{K},\eta_1\mathrm{K})\hbar\omega_a(\eta_1\mathrm{K}-\eta\mathrm{K})}{(E_{ns}(\eta\mathrm{K})-E_{ms}(\eta_1\mathrm{K}))^2-(\hbar\omega_a(\eta_1\mathrm{K}-\eta\mathrm{K}))^2} \\ &\times \gamma^\dagger_{\eta\mathrm{K},n,s}\gamma_{\eta_1\mathrm{K},m,s}\gamma^\dagger_{\eta'\mathrm{K},n',s'}\gamma_{\eta'\mathrm{K}+\eta\mathrm{K}-\eta_1\mathrm{K},m',s'} \ , \end{aligned} \tag{E14}$$

where $E_{ns}(\boldsymbol{k})$ is the energy of the nth electronic band with spin s at $\boldsymbol{k}$. Owing to the spin-orbit coupling, the low-energy electronic states in the top valence band are spin-valley locked—at low energies, we only need to consider the spin-up state at K valley and the spin-down state at −K valley. As a result, Eq. (E14) becomes

$$H_{\text{eff-int}} = \frac{1}{N}\sum_{\boldsymbol{p}\boldsymbol{p}'\boldsymbol{q}}\sum_a\sum_{\eta,\eta'}\frac{G^{\eta'}_{00a}(\eta'\mathrm{K},\eta'\mathrm{K})G^{\eta}_{00a}(\eta\mathrm{K},\eta\mathrm{K})}{-(\hbar\omega_a(0))}\gamma^\dagger_{\eta\mathrm{K}+\boldsymbol{p}+\boldsymbol{q},0,\eta}\gamma_{\eta\mathrm{K}+\boldsymbol{p}',0,\eta}\gamma^\dagger_{\eta'\mathrm{K}+\boldsymbol{p}'-\boldsymbol{q},0,\eta'}\gamma_{\eta'\mathrm{K}+\boldsymbol{p},0,\eta'} \ , \tag{E15}$$

where $n=0$ corresponds to the top valence band with spin s. Note that we have re-introduced the momentum dependence in the fermionic operators, and made the approximation that the EPC-induced interaction is independent of the momentum deviations from $\pm K$. Owing to the TR symmetry, we have

$$G^-_{00a}(-\mathrm{K},-\mathrm{K}) = G^+_{00a}(\mathrm{K},\mathrm{K}) \in \mathbb{R} \ . \tag{E16}$$

By defining

$$g_{EPC} = -2\Omega\sum_a\frac{\left|G^+_{00a}(\mathrm{K},\mathrm{K})\right|^2}{\hbar\omega_a(0)} \tag{E17}$$

with Ω the unit cell area of monolayer $MoTe_2$, we arrive at

$$H_{\text{eff-int}} = \frac{g_{EPC}}{2\mathcal{V}}\sum_{\boldsymbol{p}\boldsymbol{p}'\boldsymbol{q}}\sum_{\eta,\eta'}\gamma^\dagger_{\eta\mathrm{K}+\boldsymbol{p}+\boldsymbol{q},0,\eta}\gamma_{\eta\mathrm{K}+\boldsymbol{p}',0,\eta}\gamma^\dagger_{\eta'\mathrm{K}+\boldsymbol{p}'-\boldsymbol{q},0,\eta'}\gamma_{\eta'\mathrm{K}+\boldsymbol{p},0,\eta'} \ . \tag{E18}$$

The above derivation holds for each individual layer. By restoring the layer index l, we would have $\gamma^\dagger_{\eta\mathrm{K}+\boldsymbol{p},0,\eta,l}$ for each layer l, which is equivalent to the continuum electron creation operator $c^\dagger_{\eta,l,\boldsymbol{p}}$ in momentum space. After including the layer index and by converting $c^\dagger_{\eta,l,\boldsymbol{p}}$ to the real space, $H_{\text{eff-int}}$ becomes

$$H_{\text{eff-int}} = \frac{g_{EPC}}{2}\int d^2r\sum_{l,\eta,\eta'}c^\dagger_{\eta,l,\boldsymbol{r}}c_{\eta,l,\boldsymbol{r}}c^\dagger_{\eta',l,\boldsymbol{r}}c_{\eta',l,\boldsymbol{r}} \ , \tag{E19}$$

which, in the hole basis (Eq. (E9)) reads

$$H_{\text{eff-int}} = \frac{g_{EPC}}{2} \int d^2r \sum_{l,\eta,\eta'} \widetilde{c}_{\eta,l,\boldsymbol{r}} \widetilde{c}^\dagger_{\eta,l,\boldsymbol{r}} \widetilde{c}_{\eta',l,\boldsymbol{r}} \widetilde{c}^\dagger_{\eta',l,\boldsymbol{r}} \ . \tag{E20}$$

In this work, we consider the normal-ordered part of the interaction (see Supplementary Note 2 B). After the normal-ordering, $H_{\text{eff-int}}$ becomes

$$: H_{\text{eff-int}} := \frac{g_{EPC}}{2} \int d^2r \sum_{l,\eta,\eta'} \widetilde{c}^\dagger_{\eta,l,\boldsymbol{r}} \widetilde{c}^\dagger_{\eta',l,\boldsymbol{r}} \widetilde{c}_{\eta',l,\boldsymbol{r}} \widetilde{c}_{\eta,l,\boldsymbol{r}} \ , \tag{E21}$$

which has the same form as Eq. (E1). Therefore, g_{EPC} is the contribution to g from the EPC.

Numerically, Eq. (E17) is hard to evaluate owing to the fact that $\omega_a(0)$ is zero for acoustic modes. Therefore, we use the dimensionless EPC constant Λ to approximately calculate g_{EPC}. Specifically, Λ has the following expression

$$\Lambda = \frac{2}{D(\mu)N} \sum_{\boldsymbol{k},\boldsymbol{k}'}^{\text{1BZ}} \sum_{nmas} \frac{|G^s_{nma}(\boldsymbol{k},\boldsymbol{k}')|^2}{\hbar\omega_a(\boldsymbol{k}'-\boldsymbol{k})} \delta\left(\mu - E^s_n(\boldsymbol{k})\right) \delta\left(\mu - E^s_m(\boldsymbol{k}')\right) \ , \tag{E22}$$

where $E^s_n(\boldsymbol{k})$ is the nth electron band in the spin-s subspace, and $D(\mu) = \sum_{\boldsymbol{k}ns} \delta\left(\mu - E^s_n(\boldsymbol{k})\right)$ is the density of states. For slightly hole-doped case that is relevant for the experiments, the chemical potential is close to the valence band top such that we would have two Fermi surfaces centered around $\pm$K, and Λ becomes

$$\begin{aligned}
\Lambda &= \frac{2}{D(\mu)N} \sum_{\boldsymbol{p}\boldsymbol{p}'} \sum_{\eta\eta'} \sum_{as} \frac{|G^s_{00a}(\eta\text{K}+\boldsymbol{p},\eta'\text{K}+\boldsymbol{p}')|^2}{\hbar\omega_a(\eta'\text{K}+\boldsymbol{p}'-\eta\text{K}-\boldsymbol{p})} \delta\left(\mu - E^s_0(\eta\text{K}+\boldsymbol{p})\right) \delta\left(\mu - E^s_0(\eta'\text{K}+\boldsymbol{p}')\right) \\
&= \frac{2}{D(\mu)N} \sum_{\boldsymbol{p}\boldsymbol{p}'} \sum_{\eta} \sum_{a} \frac{|G^\eta_{00a}(\eta\text{K}+\boldsymbol{p},\eta\text{K}+\boldsymbol{p}')|^2}{\hbar\omega_a(\eta\text{K}+\boldsymbol{p}'-\eta\text{K}-\boldsymbol{p})} \delta\left(\mu - E^\eta_0(\eta\text{K}+\boldsymbol{p})\right) \delta\left(\mu - E^\eta_0(\eta\text{K}+\boldsymbol{p}')\right) \\
&\approx \frac{2}{D(\mu)N} \sum_{\boldsymbol{p}\boldsymbol{p}'} \sum_{\eta} \sum_{a} \frac{|G^\eta_{00a}(\eta\text{K},\eta\text{K})|^2}{\hbar\omega_a(0)} \delta\left(\mu - E^\eta_0(\eta\text{K}+\boldsymbol{p})\right) \delta\left(\mu - E^\eta_0(\eta\text{K}+\boldsymbol{p}')\right) \\
&= \frac{1}{D(\mu)N} \sum_{\eta} \frac{-g_{EPC}}{\Omega} D_\eta(\mu)^2 \ ,
\end{aligned} \tag{E23}$$

where we have used the fact that $\boldsymbol{p}$ on the Fermi surface is small when the filling factor ν is close to the valence band top, and $D_s(\mu) = \sum_{\boldsymbol{k}} \delta\left(\mu - E^s_n(\boldsymbol{k})\right)$. Owing to the TR symmetry, we have $D_+(\mu) = D_-(\mu) = D(\mu)/2$, leading to

$$\Lambda \approx \frac{D(\mu)}{N} \frac{-g_{EPC}}{2\Omega} \Rightarrow g_{EPC} \approx -\frac{2\Omega}{D(\mu)/N}\Lambda \ . \tag{E24}$$

Using the software package EPW [94], one can numerically calculate Λ. For $\mu = -20$meV (we choose the valence band top to correspond to zero energy), we obtain

$$D(\mu)/N \approx 0.306\text{eV}^{-1} \ , \ \text{and } \Lambda \approx 0.0233 \ . \tag{E25}$$

Combined with $\Omega \approx 0.11\text{nm}^2$, we arrive at

$$g_{EPC} \approx -16.8\,\text{meV} \cdot \text{nm}^2 \ . \tag{E26}$$

Supplementary Note 4: Dielectric screening of interaction potential

In this appendix, we discuss the impact of the dielectric environment on the effective interaction potential between electrons in the tMoTe$_2$ sample. We consider a system that is homogeneous in-plane, but is constructed by stacking distinct 'slabs' (for instance, various dielectric materials and the tMoTe$_2$ sample itself) in the out-of-plane z-direction. We first discuss the general formulation of the electrostatics problem and explain how the layer-dependent potential $V_{ll'}(q)$ used in the ED calculations of the main text is generated. We also consider some analytical examples. Then, we perform calculations on various system geometries to understand the impact of the dielectric environment on V_0/V_1, including configurations with additional 'spacer' dielectric slabs.

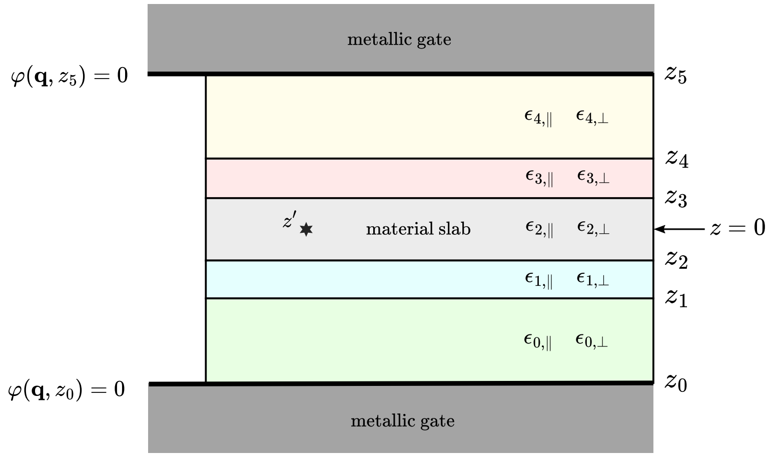

FIG. S15. **Schematic of sample geometry.** This figure considers an example system with $N_{\rm slab} = 5$ slabs, where the material slab is chosen as the middle $i = 2$ slab. The z-axis is centered so that the middle of the material slab is at $z = 0$. Each slab i is characterized by homogeneous dielectric constants $\epsilon_{i,\parallel}, \epsilon_{i,\perp}$, and has its bottom face at $z = z_i$. Perfect metallic gates are positioned at $z = z_0$ and $z = z_5$, and enforce the vanishing of the 2D Fourier-transformed interaction potential $\varphi(\mathbf{q}, z_0) = \varphi(\mathbf{q}, z_5) = 0$. To compute the screened interaction potential within the material slab, we solve the Poisson equation sourced by a point test charge at $z = z'$.

A. General setup

The time-independent Maxwell equations for electrostatics in dielectric media are

$$\nabla_{\tilde{\mathbf{r}}} \cdot \mathbf{D} = \rho_f, \quad \nabla_{\tilde{\mathbf{r}}} \times \mathbf{E} = 0, \tag{E1}$$

where ρ_f is the free charge, $\tilde{\mathbf{r}} = (\mathbf{r}, z)$ is the 3D position, and $\mathbf{r} = (x, y)$ is the 2D in-plane position. To solve the second equation above, we express

$$\mathbf{E} = \nabla_{\tilde{\mathbf{r}}} \phi \tag{E2}$$

in terms of the electric potential ϕ.

We consider a system consisting of $N_{\rm slab}$ slabs of varying thickness d_i that are vertically stacked and indexed by $i = 0, \ldots, N_{\rm slab} - 1$ in order of increasing z. Let z_i be the z-coordinate of the bottom face of slab i, and $z_{N_{\rm slab}}$ be the z-coordinate of the top face of slab $N_{\rm slab} - 1$ (the top slab), so that $d_i = z_{i+1} - z_i$. We place ideal metallic gates at z_0 and $z_{N_{\rm slab}}$, i.e. at the bottom and top of the stack. For a sample with no metallic gates, we send z_0 ($z_{N_{\rm slab}}$) to $-\infty$ ($+\infty$). For a single-gated sample, we send $z_{N_{\rm slab}}$ to $+\infty$. The slab $i = i_m$ is designated as the 'material slab', i.e. tMoTe$_2$, whose screened interaction potential $V_{ll'}(q)$ is of interest. By convention, we will center the z-axis so that the material slab is centered at $z = 0$. The setup for $N_{\rm slab} = 5$ is illustrated in Fig. S15.

Each slab is associated with anisotropic and homogeneous dielectric constants $\epsilon_{i,\parallel}$ and $\epsilon_{i,\perp}$. In terms of these, we define the following constants for convenience

$$\kappa_i = \sqrt{\frac{\epsilon_{i,\parallel}}{\epsilon_{i,\perp}}}, \quad \eta_i = \sqrt{\epsilon_{i,\parallel}\epsilon_{i,\perp}}. \tag{E3}$$

Within slab i, the electric displacement field D is related to the electric field E as

$$D_x = \epsilon_0 \epsilon_{i,\parallel} E_x, \quad D_y = \epsilon_0 \epsilon_{i,\parallel} E_y, \quad D_z = \epsilon_0 \epsilon_{i,\perp} E_z, \tag{E4}$$

where ϵ_0 is the vacuum permittivity. To account for the in-plane translation invariance, we define the 2D Fourier transform of the in-plane coordinates

$$\phi(\mathbf{r}, z) = \int \frac{d^2\boldsymbol{q}}{(2\pi)^2} e^{i\boldsymbol{q}\cdot\boldsymbol{r}} \varphi(\boldsymbol{q}, z), \tag{E5}$$

where $\mathbf{q}$ is the 2D in-plane momentum. Due to the discontinuities in the dielectric environment as a function of z, we define the electric potential within each slab

$$\varphi_i(\mathbf{q}, z) = \varphi(\mathbf{q}, z) \text{ for } z_i \leq z \leq z_{i+1}. \tag{E6}$$

Within slab i, we obtain the Poisson equation from the first equation of Eq. (E1)

$$\epsilon_{i,\perp} \partial_z^2 \varphi_i(\mathbf{q}, z) - \epsilon_{i,\parallel} q^2 \varphi_i(\mathbf{q}, z) = \rho_f(\mathbf{r}, z). \tag{E7}$$

To stitch together the solutions for different slabs, we utilize the boundary conditions

$$\varphi_i(\mathbf{q}, z_{i+1}) = \varphi_{i+1}(\mathbf{q}, z_{i+1}) \tag{E8}$$
$$\epsilon_{i,\perp} \partial_z \varphi_i(\mathbf{q}, z_{i+1}) = \epsilon_{i+1,\perp} \partial_z \varphi_{i+1}(\mathbf{q}, z_{i+1}) \tag{E9}$$

for $i = 0, \ldots N_{\text{slab}} - 2$. For the outermost faces of the system, we assume perfect metallic gates which impose

$$\varphi_0(\mathbf{q}, z_0) = \varphi_{N_{\text{slab}}-1}(\mathbf{q}, z_{N_{\text{slab}}}) = 0. \tag{E10}$$

Note that we could have considered instead metallic gates characterized by e.g. a finite screening length. However, the resulting Poisson equation would be non-local and considerably more complicated to solve. Furthermore, the screening length of graphite is $\sim 1\,\text{nm}$ [95], which is smaller than the typical thicknesses of both the encapsulating hBN substrates and graphite gates in experiments.

In the following, we consider ρ_f to consist of a test charge of magnitude Q positioned at $\mathbf{r} = 0$ and height z' within the material slab $i = i_m$

$$\rho_f(\mathbf{r}, z) = Q\delta(\mathbf{r})\delta(z - z'). \tag{E11}$$

Hence for $i \neq i_m$, we have the general solution

$$\varphi_i(\mathbf{q}, z) = A_i(\mathbf{q}) e^{\kappa_i q z} + B_i(\mathbf{q}) e^{-\kappa_i q z}, \tag{E12}$$

while for $i = i_m$, we also need to include the particular integral

$$\varphi_i(\mathbf{q}, z) = A_i(\mathbf{q}) e^{\kappa_i q z} + B_i(\mathbf{q}) e^{-\kappa_i q z} - \frac{Q}{2\epsilon_0 \eta_i q} e^{-\kappa_i q |z - z'|}. \tag{E13}$$

For each $\mathbf{q}$, we have $2N_{\text{slab}}$ unknowns $A_i(\mathbf{q}), B_i(\mathbf{q})$. There are $2N_{\text{slab}}$ boundary conditions, with $2N_{\text{slab}} - 2$ from the internal slab interfaces, and 2 from the outermost faces. The resulting linear system of equations can be solved to obtain $\varphi(\mathbf{q}, z)$.

In the $t\text{MoTe}_2$ continuum model, we take the microscopic layers to lie at the Mo planes at $z = \pm z_{\text{Mo}} = \pm 0.365\,\text{nm}$. For the ED calculations of $t\text{MoTe}_2$ in the main text, the layer-dependent interaction potential $V_{ll'}(q)$ is computed by numerically solving the Poisson problem outlined above. The intralayer interaction [$l = l'$ in $V_{ll'}(q)$] is computed by taking $z = z' = z_{\text{Mo}}$, while the interlayer interaction [$l \neq l'$ in $V_{ll'}(q)$] is computed by taking $z = -z' = z_{\text{Mo}}$. These interactions in turn can also be used to compute the LLL Haldane pseudopotentials in Eq. (E5) with the effective magnetic length $\ell_B^* = 2.02\,\text{nm}$ appropriate for $t\text{MoTe}_2$ at $\theta = 3.7°$. Note that for $\boldsymbol{q} = 0$, the electric potential within the material slab is $\varphi_{i_m}(\boldsymbol{q} = 0, z) = \frac{Q|z - z'|}{2\epsilon_0 \epsilon_{i_m,\perp}}$.

a. Isotropic material embedded in isotropic dielectric substrate

In this subsection, we discuss the analytic form of the interaction potential for a simple configuration as presented in Ref. 60. We consider three slabs ($N_{\text{slab}} = 3$) and no metallic gates. The middle material slab $i_m = 1$ has width L, occupies $-L/2 \leq z \leq L/2$, and has isotropic dielectric constant ϵ_m. The rest of space consists of a background isotropic dielectric environment with dielectric constant ϵ_b. Define the ratio $\epsilon = \epsilon_m/\epsilon_b$. A free test charge Q is positioned at $\mathbf{r} = 0$ and $z = z'$. Using the formalism presented above, the solution to the electric potential in the material slab can be obtained analytically:

$$\varphi_1(q, z, z') = -\frac{Q}{2\epsilon_0\epsilon_m q}\left[e^{-q|z-z'|} + \frac{2\delta}{e^{2qL} - \delta^2}\Big(\delta\cosh(q(z-z')) + e^{qL}\cosh(q(z+z'))\Big)\right], \tag{E14}$$

where we defined $\delta = \frac{\epsilon-1}{\epsilon+1}$.

In the long-wavelength limit where $qL \ll 1$, the interaction becomes independent of z and z':

$$\varphi_1(q) = -\frac{Q}{2\epsilon_0\epsilon_m q}\frac{e^{qL} + \delta}{e^{qL} - \delta}. \tag{E15}$$

Finally, taking the limit as $\epsilon \gg 1$, we get the Rytova-Keldysh (RK) interaction

$$\varphi_1(q) = -\frac{Q}{2\epsilon_0\epsilon_b q(1 + l_{\text{RK}} q)}, \tag{E16}$$

where we defined the length scale $l_{\text{RK}} = \frac{\epsilon L}{2}$. For long distances corresponding to small q, the interaction is inversely proportional to ϵ_b as the electric field lines are predominantly in the background dielectric and outside of the material slab. For short distances (but still longer than L) corresponding to large q, the interaction is inversely proportional to ϵ_m as the electric field lines are mostly contained within the material slab. In the context of $t\text{MoTe}_2$ encapsulated by hBN, we have $\eta_{\text{MoTe}_2} \simeq 14.5$ and $\eta_{\text{hBN}} \simeq 5$ (see Sec. 4 B for a discussion of the anisotropic dielectric constants), from which we estimate $\epsilon = \eta_{\text{MoTe}_2}/\eta_{\text{hBN}} \simeq 2.9$. Combining this with $L \simeq 1.4\,\text{nm}$, we estimate $l_{\text{RK}} \simeq 2.0\,\text{nm}$.

If probing on distances much smaller than L, i.e. $qL \gg 1$, we find instead $\varphi_1(q) \simeq -Q/(2\epsilon_0\epsilon_m q)$. In the context of $t\text{MoTe}_2$ at $\theta \simeq 3.7°$, since the MoTe_2 slab thickness of $\simeq 1.4\,\text{nm}$ is appreciably smaller than the moiré lattice constant $\simeq 6\,\text{nm}$, this $qL \gg 1$ regime is not expected to be important for the low-energy physics.

b. Anisotropic material embedded in anisotropic dielectric substrate

The calculations in the previous subsection can be generalised to the more complicated three-slab set-up where an anisotropic material with dielectric constants $\epsilon_m^{\perp,\parallel}$ is embedded in an anisotropic dielectric background with $\epsilon_b^{\perp,\parallel}$. We define $\kappa_m = \sqrt{\frac{\epsilon_m^{\parallel}}{\epsilon_m^{\perp}}}$, $\kappa_b = \sqrt{\frac{\epsilon_b^{\parallel}}{\epsilon_b^{\perp}}}$ and $\epsilon = \frac{\epsilon_m^{\perp}}{\epsilon_b^{\perp}}$. The electric potential in the middle material slab can be obtained analytically:

$$\varphi(q, z, z') = -\frac{Q}{2\epsilon_0\eta_m q}\left(e^{-\kappa_m q|z-z'|} + \frac{N_- + e^{\kappa_m qL}N_+}{N_0}\right), \tag{E17}$$

where we defined

$$N_0 = \epsilon^2\kappa_m^2\left(e^{2\kappa_m qL} - 1\right) + 2\epsilon\kappa_b\kappa_m\left(e^{2\kappa_m qL} + 1\right) + \kappa_b^2\left(e^{2\kappa_m qL} - 1\right), \tag{E18}$$

$$N_\pm = (\epsilon\kappa_m - \kappa_b)e^{\kappa_m q(z\pm z')}\left[\epsilon\kappa_m(e^{\kappa_m q(2z'\mp L)} + 1) \mp \kappa_b(e^{\kappa_m q(2z'\mp L)} - 1)\right]. \tag{E19}$$

In the long-wavelength limit where $qL \ll 1$, and in the limit where $\epsilon \gg 1$, one recovers the RK interaction

$$\varphi(q) = -\frac{Q}{2\epsilon_0\eta_b q(1 + l'_{\text{RK}} q)}, \text{ where } l'_{\text{RK}} = \frac{1}{2}\epsilon L\frac{\kappa_m^2}{\kappa_b}. \tag{E20}$$

Note that compared to the isotropic case, the RK length is now multiplied by a $\frac{\kappa_m^2}{\kappa_b}$ factor. In the context of $t\text{MoTe}_2$ at $\theta \simeq 3.7°$, we have $\kappa_{\text{MoTe}_2} = 1.45$ and $\kappa_{\text{hBN}} = 1.40$ (see Sec. 4 B for a discussion of the anisotropic dielectric constants), from which we estimate $l_{\text{RK}} \simeq 3.0\,\text{nm}$.

B. Numerical results for V_0/V_1

In this section, we present numerical results for sample geometries relevant to $t\mathrm{MoTe}_2$, focusing on the impact of dielectric engineering in suppressing the pseudopotential ratio V_0/V_1. The material $t\mathrm{MoTe}_2$ slab is fixed to have thickness $w_{\mathrm{MoTe}_2} = 1.4\mathrm{nm}$ [96]. First principles calculations of untwisted bilayers [66] yield $\epsilon^{\parallel}_{\mathrm{MoTe}_2} = 21$ and $\epsilon^{\perp}_{\mathrm{MoTe}_2} = 10$, leading to $\eta_{\mathrm{MoTe}_2} = \sqrt{\epsilon^{\parallel}_{\mathrm{MoTe}_2}\epsilon^{\perp}_{\mathrm{MoTe}_2}} \simeq 14.5$. In some of the calculations below, we will treat η_{MoTe_2} as a tunable parameter to capture the uncertainty in the appropriate dielectric parameters for $t\mathrm{MoTe}_2$. The primary encapsulating substrate for $t\mathrm{MoTe}_2$ is hBN, which has $\epsilon^{\parallel}_{\mathrm{hBN}} = 6.9$ and $\epsilon^{\perp}_{\mathrm{hBN}} = 3.5$ [66]. Note that $\eta_{\mathrm{hBN}} = \sqrt{\epsilon^{\parallel}_{\mathrm{hBN}}\epsilon^{\perp}_{\mathrm{hBN}}} \simeq 5$. To soften the short-range repulsion and suppress the ratio V_0/V_1, we consider inserting one or more high-dielectric 'spacer' slabs on either side of the $t\mathrm{MoTe}_2$, with dielectric constants $\epsilon^{\perp,\parallel}_{\mathrm{spacer}}$.

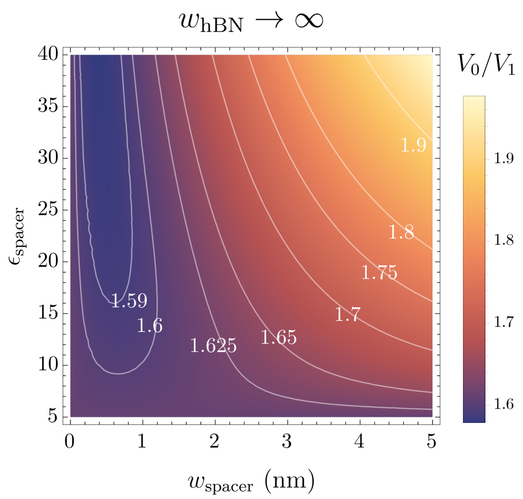

FIG. S16. **LLL pseudopotential ratio V_0/V_1 as a function of the spacer thickness and dielectric constant.** V_0/V_1 is minimized when $\epsilon_{\mathrm{spacer}}$ is large and $w_{\mathrm{spacer}} < 1\,\mathrm{nm}$, suggesting that a thin, high-dielectric material would be ideal. Interaction potential computed assuming $z = z' = 0$. We assume an isotropic spacer on both sides of the $t\mathrm{MoTe}_2$, and set the metallic gates infinitely far away. The white curves are contours of varying V_0/V_1.

In Fig. S16 we compute V_0/V_1 for the screened interaction when a spacer dielectric is added on either side of $t\mathrm{MoTe}_2$. In particular, we study the dependence of V_0/V_1 on the spacer width w_{spacer} and dielectric constant $\epsilon_{\mathrm{spacer}}$ (assumed isotropic). We also set $w_{\mathrm{hBN}} \to \infty$ and $z = z' = 0$ for simplicity. We see that V_0/V_1 is minimized when $w_{\mathrm{spacer}} \sim 0.5\mathrm{nm}$ roughly independent of $\epsilon_{\mathrm{spacer}}$. Moreover, V_0/V_1 for small w_{spacer} is roughly independent of $\epsilon_{\mathrm{spacer}}$, although it seems to reach slightly smaller values for larger $\epsilon_{\mathrm{spacer}}$. We note however that this behaviour does not persist for arbitrarily large $\epsilon_{\mathrm{spacer}}$, and for a sufficiently large dielectric constant the V_0/V_1 ratio will begin to increase again. Thus, the ideal set-up would be to use a thin material with moderately-high dielectric constant as a spacer. For concreteness, one example material is HfO_2 which is isotropic with $\epsilon_{\mathrm{HfO}_2} \simeq 26$ [71], though other dielectrics can be considered.

In Fig. S17, we plot V_0/V_1 as a function of w_{hBN} and w_{spacer} for different values of η_{MoTe_2}, fixing the dielectric anisotropy to $\kappa_{\mathrm{MoTe}_2} = 1.45$. For concreteness, we set $\epsilon^{\perp,\parallel}_{\mathrm{spacer}} = 26$ corresponding to HfO_2. We find that V_0/V_1 is overall lower for larger values of η_{MoTe_2}. For untwisted bilayer MoTe_2, we expect $\eta_{\mathrm{MoTe}_2} \approx 14.5$. However, screening coming from the moiré bands could enhance this value in $t\mathrm{MoTe}_2$. We also observe that larger w_{hBN}, and thus larger gate-to-gate distance, results in smaller values of V_0/V_1.

In the $w_{\mathrm{hBN}} \to \infty$ limit, the form of $V(q)$ is analytically tractable. Let $\kappa_1 = \kappa_{\mathrm{hBN}}, \kappa_2 = \kappa_{\mathrm{MoTe}_2}$, $\eta_1 = \eta_{\mathrm{hBN}}$, $\eta_2 = \eta_{\mathrm{MoTe}_2}$, $\epsilon_1 = \frac{\epsilon_{\mathrm{spacer}}}{\epsilon^{\perp}_{\mathrm{hBN}}}$, $\epsilon_2 = \frac{\epsilon^{\perp}_{\mathrm{MoTe}_2}}{\epsilon_{\mathrm{spacer}}}$. We also let $w_{\mathrm{MoTe}_2} = L$ and $w_{\mathrm{spacer}} = w$ in the expressions that follow for sake

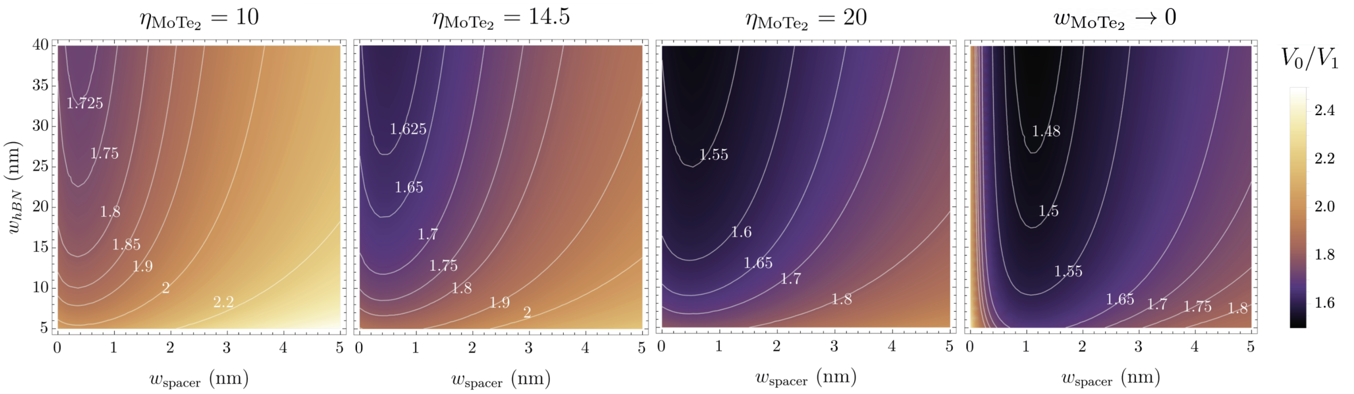

FIG. S17. **LLL pseudopotential ratio V_0/V_1 using a spacer with $\epsilon_{\mathrm{spacer}}^{\perp,\parallel} = 26$ on both sides of the tMoTe$_2$, as a function of w_{spacer} and w_{hBN}.** The results in the three panels on the left are plotted for varying values of $\eta_{\mathrm{MoTe_2}} = \sqrt{\epsilon^{\parallel}_{\mathrm{MoTe_2}}\epsilon^{\perp}_{\mathrm{MoTe_2}}}$, with a fixed $\kappa_{\mathrm{MoTe_2}} = 1.45$, showing that V_0/V_1 is smaller for larger values of $\eta_{\mathrm{MoTe_2}}$. From [66], we estimate $\eta_{\mathrm{MoTe_2}} \approx 14.5$ for untwisted bilayer MoTe$_2$, but this value could be enhanced due to screening coming from the moiré bands. The results in the right-most panel neglect the finite thickness of the tMoTe$_2$ and show lower values of V_0/V_1 compared to the left three panels. Interaction potential computed assuming $z = z' = 0$. The white curves are contours of varying V_0/V_1.

of brevity. Then we find:

$$\varphi(q) = -\frac{Q}{2\epsilon_0\eta_2 q}\left[\frac{2\left((\epsilon_2\kappa_2-1)\chi(q) - 4\epsilon_2\kappa_2\left(\kappa_1-\epsilon_1\right)e^{\kappa_2 qL}\right)}{\left((\epsilon_2\kappa_2+1)\,e^{\kappa_2 qL} - \epsilon_2\kappa_2+1\right)\chi(q)} + 1\right],$$
$$\chi(q) = \epsilon_2\kappa_1\kappa_2\left(e^{\kappa_2 qL}-1\right)\left(e^{2qw}-1\right) + \kappa_1\left(e^{\kappa_2 qL}+1\right)\left(e^{2qw}+1\right) + \epsilon_1\left[(\epsilon_2\kappa_2+1)\,e^{q(\kappa_2 L+2w)} + (1-\epsilon_2\kappa_2)\,e^{2qw} + (\epsilon_2\kappa_2-1)\,e^{\kappa_2 qL} - \epsilon_2\kappa_2 - 1\right]. \tag{E21}$$

This interaction can be expanded as a power series in q for $qL \ll 1$ and $qw \ll 1$, and taking terms up to $O(q)$ we find:

$$\varphi(q) \approx -\frac{Q}{2\epsilon_0\eta_1 q}\Bigg(1 - \underbrace{\frac{\epsilon_1^2\epsilon_2^2\kappa_2 L - 2(\kappa_1\epsilon_2 - \epsilon_1^2\epsilon_2)w - \kappa_1^2 L}{2\kappa_1\epsilon_1\epsilon_2}}_{l_{\mathrm{RK}}}\,q + o(q^2)\Bigg) \approx -\frac{Q}{2\epsilon_0\eta_1 q(1+l_{\mathrm{RK}}q)}, \tag{E22}$$

where we defined an effective RK length scale:

$$l_{\mathrm{RK}} = \frac{\epsilon_1^2\epsilon_2^2\kappa_2 L - 2(\kappa_1\epsilon_2 - \epsilon_1^2\epsilon_2)w - \kappa_1^2 L}{2\kappa_1\epsilon_1\epsilon_2} = \frac{1}{\kappa_1}\left(l'_{\mathrm{RK}}\kappa_2^2 + \left(\epsilon_1 - \frac{\kappa_1^2}{\epsilon_1}\right)w - \frac{\kappa_1^2}{2\epsilon_1\epsilon_2}L\right), \tag{E23}$$

where $l'_{\mathrm{RK}} = \epsilon_1\epsilon_2\frac{L}{2} = \frac{\epsilon^{\perp}_{\mathrm{MoTe_2}}}{\epsilon_{\mathrm{hBN}}}\frac{L}{2}$ is the RK length in the absence of a spacer. So for $w_{\mathrm{spacer}} \lesssim w_{\mathrm{MoTe_2}}$, the long wavelength interaction takes the form of an RK interaction with a renormalized length scale l'_{RK}. If $\kappa_1 < \epsilon_1$, then l'_{RK} will increase with w_{spacer} until roughly $w_{\mathrm{spacer}} \sim w_{\mathrm{MoTe_2}}$. On the other hand, when $w_{\mathrm{MoTe_2}} \to \infty$, a sensible approximation of the RK length should tend to $\epsilon_2\frac{L}{2}$ (this cannot be seen from Eq. (E23) which assumes $qw_{\mathrm{MoTe_2}} \ll 1$), which is smaller than l_{RK} with optimal w_{spacer}. Note that V_0/V_1 decreases as l_{RK} increases. Consequently, V_0/V_1 should be minimized for some value $w_{\mathrm{spacer}} \lesssim w_{\mathrm{MoTe_2}}$, and then increase again for large w_{spacer}. This explains why the optimal spacer thickness is bounded above by the tMoTe$_2$ thickness of $w_{\mathrm{MoTe_2}} = 1.4$nm. In Fig. S18 we compare the analytical solution to its RK asymptotic form, showing that the RK interaction is a good approximation at long wavelengths compared to the MoTe$_2$ thickness.

We note that the intrinsic finite thickness of MoTe$_2$ suppresses the impact of the dielectric spacer in decreasing V_0/V_1. This is clearly shown in the rightmost panel of Fig. S17, where lower values of V_0/V_1 can be obtained when MoTe$_2$ is treated as a 2D material with $w_{\mathrm{MoTe_2}} \to 0$ (this also ignores any contribution due to the in-plane polarizability [62]).

In Fig. S19, we account for the electrons residing on the Mo planes at $\pm z_{\mathrm{Mo}} = \pm 0.365$nm. We find that V_0/V_1 is reduced for the interlayer interaction, while the intralayer interaction is largely unaffected (this follows from the fact that the intralayer interaction is only weakly dependent on z in the long-wavelength limit, as discussed in Supplementary Note 4 A). In Fig. S20, we plot the V_0/V_1 ratios as a function of the spacer dielectric constant $\epsilon_{\mathrm{spacer}}$,

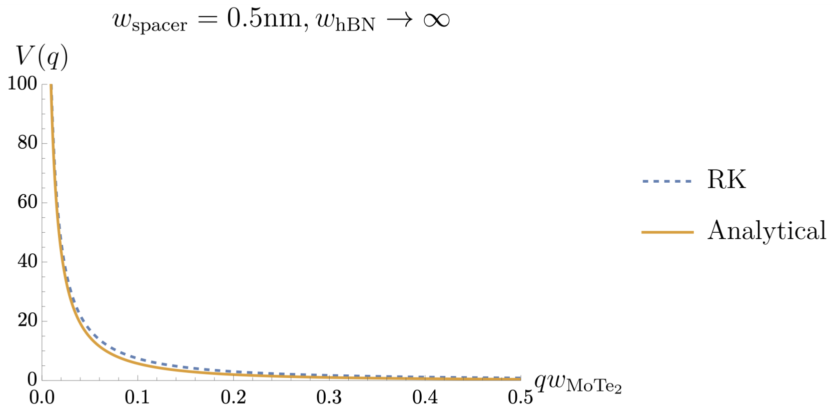

FIG. S18. **Comparison of the analytical form of the interaction for $w_{\text{hBN}} \to \infty$ and $\epsilon^{\perp,\parallel}_{\text{spacer}} = 26$ and its approximate asymptotic form as an RK interaction.** The RK interaction fits the analytical solution well for $qw_{\text{MoTe}_2} \ll 1$.

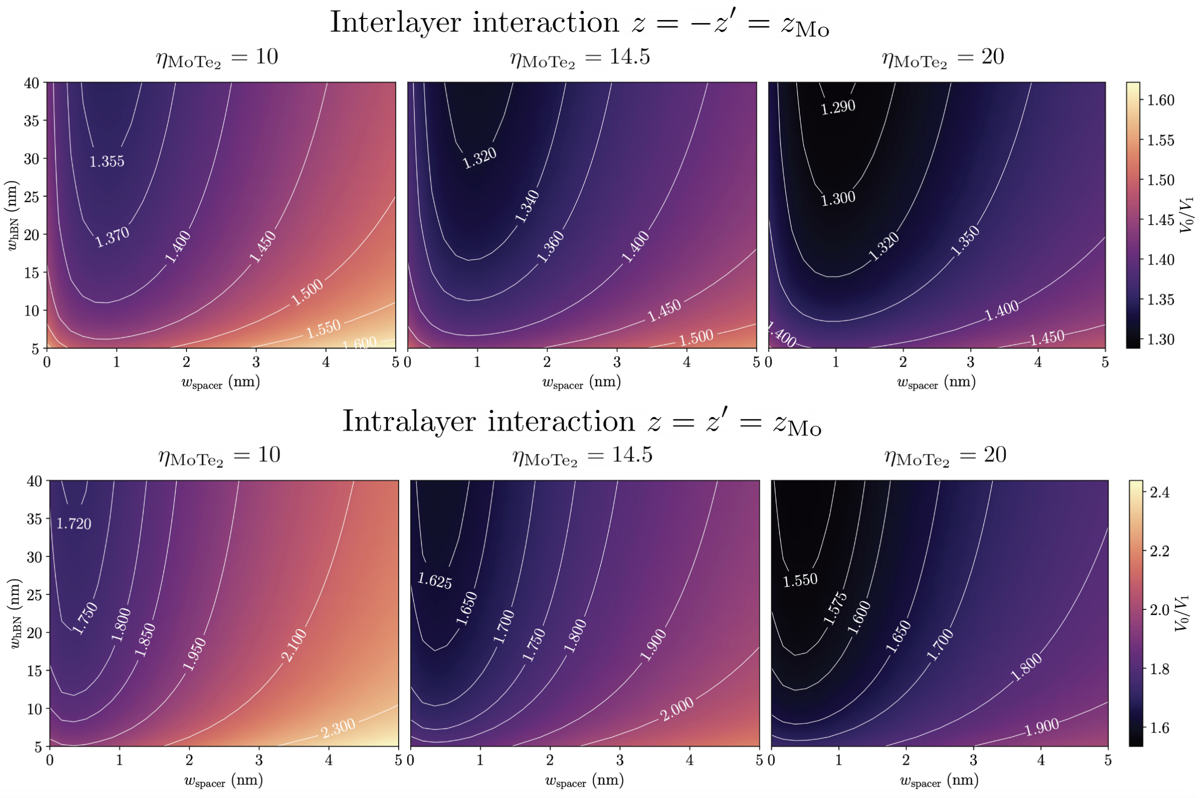

FIG. S19. **LLL pseudopotential ratio V_0/V_1 for the interlayer (top row) and intralayer (bottom row) interaction as a function of w_{spacer} and w_{hBN}.** The results are plotted for varying values of $\eta_{\text{MoTe}_2} = \sqrt{\epsilon^{\parallel}_{\text{MoTe}_2}\epsilon^{\perp}_{\text{MoTe}_2}}$, with a fixed $\kappa_{\text{MoTe}_2} = 1.45$, and assuming an isotropic spacer with $\epsilon^{\perp,\parallel}_{\text{spacer}} = 26$ placed on both sides of $t\text{MoTe}_2$. We set $z_{\text{Mo}} = \pm 0.365\,\text{nm}$ for the position of the Mo planes. Note that the colorbar ranges are different for the top and bottom row. The white curves are contours of varying V_0/V_1.

which exhibits a suppression for moderately high spacer dielectric constants, compared to the case without any spacers. V_0/V_1 increases again for sufficiently large ϵ_{spacer}, since the spacer acts like metallic gates for $\epsilon_{\text{spacer}} \to \infty$. V_0/V_1 decreases significantly for small $\epsilon_{\text{spacer}} < 1$ as the electric field lines are confined to the $t\text{MoTe}_2$, but such values of the spacer dielectric constant are not physically realistic.

We summarize the main messages of the calculations in this subsection. In the main text, our exact diagonalization calculations show that the FTI is stabilized in $\theta = 3.7^\circ$ $t\text{MoTe}_2$ when the intralyer interaction has $V_0/V_1 \simeq 1.1$ (see

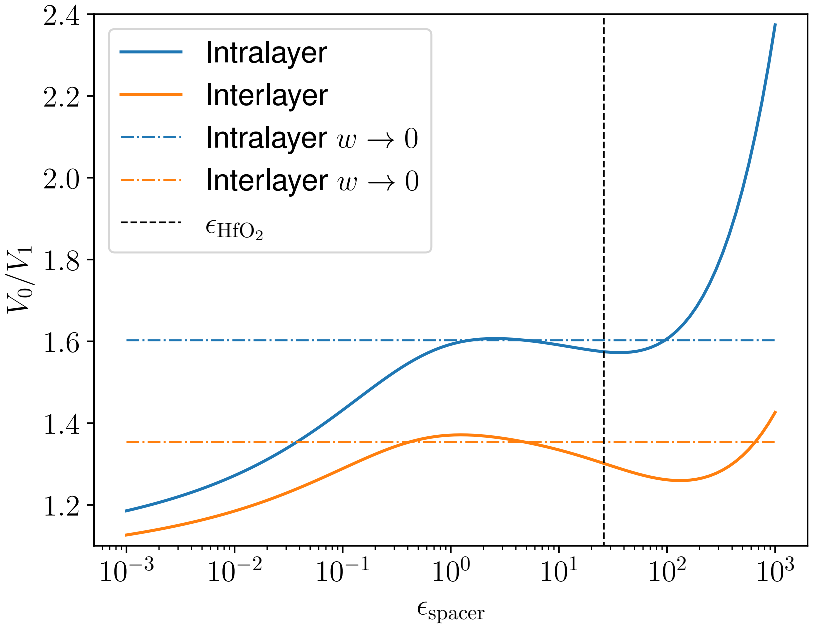

FIG. S20. V_0/V_1 **ratio for the intralayer (blue) and interlayer (orange) interaction as a function of the spacer dielectric constant.** The hBN slabs are taken to be infinitely thick, and the spacer dielectric is taken to be isotropic. We set $z_{\mathrm{Mo}} = \pm 0.365\,\mathrm{nm}$ for the position of the Mo planes, fix $w_{\mathrm{spacer}} = 0.5\,\mathrm{nm}$, and take $\epsilon^{\parallel}_{\mathrm{MoTe_2}} = 21, \epsilon^{\perp}_{\mathrm{MoTe_2}} = 10, \epsilon^{\parallel}_{\mathrm{hBN}} = 6.9$ and $\epsilon^{\perp}_{\mathrm{hBN}} = 3.5$. The horizontal blue and orange dot-dashed lines indicate the corresponding V_0/V_1 values in the absence of spacers $w_{\mathrm{spacer}} = 0$. Vertical black dashed line corresponds to $\epsilon_{\mathrm{spacer}}$ appropriate for $\mathrm{HfO_2}$.

e.g. Fig. 4b and c). Recall that the unscreened Coulomb interaction has $V_0/V_1 = 2$, which only increases in the presence of gate-screening (Fig. S4). By accounting for the finite thickness of the $\mathrm{MoTe_2}$ and using physically realistic values of the $\mathrm{MoTe_2}$ and hBN dielectric constants, we find that V_0/V_1 can be reduced to ~ 1.6 for a typical hBN thickness of $\simeq 30\,\mathrm{nm}$ (see bottom row of Fig. S19 for $\eta_{\mathrm{MoTe_2}} = 14.5$). While this represents a substantial reduction compared to the unscreened Coulomb interaction, this does not yet reach the regime of V_0/V_1 that we expect is required for the FTI. Introduction of spacer dielectric slabs can further reduce V_0/V_1. Considering $\epsilon^{\perp,\parallel}_{\mathrm{spacer}} = 26$, which is relevant for $\mathrm{HfO_2}$, we find that V_0/V_1 can be further reduced for a spacer width $w_{\mathrm{spacer}} \lesssim 0.5\,\mathrm{nm}$. However, at least for the parameters relevant to $\theta = 3.7°$ $t\mathrm{MoTe_2}$, we find that this reduction is small (Fig. S19).

N_s	N_1	N_2	f_{11}	f_{12}	f_{21}	f_{22}
9	3	3	1	0	0	1
9	9	1	1	2	1	1
12	3	4	1	0	0	1
12	3	4	1	-1	-1	2
15	15	1	1	3	0	1
18	18	1	1	-4	0	1

TABLE S1. **System sizes studied in this work.** N_s is the number of moiré unit cells, N_1 and N_2 are the numbers of points along the axes defined by the (tilted) reciprocal lattice vectors. f_{ij} are parameters describing the tilting of the lattice. In particular, the f_{ij} are the coordinates of new reciprocal lattice vectors $\mathbf{B}_{1,2}$ in terms of the standard basis reciprocal lattice vectors $\mathbf{b}_{1,2}$ (see Eq. (E2)). $f_{ij} = \delta_{ij}$ for an untilted lattice.

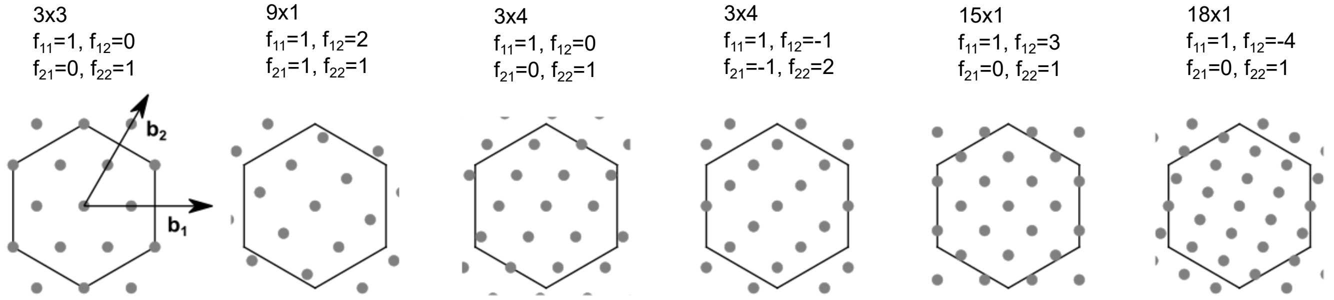

FIG. S21. **Momentum grids in the mBZ for different tilted finite-size lattices.** The lattices shown correspond to those listed in Tab. S1. The basis moiré reciprocal lattice vectors $\mathbf{b}_1$ and $\mathbf{b}_2$ are shown in the first panel.

Supplementary Note 5: Additional numerical data for tMoTe$_2$

In this appendix, we present further numerical results on tMoTe$_2$ beyond those presented in the main text, in particular with regards to different system sizes, dependence on other physical parameters, and band mixing. For simplicity, unless otherwise stated, the calculations in this section use the 2D gate-screened Coulomb interaction with a RK lengthscale l_{RK}

$$V_{ll'}(q) = \frac{e^2}{2\epsilon_0\epsilon q(1+l_{\mathrm{RK}}q)} \tanh\frac{q\xi}{2}, \tag{E1}$$

where ξ is the distance between the gates, and ϵ is the relative permittivity which is assumed isotropic. Note that Eq. E1 is independent of the layer indices l, l'. Based on non-moiré TMD studies [63, 64, 97–99], we estimate l_{RK} to be a few nm. The additional short-range interaction takes the form of Eq. (3) in the main text.

We list all the system sizes studied in this work in Tab. S1. In particular, we employ tilted lattices for different system sizes since, as known in finite-size studies of FCIs, lattice tilting can affect the results. We define the new reciprocal lattice vectors $\mathbf{B}_{1,2}$ as

$$\begin{aligned}\mathbf{B}_1 &= f_{11}\mathbf{b}_1 + f_{12}\mathbf{b}_2 \\ \mathbf{B}_2 &= f_{21}\mathbf{b}_1 + f_{22}\mathbf{b}_2,\end{aligned} \tag{E2}$$

where $\mathbf{b}_{1,2}$ are the standard basis moiré reciprocal lattice vectors (see Fig. S21, left), and the f_{ij} are integers. The set of inequivalent momenta that are not related by a reciprocal lattice vector can be generated as $n_1\mathbf{B}_1/N_1 + n_2\mathbf{B}_2/N_2$, with $n_1 = 0, \ldots, N_1 - 1$ and $n_2 = 0, \ldots, N_2 - 1$. The momentum grids in the mBZ are shown in Fig. S21.

enhanced

A. Effects of system parameters

In this subsection, we consider the properties of the FTI phase, and how it is impacted by changing various system parameters.

We compare the $\lambda - g$ phase diagram for four different system sizes in Fig. S22, which shows that larger system sizes tend to favor the FTI state. There are also variations for fixed N_s depending on the lattice tilting (compare the untilted and tilted 3×4 lattices). The results in Fig. S22 are for the bare gate-screened Coulomb interaction ($l_{\mathrm{RK}} = 0$nm), since the optimal RK length depends on the system size. We observe that for $g = 0$, the FTI survives for only a small range of $\lambda \lesssim 0.2$ (recall that λ is a parameter that multiplies the intervalley part of the interaction, see Eq. (E4)), but the stability window can be substantially enhanced for finite g. In Fig. S25 we show the same results but for $l_{\mathrm{RK}} = 3$nm. Note the difference in the g-axis scale compared to Fig. S22: in the presence of finite l_{RK}, the magnitude of $|g|$ required to obtain the FTI is reduced. The FTI phase for the 15×1 lattice persists to $\lambda = 1$. Note also the adiabatic continuity of the gapped phase to $\lambda = g = 0$, where the ground state in the spin-unpolarized sector is a decoupled product of two FCIs.

In Figs. S28 and S29 we show the dependence of the FTI stability on the displacement field D and the gate-screening length ξ respectively. The displacement field is deleterious to the FTI, and we find that the spacing/gap ratio improves for larger ξ. In Fig. S30, we show s_9/Δ_9 as a function of l_{RK} and g for $\xi = 20$ and 2000 nm. The spacing/gap ratio is minimal approximately along a stripe of constant V_0/V_1. The FTI does not survive for much larger or smaller l_{RK} than those shown in Fig. S30, implying that the higher pseudopotentials $V_{m>1}$ still play a non-trivial role in the stability of the FTI. The results for $\xi = 20$ and 2000 nm are very similar, demonstrating that $\xi = 20$ nm already yields results close to the limit $\xi \to \infty$.

In Fig. S31 we show that the momentum space occupation number fluctuations σ_{n_k} of the FTI correlate with the spacing/gap ratio s_9/Δ_9. In particular, a smaller σ_{n_k} is correlated with a smaller s_9/Δ_9.

B. Spin depolarization

A key criterion for obtaining a time-reversal symmetric FTI at $\nu = -4/3$ is that the global ground state of the system has zero spin polarization at this filling. (Other FTIs with topological order but not necessarily edge states can be obtained if time reversal symmetry is broken.) In this subsection, we provide numerical results pertaining to the spin polarization as a function of system parameters and band mixing.

In Fig. S32 we show how band mixing affects the ground state energies across different spin sectors for the untilted 3×3 lattice. The total energies in every S_z sector are converged at $N^1_{\mathrm{max}} = 7$. In Fig. S33 we show that the band mixing can lead to complete depolarization of the spin of the global ground state for the larger untilted 3×4 lattice. In Fig. S34 we show that large, negative g as well as finite l_{RK} have a similar depolarizing effect on the ground state.

For the tilted 3×4 and tilted 9×1 lattices, Figs. S24 and S23 (S27 and S26) show that the entire FTI phase in Fig. S22 for $l_{\mathrm{RK}} = 0$ nm (Fig. S25 for $l_{\mathrm{RK}} = 3$ nm) is indeed the global ground state across all magnetization sectors.

C. Insights from Hartree-Fock

We expect that a narrow bandwidth is an important requirement for stabilizing an FTI phase. For instance, Fig. S31 demonstrates that a small FTI spacing/gap ratio is correlated with the FTI having small number occupation fluctuations across the mBZ. A significant dispersion would penalize having such a homogeneous number occupation distribution. However, the non-interacting bandwidth is not necessarily a reliable proxy for judging the effective dispersion, since interaction effects may significantly renormalize the bandwidth. In this subsection, we discuss our estimations of the Hartree-renormalized bandwidth and its correlation with the continuum model parameters and the FTI phase. We also comment on the fate of the $|C| = 1$ insulator at $\nu = -1$ within Hartree-Fock calculations.

In Figs. S35 and S36, we show that the minimum bandwidth W_{Hartree} of the Hartree-renormalized band structure gives a good estimate of the region where the FTI has the lowest value of s_9/Δ_9. To obtain the Hartree-renormalized band structure in a 1BPV model, we consider the Hartree potential generated by the momentum-independent one-body hole density matrix $P_0 = \frac{2}{3}\mathbb{1}$, where $\mathbb{1}$ is the identity in valley space. Hence, P_0 corresponds to a state at physical filling $\nu = -4/3$. The Hartree-renormalized band structure is given by the sum of the non-interacting dispersion and the Hartree potential. The choice of P_0 is motivated from the fact that the occupation number in the FTI is mostly uniform across the mBZ (Fig. S31).

In Fig. S37 we show properties of the band structure as a function of the continuum model parameters. We note that for parameters where the Chern number of the highest single-particle valence band is non-zero, the region where the Hartree-renormalized bandwidth W_{Hartree} is minimal correlates with the region where the Berry curvature fluctuations are small.

In Fig. S38, we repeat the calculation of Fig. 3c in the main text for smaller values of $|g|$. We find that for certain continuum model parameters with reduced $|w/V|$, the FTI can survive down to $g \sim -900$ meVnm^2.

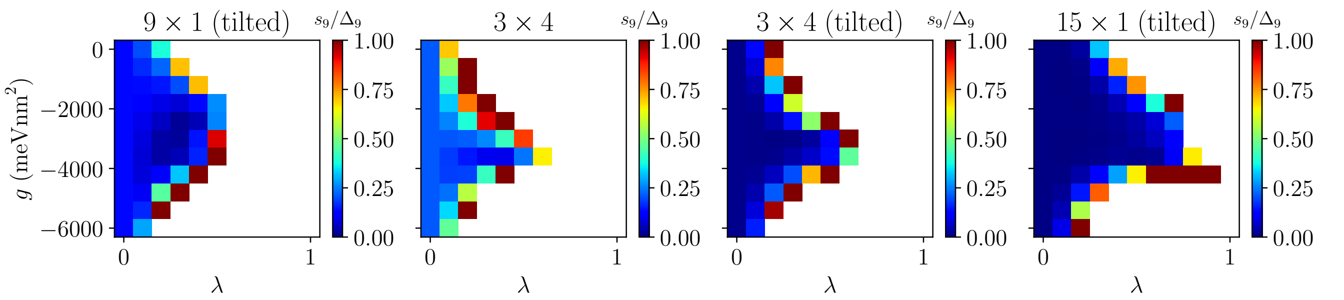

FIG. S22. **FTI stability region for different system sizes.** For four different lattices, we plot the spacing/gap ratio s_9/Δ_9 where $\Delta_9 > 0$ (white regions correspond to where $\Delta_9 < 0$). The FTI stability region is largest for the tilted 15×1 lattice (which is the lattice that is primarily used in the main text). We choose $10/\epsilon = 2$ and $l_{\mathrm{RK}} = 0\,\mathrm{nm}$.

In Fig. S39, we repeat the $N_s = 15$ 1BPV calculations of Figs. 3b,d and Fig. 4b, except that we project into band 1 instead of band 0. We find that the FTI can have a much lower s_9/Δ_9 ratio, and survives to significantly lower values of $|g|$. Furthermore, the FTI phase extends over a larger range of kinetic factor κ_{SP}, including the flat-band limit $\kappa_{\mathrm{SP}} = 0$. The FTI similarly drifts to smaller $|g|$ when the dielectric constant of the $\mathrm{MoTe_2}$ slab is increased.

In Fig. S40, we perform self-consistent Hartree-Fock calculations at $\nu = -1$, which allow for intervalley coherence at wavevectors $\boldsymbol{q} = \Gamma_{\mathrm{M}}, K_{\mathrm{M}}, K'_{\mathrm{M}}$. Note that the interaction we use here differs from previous theoretical HF studies of $t\mathrm{MoTe_2}$. In particular, we use the layer-dependent interaction obtained by solving the Poisson equation (see Supplementary Note 4 for details) for the configuration in Fig. 4a with $\epsilon^{\perp}_{\mathrm{MoTe_2}} = 10$, $\epsilon^{\parallel}_{\mathrm{MoTe_2}} = 21$ and $\epsilon^{\perp,\parallel}_{\mathrm{spacer}} = 26$. We project onto both band 0 and band 1 (i.e. a 2BPV calculation) — a 1BPV calculation within band 0 would yield valley polarized Chern insulators for all the parameters shown. In the 2BPV case, we find that HF yields a $|C| = 1$ Chern insulator at $\nu = -1$ for the parameters where 1BPV ED gives an FTI at $\nu = -4/3$ (see Fig. 4c in the main text). However, the HF state is only partially valley-polarized and has a finite amount of intervalley coherence.

D. Fractional Chern insulators at $\nu = -2/3$

The spin-polarized FCI phase at $\nu = -2/3$ has been extensively studied in previous works [13–21]. However, the interaction that we use (see Supplementary Note 4 for more details) for calculations of the FTI phase at $\nu = -4/3$ in the main text differs in several respects from these previous works. Firstly, we account for the layer-dependence of the interaction by considering the continuum model degrees of freedom to reside on the Mo planes at $z = \pm 0.365\,\mathrm{nm}$. Secondly, our interaction potential is computed with the influence of the (anisotropic) dielectric environment imposed by the $t\mathrm{MoTe_2}$ device geometry. This subsection addresses how these factors affect the FCI phase at $\nu = -2/3$.

In Fig. S41, we perform spin-polarized ED calculations at $\nu = -2/3$ on the 3×4 untilted lattice, using different interaction potentials. We project into band 0 and band 1, with no restriction on the band occupations (i.e. 2BPV calculations). We present phase diagrams as a function of twist angle θ and a multiplicative factor $10/\epsilon$ or u that scales the overall strength of the interaction. Note that here, we consider the calculation to yield an FCI if the spread of the FCI ground state manifold is less than the gap to higher energy states, since this measure has been widely used in previous studies of the FCI at $\nu = -2/3$. In Fig. S41a,b, we consider a dielectric environment that is used in most theoretical treatments of $t\mathrm{MoTe_2}$, i.e. we assume a uniform isotropic dielectric with dielectric constant ϵ which is terminated by perfect metallic gates at $z = \pm\xi/2$, where we choose $\xi = 20\,\mathrm{nm}$. In Fig. S41a, we place the continuum model layers at $z = 0$, yielding a layer-independent interaction $V(q) \propto \tanh(q\xi/2)/\epsilon q$. In Fig. S41b, we place the continuum model layers at the Mo planes at $z = \pm 0.365\,\mathrm{nm}$, leading to a layer-dependent interaction. In Fig. S41c,d, the interaction is obtained by solving the Poisson equation for the configuration in Fig. 4a with $\epsilon^{\perp}_{\mathrm{MoTe_2}} = 10, \epsilon^{\parallel}_{\mathrm{MoTe_2}} = 21$ and no spacer. In Fig. S41c, the continuum model layers are at $|z| = 0\,\mathrm{nm}$, while in Fig. S41d, the continuum model layers are at $|z| = \pm 0.365\,\mathrm{nm}$. In all cases, we find that the FCI phase lies in a stripe-like region in the phase space of interaction strength and twist angle. This implies that the properties of the FCI are qualitatively unchanged by incorporating the layer-dependence of the interaction and finer details of the dielectric screening.

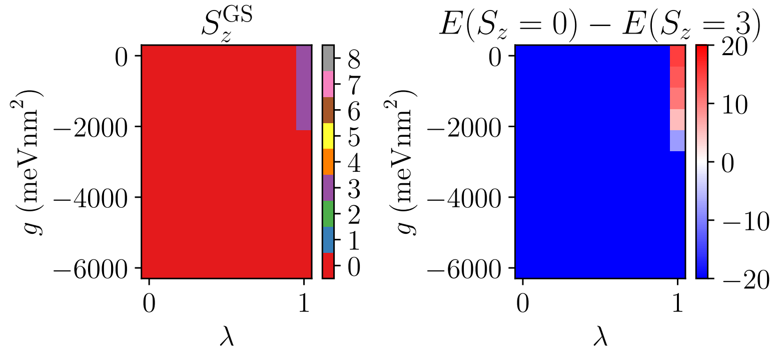

FIG. S23. **Spin polarization for the tilted 9×1 lattice.** Left: Spin polarization of the ground state. Right: Energy difference in meV between the ground state in the spin unpolarized sector and in the maximally polarized sector. We choose $10/\epsilon = 2$ and $l_{\rm RK} = 0\,$nm.

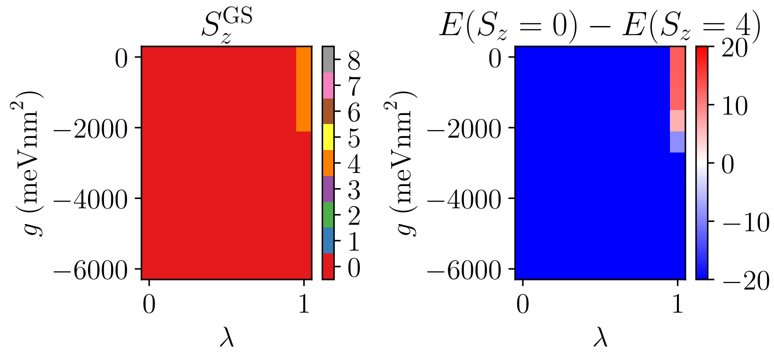

FIG. S24. **Spin polarization for the tilted 3×4 lattice.** Left: Spin polarization of the ground state. Right: Energy difference in meV between the ground state in the spin unpolarized sector and in the maximally polarized sector. We choose $10/\epsilon = 2$ and $l_{\rm RK} = 0\,$nm.

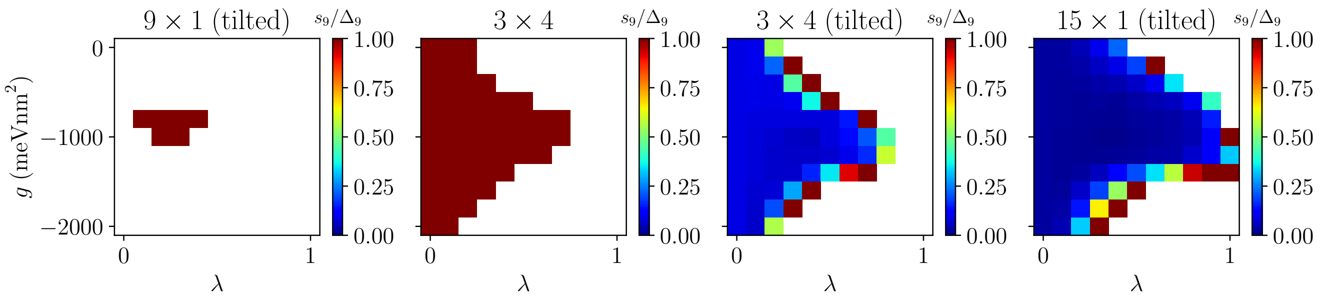

FIG. S25. **FTI stability region for different system sizes with finite $l_{\rm RK}$.** For four different lattices, we plot the spacing/gap ratio s_9/Δ_9 where $\Delta_9 > 0$ (white regions correspond to where $\Delta_9 < 0$). The FTI stability region is largest for the tilted 15×1 lattice (which is the lattice that is primarily used in the main text). We choose $10/\epsilon = 2$ and $l_{\rm RK} = 3$nm.

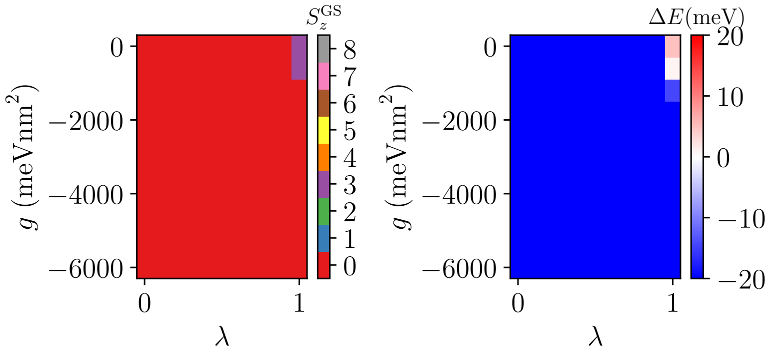

FIG. S26. **Spin polarization with finite $l_{\mathbf{RK}}$ for the tilted** 9×1 **lattice.** Left: Spin polarization of the ground state. Right: Energy difference between the ground state in the spin unpolarized sector and in the maximally polarized sector: $\Delta E = E(S_z = 0) - E(S_z = 3)$. We choose $10/\epsilon = 2$ and $l_{\mathrm{RK}} = 3\,\mathrm{nm}$.

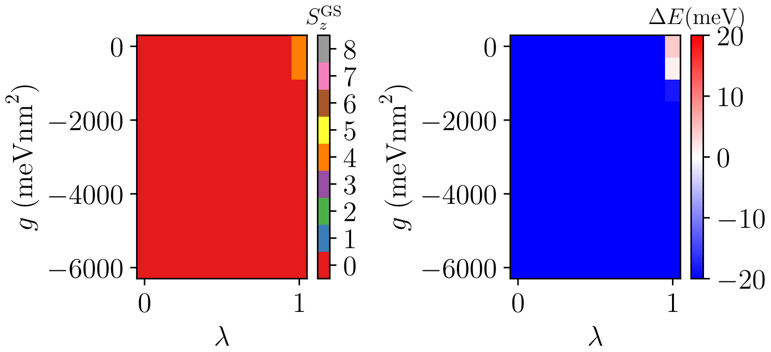

FIG. S27. **Spin polarization with finite $l_{\mathbf{RK}}$ for the tilted** 3×4 **lattice.** Left: Spin polarization of the ground state. Right: Energy difference between the ground state in the spin unpolarized sector and in the maximally polarized sector: $\Delta E = E(S_z = 0) - E(S_z = 4)$. We choose $10/\epsilon = 2$ and $l_{\mathrm{RK}} = 3\,\mathrm{nm}$.

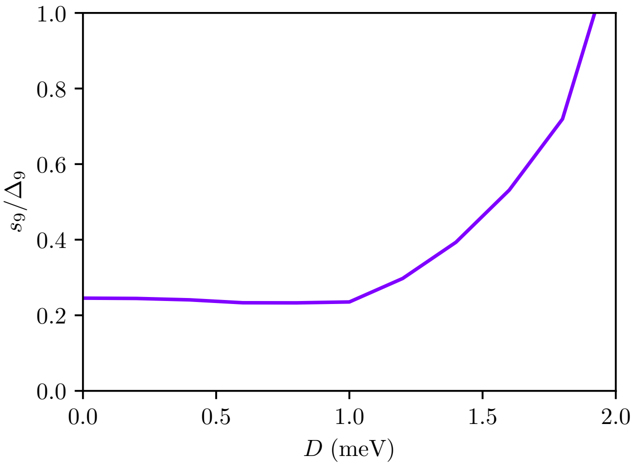

FIG. S28. **Stability of FTI under displacement field.** For the tilted 15×1 lattice we plot the FTI spacing/gap ratio under an applied displacement field D. Applying a displacement field destroys the FTI. We choose $10/\epsilon = 2$ and $l_{\mathrm{RK}} = 3\mathrm{nm}$ and $g = -1250\mathrm{meVnm}^2$.

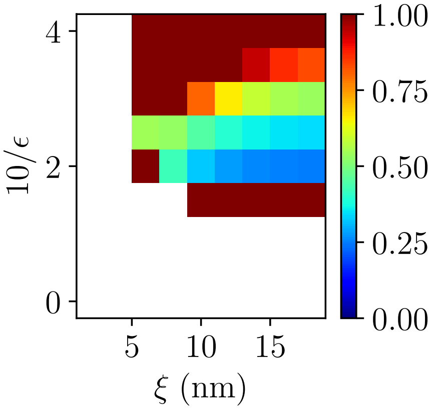

FIG. S29. **Dependence on screening length.** We plot s_9/Δ_9 for the tilted 15×1 lattice at $\theta = 3.7°$. Smaller values of the screening length ξ are worse for the FTI. White regions correspond to where $\Delta_9 < 0$. Parameters: $\theta = 3.7°$, $(\epsilon/10) \times g = -625\text{meVnm}^2$ and $l_{\rm RK} = 3\text{nm}$.

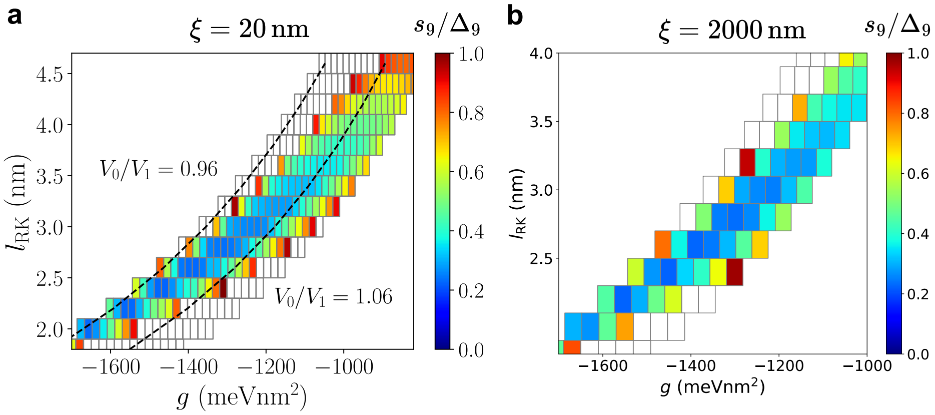

FIG. S30. **Dependence on $l_{\rm RK}$ and g for different screening lengths.** White regions correspond to where $\Delta_9 < 0$ or $s_9/\Delta_9 > 1$. a) $\xi = 20\,\text{nm}$. Contours of constant V_0/V_1 are indicated. b) $\xi = 2000\,\text{nm}$. In both cases, calculations are performed on the tilted 15×1 lattice ($N_s = 15$) with $\theta = 3.7°$ and $\epsilon = 5$.

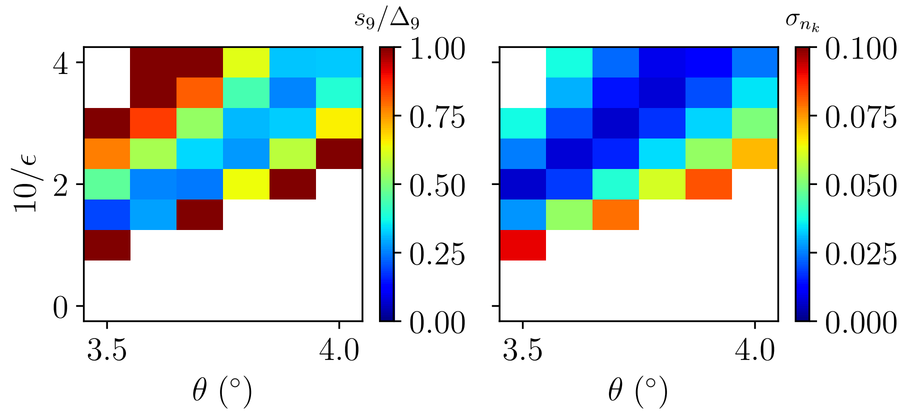

FIG. S31. **Occupation number fluctuations.** For the tilted 15×1 lattice we plot the FTI spacing/gap ratio (left; white regions correspond to where $\Delta_9 < 0$) and compare to the occupation number fluctuations σ_{n_k} (right) as a function of interaction strength $10/\epsilon$ and twist angle θ. The FTI spacing/gap ratio is smallest where the fluctuations in the occupation number are smallest. We choose $l_{\mathrm{RK}} = 3\mathrm{nm}$ and $(\epsilon/10) \times g = -625\mathrm{meVnm}^2$.

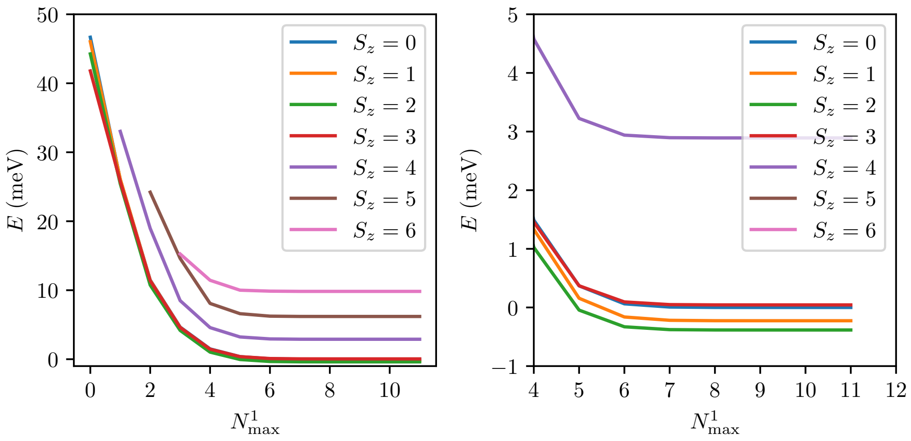

FIG. S32. **Effect of band mixing on ground state energy across different spin sectors for the 3×3 lattice.** The ground state energies in the different spin sectors are plotted as a function of the maximum number of holes N^1_{max} allowed in band 1 according to our truncation scheme (see main text). The energies converge as N^1_{max} is increased. The right plot is a zoomed-in version of the left. We choose $10/\epsilon = 0.6$, $g = 0\mathrm{meVnm}^2$ and $l_{\mathrm{RK}} = 0\mathrm{nm}$.

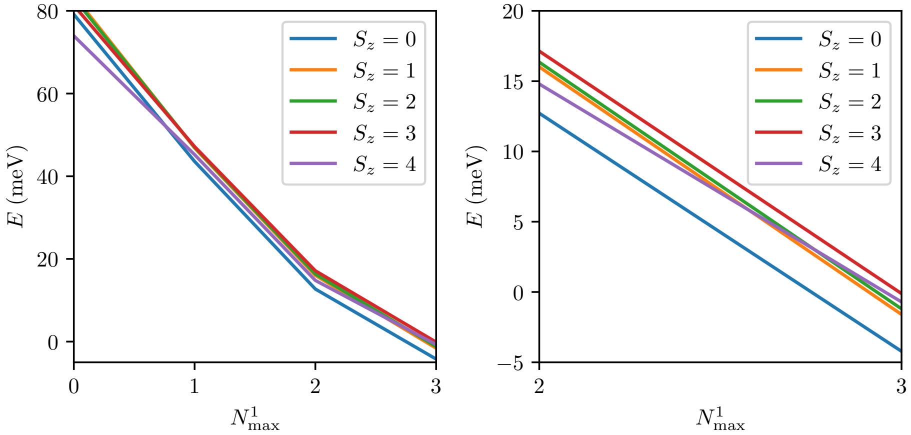

FIG. S33. **Spin depolarization from band mixing for the** 3×4 **lattice**. Same as Fig. S32 but for the 3×4 lattice. As the allowed number of holes $N^1_{\rm max}$ in the remote band is increased, the ground state changes from a spin-polarized state to a spin-unpolarized state. Since the FTI state occurs in the spin-unpolarized sector, this shows that a necessary (but not sufficient) condition for the FTI to emerge for this particular choice of parameters ($10/\epsilon = 0.6$, $g = 0$meVnm^2 and $l_{\rm RK} = 0$nm) is band mixing. The right plot is a zoomed-in version of the left.

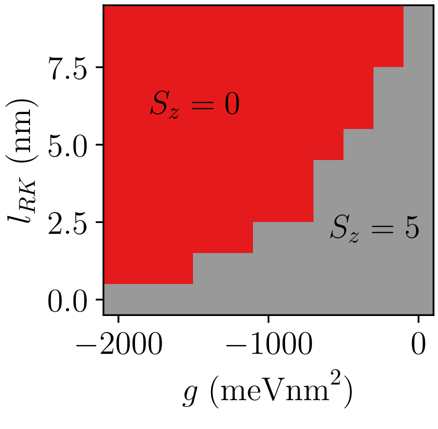

FIG. S34. **Spin depolarization with** g **and** $l_{\rm RK}$. We choose $10/\epsilon = 2$ and study the 15×1 tilted lattice with $N^1_{\rm max} = 0$. Both g and $l_{\rm RK}$ have a depolarizing effect on the ground state.

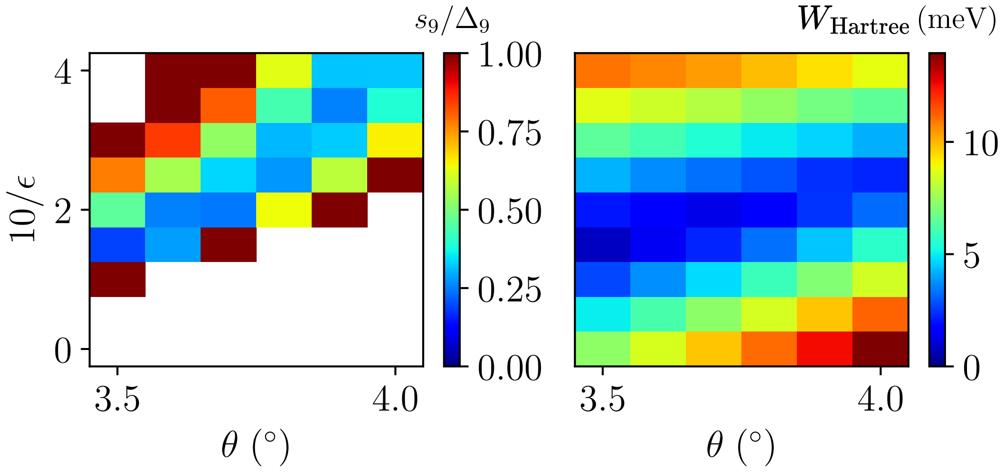

FIG. S35. **Comparison of $\theta-(10/\epsilon)$ phase diagram with Hartree renormalized bandstructure**. Left: FTI spacing/gap ratio as a function of $10/\epsilon$ and θ. White regions correspond to where $\Delta_9 < 0$. Right: Bandwidth W_{Hartree} of the Hartree renormalized bandstructure. We perform a single-shot (i.e. not self-consistent) Hartree calculation for the tilted 15×1 lattice with the hole density matrix $\nu_s \mathbb{1}$ where the average hole occupation per spin is $\nu_s = 2/3$. All the parameters in the Hartree calculation are the same as the ED $[(\epsilon/10) \times g = -625\text{meVnm}^2$, $l_{\text{RK}} = 3\text{nm}]$.

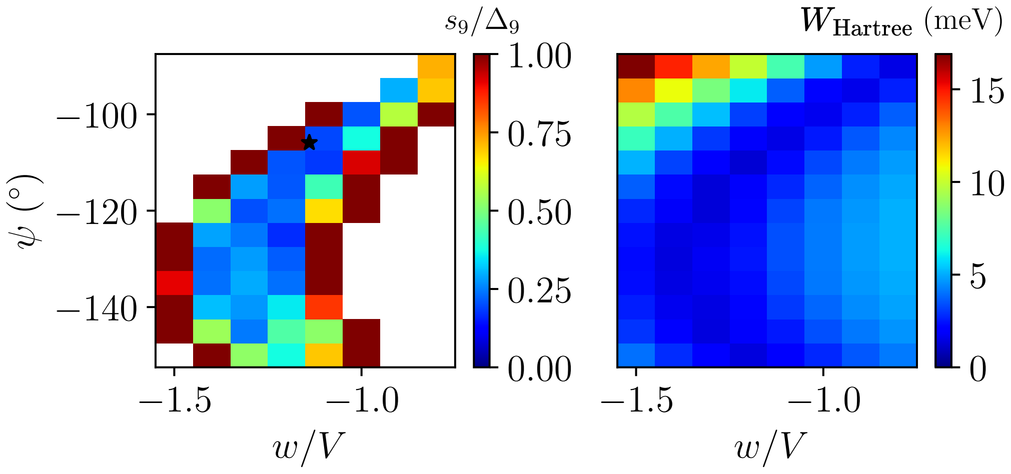

FIG. S36. **Comparison of $w/V-\psi$ phase diagram with Hartree renormalized bandstructure**. Left: FTI spacing/gap ratio as a function of continuum model parameters. White regions correspond to where $\Delta_9 < 0$. The black star indicates the continuum model parameters of Ref. [22]. Right: Bandwidth W_{Hartree} of the Hartree renormalized bandstructure. We perform a single-shot (i.e. not self-consistent) Hartree calculation for the tilted 15×1 lattice with the hole density matrix $\nu_s \mathbb{1}$, where the average hole occupation per spin is $\nu_s = 2/3$. All the parameters in the Hartree calculation are the same as the ED (at fixed $\sqrt{w^2+V^2} = 25\text{meV}$, $g = -1250\text{meVnm}^2$, $l_{\text{RK}} = 3\text{nm}$, $\theta = 3.7°$, $10/\epsilon = 2$).

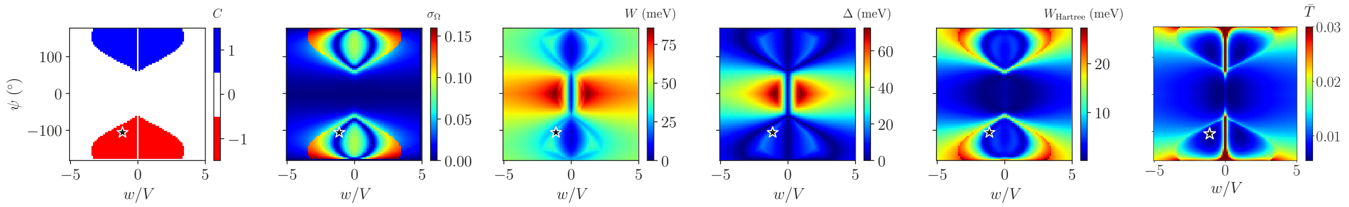

FIG. S37. **Bandstructure properties of the highest valence band in the $w/V - \psi$ phase diagram**. From left to right, we show the Chern number C, Berry curvature fluctuations σ_Ω, single-particle bandwidth W, bandgap Δ, Hartree-renormalized bandwidth $W_{\rm Hartree}$ and trace condition violation $\bar{T}$ (at fixed $\sqrt{w^2+V^2} = 25$meV and $\theta = 3.7°$). W is calculated as the combined bandwidth of the highest and second highest valence band. Similarly Δ is the direct gap between the highest and second highest valence band. $W_{\rm Hartree}$ is calculated from a single-shot (i.e. not self-consistent) Hartree calculation for the tilted 15×1 lattice with density matrix $\nu_s \mathbb{1}$, where the average occupation per spin is $\nu_s = 2/3$. For the Hartree calculation we use a gate-screened Coulomb interaction with $10/\epsilon = 2$, $g = -1250$meVnm^2 and $l_{\rm RK} = 3$nm. The black star indicates the continuum model parameters of Ref. [22].

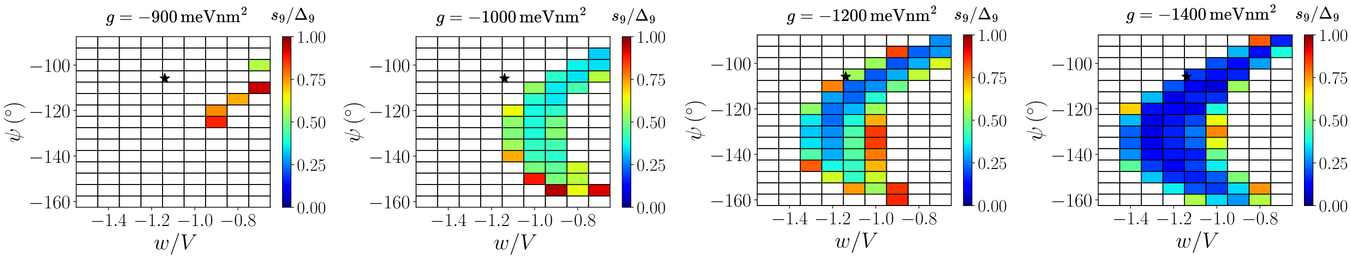

FIG. S38. **Varying continuum model parameters for different g.** White regions correspond to where $\Delta_9 < 0$ or $s_9/\Delta_9 > 1$. From left to right, we show the analog of Fig. 3c in the main text for $g = -900, -1000, -1200, -1400$ meVnm^2, on the tilted 15×1 lattice. Note that we use the layer-dependent interaction obtained by solving the Poisson equation for the configuration in Fig. 4a with $\epsilon^{\perp}_{\rm MoTe_2} = 10$, $\epsilon^{\parallel}_{\rm MoTe_2} = 21$ and no spacer.

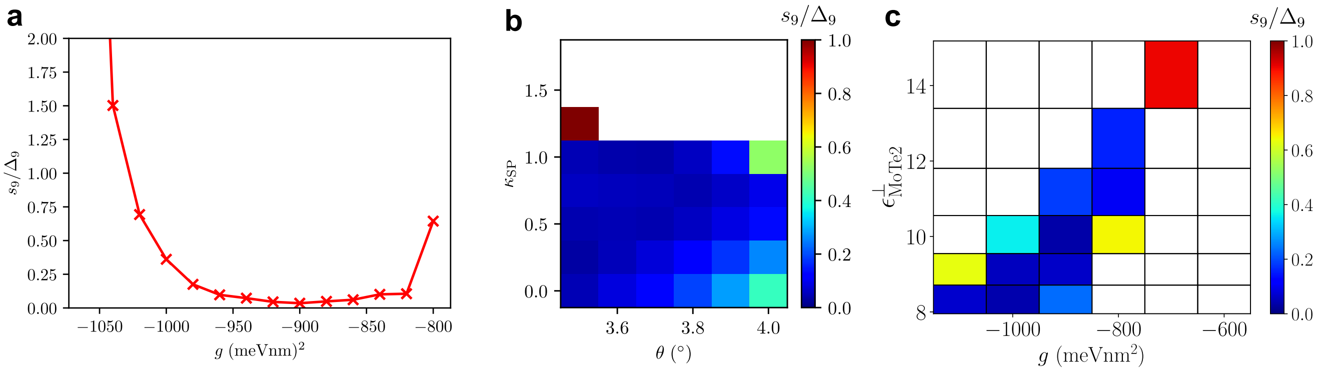

FIG. S39. **Projection into band 1.** ED calculations are performed on the tilted 15×1 lattice. Note that for all three plots, we use the layer-dependent interaction obtained by solving the Poisson equation for the configuration in Fig. 4a with no spacer. a) FTI spacing/gap ratio as a function of g. We set $\epsilon^{\perp}_{\rm MoTe_2} = 10$ and $\epsilon^{\parallel}_{\rm MoTe_2} = 21$. b) s_9/Δ_9 as a function of twist angle θ and kinetic energy factor $\kappa_{\rm SP}$ for $g = -900$ meVnm^2. White regions correspond to where $\Delta_9 < 0$. We set $\epsilon^{\perp}_{\rm MoTe_2} = 10$ and $\epsilon^{\parallel}_{\rm MoTe_2} = 21$. c) s_9/Δ_9 as a function of $\epsilon^{\perp}_{\rm MoTe_2}$ and g for fixed $\epsilon^{\parallel}_{\rm MoTe_2}/\epsilon^{\perp}_{\rm MoTe_2} = 2.1$.

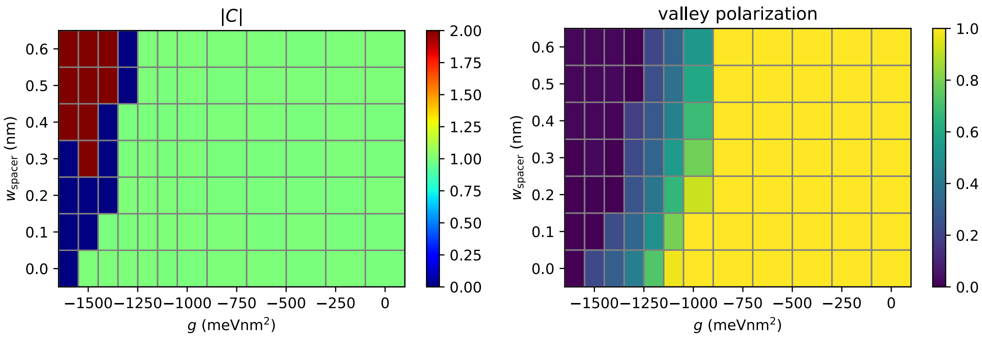

FIG. S40. **Self-consistent Hartree-Fock calculations at $\nu = -1$.** Left: Chern number C. Right: Valley polarization. System size is 18×18, and 2 bands kept per valley. Note that we use the layer-dependent interaction obtained by solving the Poisson equation for the configuration in Fig. 4a with $\epsilon^{\perp}_{\rm MoTe_2} = 10$, $\epsilon^{\parallel}_{\rm MoTe_2} = 21$ and $\epsilon^{\perp,\parallel}_{\rm spacer} = 26$.

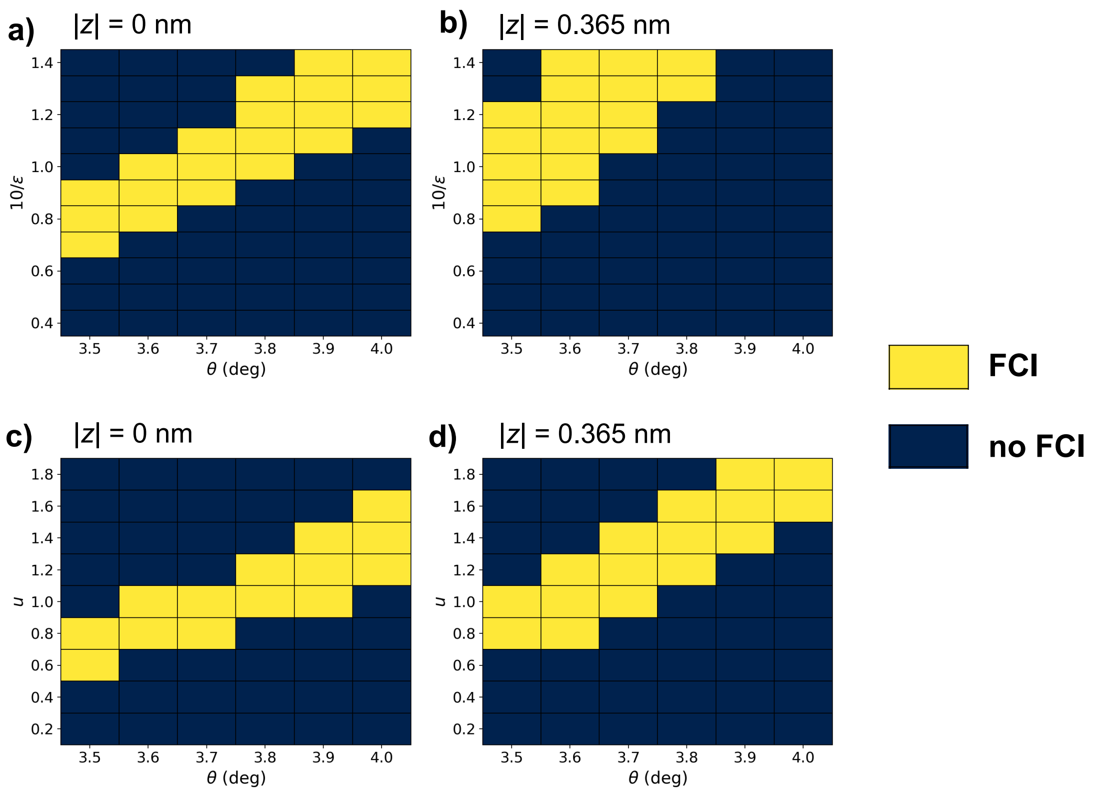

FIG. S41. **ED calculations of FCIs at $\nu = -2/3$.** All calculations are performed on the untilted 3×4 lattice, and projected into band 0 and band 1 (see Fig 1a), with no restriction on the band occupations (i.e. 2BPV calculations). We label a region an FCI if the spread of the FCI ground manifold is less than the gap to higher energy states. a,b) Interaction is computed by assuming an isotropic dielectric with dielectric constant ϵ. The metallic gates are at $z = \pm 10\,$nm. In a), the continuum model layers are at $|z| = 0\,$nm, while in b), the continuum model layers are at $|z| = \pm 0.365\,$nm. c,d) The interaction is obtained by solving the Poisson equation for the configuration in Fig. 4a with $\epsilon^{\perp}_{\rm MoTe_2} = 10$, $\epsilon^{\parallel}_{\rm MoTe_2} = 21$ and no spacer. u is a multiplicative factor that scales the overall strength of the interaction. In c), the continuum model layers are at $|z| = 0\,$nm, while in d), the continuum model layers are at $|z| = \pm 0.365\,$nm.

Supplementary Note 6: Edge mode stability and transport

A strong experimental indication for the realization of a topologically ordered state in FQHE and FCI phases is the fractionally quantized charge or thermal Hall response. For FTIs topological transport (edge) responses may or may not be available depending on the symmetries of the system – irrespective of whether they support topological anyonic excitations in the 2D bulk. Here, we discuss the $\nu = -4/3$ FTI from this perspective. First in Supplementary Note 6 A, we clarify that topological edge modes here are only stable when spin U(1) symmetry is present along with TR symmetry and charge U(1). This is in contrast to e.g. the FTI at filling factor $\nu = 1/3 + 1/3$ whose edge modes are stable against perturbations that violate spin U(1) symmetry [35]. Subsequently in Supplementary Note 6 B, we list the fractionally quantized charge transport responses of a four-contact geometry, assuming all these symmetries are present. We choose units where $e = h = 1$ for simplicity.

A. Stability analysis

The stability of the edge modes in FTIs has been studied in Ref. 36 and we use the notation introduced there. The edge is modelled as a collection of chiral Luttinger liquids. Its universal properties are contained in a symmetric and invertible integer matrix K as well as an integer charge vector Q and an integer spin vector S. The number of components of the latter vectors corresponds to the number of real chiral scalar fields that describe the edge. The component Q_i denotes the electric charge of the ith scalar field, while the component S_i denotes the spin associated with up or down projection along the $\hat{S}_z$ spin-axis which is conserved in our system.

Time-reversal symmetry implies the structure [36]

$$K = \begin{pmatrix} \kappa & \Delta \\ \Delta^\mathsf{T} & -\kappa \end{pmatrix}, \quad Q = \begin{pmatrix} \rho \\ \rho \end{pmatrix}, \qquad S = \begin{pmatrix} \rho \\ -\rho \end{pmatrix} \tag{E1}$$

in terms of matrices κ and Δ as well as a vector ρ for each spin component. Above, κ is an $N \times N$ symmetric matrix, Δ is an $N \times N$ anti-symmetric matrix, and ρ is an N-component vector. $2N$ is the number of scalar fields. Since spin is odd under time-reversal, it can be seen that in our conventions, time-reversal relates the top N components with the bottom N components.

The edge of a $\nu = -4/3$ FTI state is represented by $N = 2$ and

$$\kappa = \begin{pmatrix} -3 & 0 \\ 0 & 1 \end{pmatrix}, \quad \Delta = 0, \quad \rho = \begin{pmatrix} 1 \\ 1 \end{pmatrix}, \tag{E2}$$

i.e. in the quantum Hall analogy, in each of the Kramers paired blocks of K there is a full Landau level and a conjugate Laughlin 1/3 state to model $\nu = -2/3$ in that spin sector[1]. The data of Eq. E1 (using the parameters of Eq. E2) is therefore consistent with an FTI that is connected to the limit of a decoupled product of $\nu = -2/3$ FCIs in each spin with opposite chiralities. We can verify the vanishing charge Hall conductance, mandated by time-reversal symmetry, by computing $Q^\mathsf{T} K^{-1} Q = 0$. The 'spin filling fraction' [36] is $\frac{1}{2} Q^\mathsf{T} K^{-1} S = 2/3$, which simply reflects the fact that each spin sector individually has a Hall conductance of $\pm 2/3$ which is opposite in the two spin sectors.

We now ask whether the edge modes are stable against perturbations at the edge. For instance, if we allow all possible tunnelings of electronic charge among the different edge branches, some or all of the gapless modes could be gapped out. The answer will generally depend on what symmetries the perturbations preserve. Following the stability analysis of Ref. 36, we find that our state at $\nu = -4/3$ retains protected gapless modes as long as we impose charge U(1), spin U(1), and TR symmetry. We consider this a fractional quantum spin Hall (FQSH) state as it preserves spin U(1). Breaking spin U(1) will allow to gap out all the edge modes, but this is beyond FQSH which always assumes spin U(1). The MoTe2 samples, due to their valley-spin locking, maintain $U(1)$ symmetry. To demonstrate the above statements, we specify a possible choice of two tunneling vectors T that together would gap out all the edge modes [36]

$$T' = \begin{pmatrix} 1 \\ -1 \\ -1 \\ 1 \end{pmatrix}, \quad T'' = \begin{pmatrix} -1 \\ 3 \\ 1 \\ -3 \end{pmatrix}. \tag{E3}$$

[1] Eq. E2 corresponds to the so-called symmetric basis representation of each Kramers paired block. As shown in Ref. 100, this is equivalent to the hierarchical basis representation with $\kappa' = \begin{pmatrix} -2 & 1 \\ 1 & 1 \end{pmatrix}$ and $\rho' = (0, 1)^\mathsf{T}$. This can be related to the symmetric basis via the linear transformation $\kappa = W^\mathsf{T} \kappa' W$ and $\rho = W^\mathsf{T} \rho'$, where $W^\mathsf{T} = \begin{pmatrix} -1 & 1 \\ 0 & 1 \end{pmatrix}$.

Both tunneling vectors are charge-conserving since they satisfy $Q^\mathsf{T} T = 0$. They also satisfy time-reversal since they obey $\Sigma_1 T = -T$, where $\Sigma_1 = \begin{pmatrix} 0 & I_N \\ I_N & 0 \end{pmatrix}$ with I_N the $N \times N$ identity matrix. Using the spin vector $S^\mathsf{T} = (1, 1, -1, -1)$, one obtains that while T' is spin conserving ($S^\mathsf{T} T' = 0$), the tunneling vector T'' is not ($S^\mathsf{T} T'' \neq 0$). The latter therefore represents the gapless modes that are protected by spin U(1), charge U(1) and time reversal symmetry in a strongly interacting edge. Note that the charge per spin that is gapped out by T' is zero since $(1, -1)\rho = 0$, so that the total charge of the remaining gapless mode is $\pm 2/3$ per spin. This result will be used below in the next subsection on non-local transport measurements.

B. Landauer-Büttiker analysis

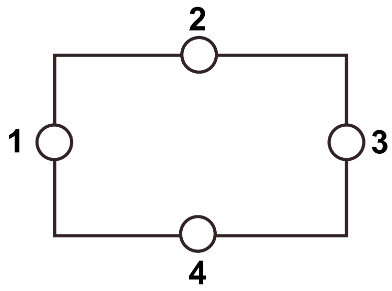

FIG. S42. **Contact configuration for the Landauer-Büttiker analysis.** The four contacts are labelled.

While the FTI lacks a non-zero quantized charge Hall response, its presence can still be detected with non-local transport measurements. In this subsection, we consider the expected values of various transport experiments using the four-contact geometry in Fig. S42. Our focus is on low temperatures and the regime where transport is mediated by the gapless edge modes of the FTI at $\nu = -4/3$. We assume perfect spin $U(1)$ symmetry (such that we have a FQSH state) where the different spins cannot scatter into each other, perfect contacts, and full local equilibration between the possible multiple edge modes for a given spin projection. Within the Landauer-Büttiker framework, we have

$$I_\alpha = \sum_\beta \mathcal{G}_{\alpha\beta} V_\beta, \quad \mathcal{G} = \begin{pmatrix} -4/3 & 2/3 & 0 & 2/3 \\ 2/3 & -4/3 & 2/3 & 0 \\ 0 & 2/3 & -4/3 & 2/3 \\ 2/3 & 0 & 2/3 & -4/3 \end{pmatrix} \tag{E4}$$

where I_α and V_α are the current and voltage on lead α respectively. We use the convention that I_α denotes the current leaving the system via lead α. We now explain how the conductance matrix $\mathcal{G}$ is determined. $\mathcal{G}$ has an entry 2/3 between any pair of adjacent contacts, and 0 for contacts that are not adjacent (every current goes through one adjacent contact to arrive to a non-adjacent one). This is because raising the voltage on lead α increases the chemical potential of its outgoing edge states which carry charge 2/3. This is collected as current in the adjacent leads. The diagonal entries are constrained by the fact that a uniform shift of all voltages should not change the current. This imposes that each row of $\mathcal{G}$ has vanishing sum. Consistent with current conservation for arbitrary voltages, each column of $\mathcal{G}$ has vanishing sum.

Eq. E4 can be solved with various constraints on I_α and V_α depending on the measurement configuration, as discussed below. We consider effective four-terminal and two-terminal measurements. The combination of these measurements would provide experimental evidence for the $\nu = -4/3$ FTI. We also note that since the FTI is incompressible, the below quantized responses should exhibit finite plateaus as a function of e.g. filling factor and displacement field.

For the four-terminal measurements, we drive a current I through two current contacts (i.e. we impose $I_\alpha = -I_\beta = I$ for some $\alpha \neq \beta$), and measure the voltage drop V across the voltage contacts (which are different than the current contacts). The resistance is determined as $R = V/I$. Because we impose the condition of zero current at the voltage leads, Eq. E4 can be solved for all the voltages up to an overall constant offset. We find, referring to Fig. S42:

- Current contacts 1 & 3; voltage contacts 2 & 4 : $R = 0$. This corresponds to vanishing Hall conductance in an FTI.
- Current contacts 1 & 4; voltage contacts 2 & 3 : $R = 3/8$

For two-terminal measurements, we need to specify the two 'terminals', where a terminal could involve shorting together several contacts, which equalizes their voltages. Other contacts not within a terminal are therefore floating, which means that they cannot source or sink any net current ($I_\alpha = 0$). As an example, if we specify terminals $(1,3)$ & 2, this means that contacts 1 and 3 are shorted together to make one terminal ($V_1 = V_3$), contact 2 forms the other terminal, and contact 4 is left floating ($I_4 = 0$). By conservation of current, we have $I_1 + I_3 = -I_2 = I$, where I is the current driven through the terminals. The conductance $G = I/V$ is computed by dividing this current by the voltage drop V across the terminals. V can be uniquely determined by solving Eq. E4 with the above constraints. This analysis can be repeated for different choices of terminals. Referring again to Fig. S42, we list the conductances G for $\nu = -4/3$ FTI using the four configurations considered in Ref. 84:

- Terminals $(1,4)$ & $(2,3)$: $G = 4/3$
- Terminals (1) & $(2,3)$: $G = 1$
- Terminals (1) & (4) : $G = 8/9$
- Terminals (1) & (3) : $G = 2/3$

[1] T. Neupert, L. Santos, C. Chamon, and C. Mudry, Phys. Rev. Lett. **106**, 236804 (2011).
[2] D. N. Sheng, Z.-C. Gu, K. Sun, and L. Sheng, Nature Communications **2**, 389 (2011), arXiv:1102.2658 [cond-mat.str-el].
[3] N. Regnault and B. A. Bernevig, Phys. Rev. X **1**, 021014 (2011).
[4] K. Sun, Z. Gu, H. Katsura, and S. Das Sarma, Phys. Rev. Lett. **106**, 236803 (2011).
[5] S. Yang, Z.-C. Gu, K. Sun, and S. Das Sarma, Phys. Rev. B **86**, 241112 (2012).
[6] E. Tang, J.-W. Mei, and X.-G. Wen, Phys. Rev. Lett. **106**, 236802 (2011).
[7] J. Cai, E. Anderson, C. Wang, X. Zhang, X. Liu, W. Holtzmann, Y. Zhang, F. Fan, T. Taniguchi, K. Watanabe, Y. Ran, T. Cao, L. Fu, D. Xiao, W. Yao, and X. Xu, Nature 10.1038/s41586-023-06289-w (2023).
[8] Y. Zeng, Z. Xia, K. Kang, J. Zhu, P. Knüppel, C. Vaswani, K. Watanabe, T. Taniguchi, K. F. Mak, and J. Shan, Nature 10.1038/s41586-023-06452-3 (2023).
[9] H. Park, J. Cai, E. Anderson, Y. Zhang, J. Zhu, X. Liu, C. Wang, W. Holtzmann, C. Hu, Z. Liu, T. Taniguchi, K. Watanabe, J.-H. Chu, T. Cao, L. Fu, W. Yao, C.-Z. Chang, D. Cobden, D. Xiao, and X. Xu, Nature **622**, 74 (2023).
[10] F. Xu, Z. Sun, T. Jia, C. Liu, C. Xu, C. Li, Y. Gu, K. Watanabe, T. Taniguchi, B. Tong, J. Jia, Z. Shi, S. Jiang, Y. Zhang, X. Liu, and T. Li, Phys. Rev. X **13**, 031037 (2023).
[11] H. Park, J. Cai, E. Anderson, X.-W. Zhang, X. Liu, W. Holtzmann, W. Li, C. Wang, C. Hu, Y. Zhao, T. Taniguchi, K. Watanabe, J. Yang, D. Cobden, J.-H. Chu, N. Regnault, B. A. Bernevig, L. Fu, T. Cao, D. Xiao, and X. Xu, Ferromagnetism and topology of the higher flat band in a fractional chern insulator (2024), arXiv:2406.09591 [cond-mat.mes-hall].
[12] F. Xu, X. Chang, J. Xiao, Y. Zhang, F. Liu, Z. Sun, N. Mao, N. Peshcherenko, J. Li, K. Watanabe, T. Taniguchi, B. Tong, L. Lu, J. Jia, D. Qian, Z. Shi, Y. Zhang, X. Liu, S. Jiang, and T. Li, Interplay between topology and correlations in the second moiré band of twisted bilayer mote2 (2024), arXiv:2406.09687 [cond-mat.mes-hall].
[13] H. Li, U. Kumar, K. Sun, and S.-Z. Lin, Phys. Rev. Res. **3**, L032070 (2021).
[14] V. Crépel and L. Fu, Phys. Rev. B **107**, L201109 (2023).
[15] N. Morales-Durán, J. Wang, G. R. Schleder, M. Angeli, Z. Zhu, E. Kaxiras, C. Repellin, and J. Cano, Pressure–enhanced fractional chern insulators in moiré transition metal dichalcogenides along a magic line (2023), arXiv:2304.06669 [cond-mat.str-el].
[16] C. Wang, X.-W. Zhang, X. Liu, Y. He, X. Xu, Y. Ran, T. Cao, and D. Xiao, Fractional chern insulator in twisted bilayer mote$_2$ (2023), arXiv:2304.11864 [cond-mat.str-el].
[17] A. P. Reddy and L. Fu, Toward a global phase diagram of the fractional quantum anomalous hall effect (2023), arXiv:2308.10406 [cond-mat.mes-hall].
[18] A. P. Reddy, F. Alsallom, Y. Zhang, T. Devakul, and L. Fu, Phys. Rev. B **108**, 085117 (2023).
[19] J. Yu, J. Herzog-Arbeitman, M. Wang, O. Vafek, B. A. Bernevig, and N. Regnault, Phys. Rev. B **109**, 045147 (2024).
[20] C. Xu, J. Li, Y. Xu, Z. Bi, and Y. Zhang, Proceedings of the National Academy of Sciences **121**, e2316749121 (2024), https://www.pnas.org/doi/pdf/10.1073/pnas.2316749121.
[21] A. Abouelkomsan, A. P. Reddy, L. Fu, and E. J. Bergholtz, Band mixing in the quantum anomalous hall regime of twisted semiconductor bilayers (2023), arXiv:2309.16548 [cond-mat.mes-hall].
[22] Y. Jia, J. Yu, J. Liu, J. Herzog-Arbeitman, Z. Qi, N. Regnault, H. Weng, B. A. Bernevig, and Q. Wu, Moiré fractional chern insulators i: First-principles calculations and continuum models of twisted bilayer mote$_2$ (2023), arXiv:2311.04958 [cond-mat.mes-hall].
[23] N. Mao, C. Xu, J. Li, T. Bao, P. Liu, Y. Xu, C. Felser, L. Fu, and Y. Zhang, Lattice relaxation, electronic structure and continuum model for twisted bilayer mote$_2$ (2023), arXiv:2311.07533 [cond-mat.str-el].
[24] X.-W. Zhang, C. Wang, X. Liu, Y. Fan, T. Cao, and D. Xiao, Polarization-driven band topology evolution in twisted mote$_2$ and wse$_2$ (2024), arXiv:2311.12776 [cond-mat.mtrl-sci].
[25] T. Wang, M. Wang, W. Kim, S. G. Louie, L. Fu, and M. P. Zaletel, Topology, magnetism and charge order in twisted mote2 at higher integer hole fillings (2023), arXiv:2312.12531 [cond-mat.str-el].
[26] T. Han, Z. Lu, G. Scuri, J. Sung, J. Wang, T. Han, K. Watanabe, T. Taniguchi, H. Park, and L. Ju, Nature Nanotechnology 10.1038/s41565-023-01520-1 (2023).
[27] Z. Lu, T. Han, Y. Yao, A. P. Reddy, J. Yang, J. Seo, K. Watanabe, T. Taniguchi, L. Fu, and L. Ju, Nature **626**, 759 (2024).
[28] J. Dong, T. Wang, T. Wang, T. Soejima, M. P. Zaletel, A. Vishwanath, and D. E. Parker, Anomalous hall crystals in rhombohedral multilayer graphene i: Interaction-driven chern bands and fractional quantum hall states at zero magnetic field (2023), arXiv:2311.05568 [cond-mat.str-el].
[29] B. Zhou, H. Yang, and Y.-H. Zhang, Fractional quantum anomalous hall effects in rhombohedral multilayer graphene in the moiréless limit and in coulomb imprinted superlattice (2023), arXiv:2311.04217 [cond-mat.str-el].
[30] Z. Dong, A. S. Patri, and T. Senthil, Theory of fractional quantum anomalous hall phases in pentalayer rhombohedral graphene moiré structures (2023), arXiv:2311.03445 [cond-mat.str-el].
[31] J. Herzog-Arbeitman, Y. Wang, J. Liu, P. M. Tam, Z. Qi, Y. Jia, D. K. Efetov, O. Vafek, N. Regnault, H. Weng, *et al.*, arXiv preprint arXiv:2311.12920 (2023).
[32] Y. H. Kwan, J. Yu, J. Herzog-Arbeitman, D. K. Efetov, N. Regnault, and B. A. Bernevig, Moiré fractional chern insulators iii: Hartree-fock phase diagram, magic angle regime for chern insulator states, the role of the moiré potential and goldstone gaps in rhombohedral graphene superlattices (2023), arXiv:2312.11617 [cond-mat.str-el].
[33] Z. Guo, X. Lu, B. Xie, and J. Liu, Theory of fractional chern insulator states in pentalayer graphene moiré superlattice (2023), arXiv:2311.14368 [cond-mat.str-el].
[34] B. A. Bernevig and S.-C. Zhang, Phys. Rev. Lett. **96**, 106802 (2006).

[35] M. Levin and A. Stern, Phys. Rev. Lett. **103**, 196803 (2009).
[36] T. Neupert, L. Santos, S. Ryu, C. Chamon, and C. Mudry, Phys. Rev. B **84**, 165107 (2011).
[37] A. Stern, Annual Review of Condensed Matter Physics **7**, 349 (2016), https://doi.org/10.1146/annurev-conmatphys-031115-011559.
[38] T. Neupert, C. Chamon, T. Iadecola, L. H. Santos, and C. Mudry, Physica Scripta **2015**, 014005 (2015).
[39] M. Levin and A. Stern, Phys. Rev. B **86**, 115131 (2012).
[40] K. Kang, B. Shen, Y. Qiu, Y. Zeng, Z. Xia, K. Watanabe, T. Taniguchi, J. Shan, and K. F. Mak, Nature **628**, 522 (2024).
[41] C. Repellin, B. A. Bernevig, and N. Regnault, Phys. Rev. B **90**, 245401 (2014).
[42] S. Furukawa and M. Ueda, Phys. Rev. A **90**, 033602 (2014).
[43] V. Crépel and N. Regnault, Attractive haldane bilayers for trapping non-abelian anyons (2024), arXiv:2403.05622 [cond-mat.str-el].
[44] H. Chen and K. Yang, Phys. Rev. B **85**, 195113 (2012).
[45] S. Mukherjee and K. Park, Phys. Rev. B **99**, 115131 (2019).
[46] N. Bultinck, S. Chatterjee, and M. P. Zaletel, Phys. Rev. Lett. **124**, 166601 (2020).
[47] S. Furukawa and M. Ueda, Phys. Rev. A **90**, 033602 (2014).
[48] Y.-H. Zhang, Composite fermion insulator in opposite-fields quantum hall bilayers (2018), arXiv:1810.03600 [cond-mat.str-el].
[49] Y. H. Kwan, Y. Hu, S. H. Simon, and S. A. Parameswaran, Phys. Rev. Lett. **126**, 137601 (2021).
[50] Y. H. Kwan, Y. Hu, S. H. Simon, and S. A. Parameswaran, Phys. Rev. B **105**, 235121 (2022).
[51] P. M. Eugenio and C. B. Dağ, SciPost Phys. Core **3**, 015 (2020).
[52] N. Stefanidis and I. Sodemann, Phys. Rev. B **102**, 035158 (2020).
[53] S. Chatterjee, M. Ippoliti, and M. P. Zaletel, Phys. Rev. B **106**, 035421 (2022).
[54] N. Myerson-Jain, C.-M. Jian, and C. Xu, The conjugate composite fermi liquid (2023), arXiv:2311.16250 [cond-mat.str-el].
[55] K. Yang, Chinese Physics B **32**, 097303 (2023).
[56] Y.-M. Wu, D. Shaffer, Z. Wu, and L. H. Santos, Phys. Rev. B **109**, 115111 (2024).
[57] Z. D. Shi, H. Goldman, Z. Dong, and T. Senthil, Excitonic quantum criticality: from bilayer graphene to narrow chern bands (2024), arXiv:2402.12436 [cond-mat.str-el].
[58] Y. H. Kwan, Z. Wang, G. Wagner, S. H. Simon, S. A. Parameswaran, and N. Bultinck, Textured exciton insulators (2024), arXiv:2406.15343 [cond-mat.str-el].
[59] B. A. Bernevig and N. Regnault, Phys. Rev. B **85**, 075128 (2012).
[60] N. S. Rytova, arXiv preprint arXiv:1806.00976 (2018).
[61] L. V. Keldysh, Soviet Journal of Experimental and Theoretical Physics Letters **29**, 658 (1979).
[62] P. Cudazzo, I. V. Tokatly, and A. Rubio, Phys. Rev. B **84**, 085406 (2011).
[63] G. Wang, A. Chernikov, M. M. Glazov, T. F. Heinz, X. Marie, T. Amand, and B. Urbaszek, Rev. Mod. Phys. **90**, 021001 (2018).
[64] S. Zhao, J. Huang, V. Crépel, X. Wu, T. Zhang, H. Wang, X. Han, Z. Li, C. Xi, S. Pan, Z. Wang, K. Watanabe, T. Taniguchi, B. Sacépé, J. Zhang, N. Wang, J. Lu, N. Regnault, and Z. V. Han, Probing the fractional quantum hall phases in valley-layer locked bilayer mos_2 (2023), arXiv:2308.02821 [cond-mat.mes-hall].
[65] F. Wu, T. Lovorn, E. Tutuc, I. Martin, and A. H. MacDonald, Phys. Rev. Lett. **122**, 086402 (2019).
[66] A. Laturia, M. L. Van de Put, and W. G. Vandenberghe, npj 2D Materials and Applications **2**, 6 (2018).
[67] A. M. Läuchli, Z. Liu, E. J. Bergholtz, and R. Moessner, Phys. Rev. Lett. **111**, 126802 (2013).
[68] E. H. Rezayi and S. H. Simon, Phys. Rev. Lett. **106**, 116801 (2011).
[69] Z. Liu, R. N. Bhatt, and N. Regnault, Phys. Rev. B **91**, 045126 (2015).
[70] R. Roy, Phys. Rev. B **90**, 165139 (2014).
[71] G. D. Wilk, R. M. Wallace, and J. M. Anthony, Journal of Applied Physics **89**, 5243 (2001), https://pubs.aip.org/aip/jap/article-pdf/89/10/5243/19314247/5243_1_online.pdf.
[72] A. Blason and M. Fabrizio, Phys. Rev. B **106**, 235112 (2022).
[73] Y. H. Kwan, G. Wagner, N. Bultinck, S. H. Simon, E. Berg, and S. A. Parameswaran, Electron-phonon coupling and competing kekulé orders in twisted bilayer graphene (2023), arXiv:2303.13602 [cond-mat.str-el].
[74] H. Shi, W. Miao, and X. Dai, Moiré optical phonons dancing with heavy electrons in magic-angle twisted bilayer graphene (2024), arXiv:2402.11824 [cond-mat.mes-hall].
[75] M. P. Zaletel, R. S. K. Mong, F. Pollmann, and E. H. Rezayi, Phys. Rev. B **91**, 045115 (2015).
[76] L. Herviou and F. Mila, Phys. Rev. B **107**, 115137 (2023).
[77] S. H. Simon and E. H. Rezayi, Phys. Rev. B **87**, 155426 (2013).
[78] M. R. Peterson and C. Nayak, Phys. Rev. B **87**, 245129 (2013).
[79] W. Bishara and C. Nayak, Phys. Rev. B **80**, 121302 (2009).
[80] C. Xu, N. Mao, T. Zeng, and Y. Zhang, Multiple chern bands in twisted $mote_2$ and possible non-abelian states (2024), arXiv:2403.17003 [cond-mat.str-el].
[81] C.-E. Ahn, W. Lee, K. Yananose, Y. Kim, and G. Y. Cho, First landau level physics in second moiré band of 2.1° twisted bilayer $mote_2$ (2024), arXiv:2403.19155 [cond-mat.str-el].
[82] C. Wang, X.-W. Zhang, X. Liu, J. Wang, T. Cao, and D. Xiao, Higher landau-level analogues and signatures of non-abelian states in twisted bilayer $mote_2$ (2024), arXiv:2404.05697 [cond-mat.str-el].
[83] Z. Ji, H. Park, M. E. Barber, C. Hu, K. Watanabe, T. Taniguchi, J.-H. Chu, X. Xu, and Z. xun Shen, Local probe of bulk and edge states in a fractional chern insulator (2024), arXiv:2404.07157 [cond-mat.str-el].
[84] K. Kang, B. Shen, Y. Qiu, K. Watanabe, T. Taniguchi, J. Shan, and K. F. Mak, Observation of the fractional quantum spin hall effect in moiré mote2 (2024), arXiv:2402.03294 [cond-mat.mes-hall].
[85] Y.-H. Zhang, Vortex spin liquid with fractional quantum spin hall effect in moiré chern bands (2024), arXiv:2402.05112 [cond-mat.str-el].
[86] J. May-Mann, A. Stern, and T. Devakul, Theory of

half-integer fractional quantum spin hall insulator edges (2024), arXiv:2403.03964 [cond-mat.mes-hall].
[87] C.-M. Jian, M. Cheng, and C. Xu, Minimal fractional topological insulator in half-filled conjugate moiré chern bands (2024), arXiv:2403.07054 [cond-mat.str-el].
[88] I. S. Villadiego, Halperin states of particles and holes in ideal time reversal invariant pairs of chern bands and the fractional quantum spin hall effect in moiré mote$_2$ (2024), arXiv:2403.12185 [cond-mat.mes-hall].
[89] Y.-H. Zhang, Non-abelian and abelian descendants of vortex spin liquid: fractional quantum spin hall effect in twisted mote$_2$ (2024), arXiv:2403.12126 [cond-mat.str-el].
[90] Y.-Z. Chou and S. D. Sarma, Composite helical edges from abelian fractional topological insulators (2024), arXiv:2406.06669 [cond-mat.str-el].
[91] A. Abouelkomsan and L. Fu, Non-abelian spin hall insulator (2024), arXiv:2406.14617 [cond-mat.mes-hall].
[92] F. D. M. Haldane, Phys. Rev. Lett. **55**, 2095 (1985).
[93] M. O. Goerbig, R. Moessner, and B. Douçot, Phys. Rev. B **74**, 161407 (2006).
[94] H. Lee, S. Poncé, K. Bushick, S. Hajinazar, J. Lafuente-Bartolome, J. Leveillee, C. Lian, J.-M. Lihm, F. Macheda, H. Mori, *et al.*, npj Computational Materials **9**, 156 (2023).
[95] H. Miyazaki, S. Odaka, T. Sato, S. Tanaka, H. Goto, A. Kanda, K. Tsukagoshi, Y. Ootuka, and Y. Aoyagi, Applied Physics Express **1**, 034007 (2008).
[96] T. Ma, H. Chen, K. Yananose, X. Zhou, L. Wang, R. Li, Z. Zhu, Z. Wu, Q.-H. Xu, J. Yu, *et al.*, Nature Communications **13**, 5465 (2022).
[97] T. C. Berkelbach, M. S. Hybertsen, and D. R. Reichman, Phys. Rev. B **88**, 045318 (2013).
[98] A. Chernikov, T. C. Berkelbach, H. M. Hill, A. Rigosi, Y. Li, B. Aslan, D. R. Reichman, M. S. Hybertsen, and T. F. Heinz, Phys. Rev. Lett. **113**, 076802 (2014).
[99] D. Van Tuan, M. Yang, and H. Dery, Phys. Rev. B **98**, 125308 (2018).
[100] L. Santos, T. Neupert, S. Ryu, C. Chamon, and C. Mudry, Phys. Rev. B **84**, 165138 (2011)
